# Synthesis and Self‐Assembly of a Discotic Oligo‐Carboxylate *Tetra‐*Porphyrin‐Perylenebisimide Amphiphile

**DOI:** 10.1002/chem.202502093

**Published:** 2025-07-22

**Authors:** Erik J. Schulze, Mingjian Wu, Christina Hofmann, Erdmann Spiecker, Andreas Hirsch

**Affiliations:** ^1^ Department of Chemistry & Pharmacy Chair of Organic Chemistry II Friedrich‐Alexander‐Universität Erlangen‐Nürnberg Nikolaus‐Fiebiger‐Straße 10 91058 Erlangen Germany; ^2^ Institute of Micro‐ and Nanostructure Research (IMN) Center for Nanoanalysis and Electron Microscopy (CENEM) and Interdisciplinary Center for Nanostructured Films (IZNF) Friedrich‐Alexander‐Universität Erlangen‐Nürnberg Cauerstraße 3 91058 Erlangen Germany

**Keywords:** aggregation, amphiphiles, donor‐acceptor‐hybrids, perylenebisimide, porphyrinoids

## Abstract

We report the synthesis of an amphiphilic *tetra*‐porphyrin perylenebisimide (PBI) conjugate via four‐fold Suzuki cross‐coupling at the PBI *ortho‐*position. Oligo‐carboxylate Newkome‐type G1 dendrons attached at the porphyrin periphery serve as polar head groups. Theoretical modelling corroborated the assumed planar discotic geometry. The amphiphile is very soluble in mixtures of basic water and THF, where it displays strong aggregation at high water content. Insights into the self‐assembly process were obtained by tracking the individualization through THF addition using UV/Vis absorption spectroscopy. Here, a PBI‐centred aggregation motif becomes evident. Further evaluation of the morphology via STEM and TEM imaging reveals that the assembly is guided by hydrophobic interactions as well as interactions of the carboxylates. These *ortho*‐linked *tetra*‐porphyrin PBI architectures fill a gap within the known families of porphyrin‐PBI conjugates. Furthermore, the amphiphile is a valuable addition to the library of amphiphilic porphyrin‐PBI conjugates, representing a discotic counterpart to the known calamitic architectures.

## Graphical abstract

We report on the synthesis of a new amphiphilic *tetra*‐*ortho‐*porphyrin PBI pentad. The pentads are characterized by quenching of the fluorescence emission, indicating efficient photo‐induced charge‐transfer. The amphiphiles show solubility in basic aqueous solutions of THF, where high water contents lead to a PBI‐centered aggregation. TEM imaging reveals the morphology as randomly coiled sheets consisting out of highly regular assemblies.

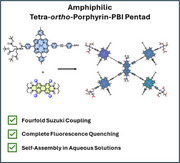

## 1 Introduction

Organic materials for efficient and sustainable light‐to‐energy conversion containing integrated donor‐acceptor (D‐A) couples have attracted considerable attention over the last decades.^[^ ^1^, ^2^ ^]^ Such systems, capable of photoinduced energy‐ or charge‐transfer, are of high interest for solar‐cell applications,^[^ ^3^ ^]^ photo‐catalytic systems,^[^ ^4^ ^]^ and artificial photosynthesis.^[^ ^5^, ^6^, ^7^ ^]^ Organic chromophores with extended *π*‐systems are of particular interest, because they can be precisely fine‐tuned at the molecular level. However, gaining control over the supramolecular assembly of such systems is of utmost importance as well, as it is detrimental to the bulk properties. When intermolecular donor‐acceptor interactions dictate the assembly, unwanted segregated stacking motifs are often formed. This can result in the suppression of long‐lived charge‐separated states and high charge mobility by fast charge recombination pathways.^[^ ^1^ ^]^ One approach to overcome these interactions is controlling the self‐assembly through the utilization of the hydrophobic effect, that is, designing amphiphiles with integrated donor‐acceptor systems.^[^ ^8^, ^9^ ^]^ However, this attractive approach is still challenging regarding the synthesis and the defined supramolecular aggregation of the molecular building blocks.^[^ ^10^, ^11^, ^12^ ^]^ Therefore, to gain further insights into the self‐assembly and structure‐to‐function principles of amphiphilic D‐A systems, using photo‐physically well‐understood D‐A pairs, such as porphyrins and rylenebisimides, is highly advantageous.^[^ ^13^, ^14^, ^15^, ^16^ ^]^

**Figure 1:**
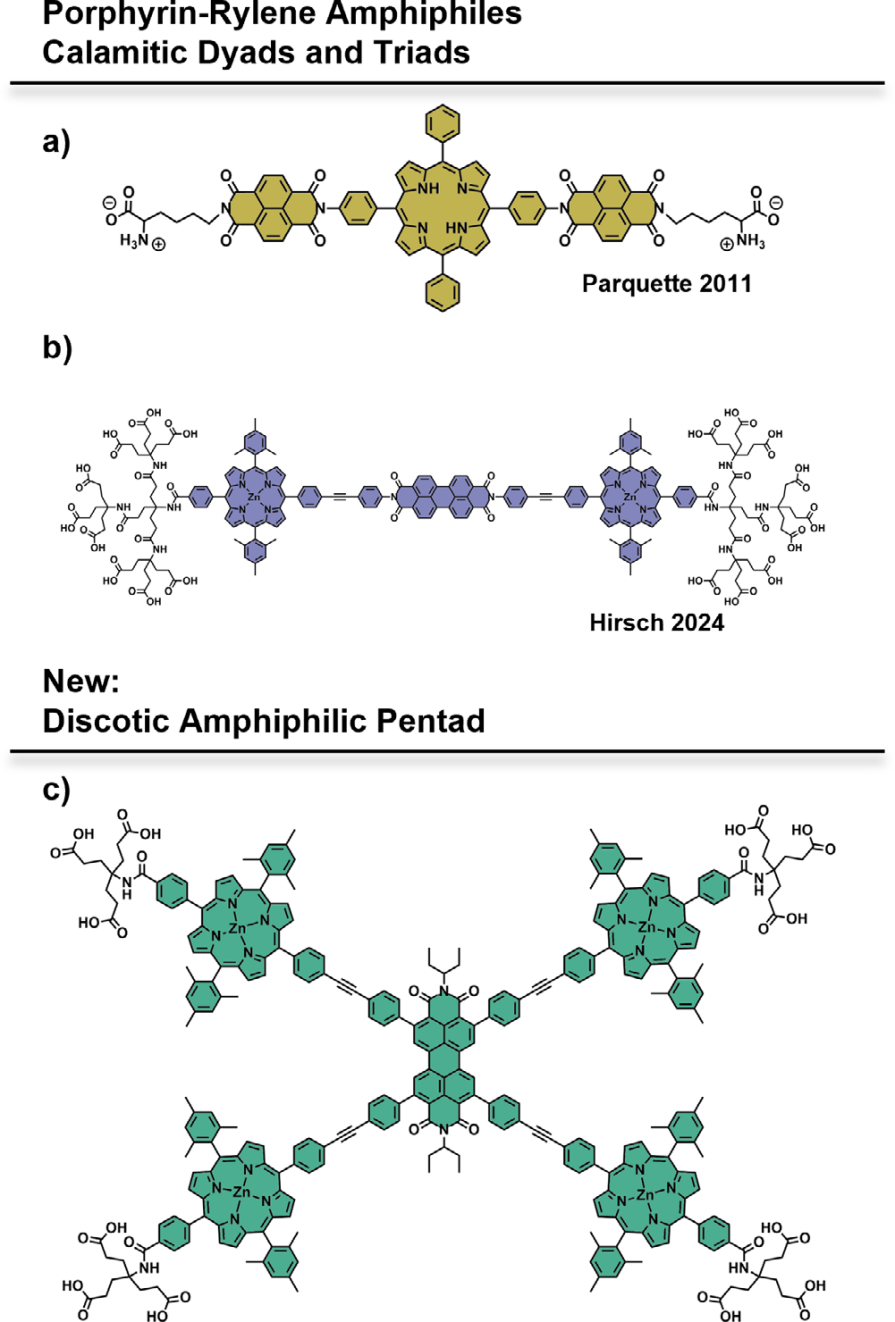
Molecular structures of a) calamitic naphthalenebisimide‐porphyrin bola‐amphiphile; b) calamitic porphyrin‐PBI bola‐amphiphile; c) discotic *tetra*‐porphyrin‐PBI amphiphile.

Furthermore, these chromophores are well‐established synthetically and can easily be functionalized in their periphery.^[^ ^17^, ^18^, ^19^, ^20^ ^]^ Building on this, the group of Parquette reported the first amphiphilic porphyrin‐rylene conjugates (Figure 1a) in 2013.^[^ ^12^, ^21^ ^]^ They synthesized a bola‐amphiphilic A‐D‐A naphthalenebisimide‐porphyrin triad, attaching amino acids as polar terminal groups. These triads assembled into discrete bicontinuous nanotubular structures from a 10 vol% MeOH in H_2_O solution. Time‐resolved spectroscopy revealed a strong dependency of the charge transfer and recombination on the assembly motif. This demonstrated the viability of the amphiphilic porphyrin‐rylene architecture in building opto‐electronically distinct structures in aqueous media.

Recently, we reported the synthesis of a family of calamitic porphyrin‐PBI amphiphiles (Figure 1b).^[^ ^22^, ^23^ ^]^ In contrast to the terminal amino acids, the highly polar Newkome‐type dendrimer head groups provided full water solubility. Tracing the individualization of amphiphiles through UV/Vis absorption spectroscopy revealed, for example, PBI‐centered aggregation motifs rotated around an axis perpendicular to the PBI plane. Furthermore, strong fluorescence quenching in the aggregated state indicated efficient charge transfer. However, the linear architecture of the amphiphiles allows for the formation of numerous superstructures that are highly dependent on the solvent environment.

To overcome this, further expansion of the geometric scope of porphyrin‐PBI architectures to *tetra*‐*ortho*‐porphyrin PBI pentads **8** and **15** (Figure 1c) – as the discotic counterpart to the reported calamitic molecules–could open the door to distinct, highly ordered columnar assemblies. Although porphyrin‐PBI conjugates linked through the imide‐ and bay‐positions are well established,^[^ ^13^, ^14^, ^15^, ^24^, ^25^, ^26^ ^]^ linkage through the *ortho‐*position is, to the best of our knowledge, still unprecedented. Therefore, we aimed to synthesize an *ortho*‐linked *tetra* porphyrin‐PBI pentad to access the sought‐after geometry, while simultaneously filling a gap in porphyrin‐PBI conjugates. Additionally, we addressed the challenge of synthesizing the respective amphiphilic pentad (Figure 1c) using well‐established oligo‐carboxylate Newkome dendrons^[^ ^27^, ^28^, ^29^ ^]^ to obtain first insights into the self‐assembly properties of this new scaffold.

## 2 Results and Discussion

### 2.1 Synthesis

**Scheme 1:**
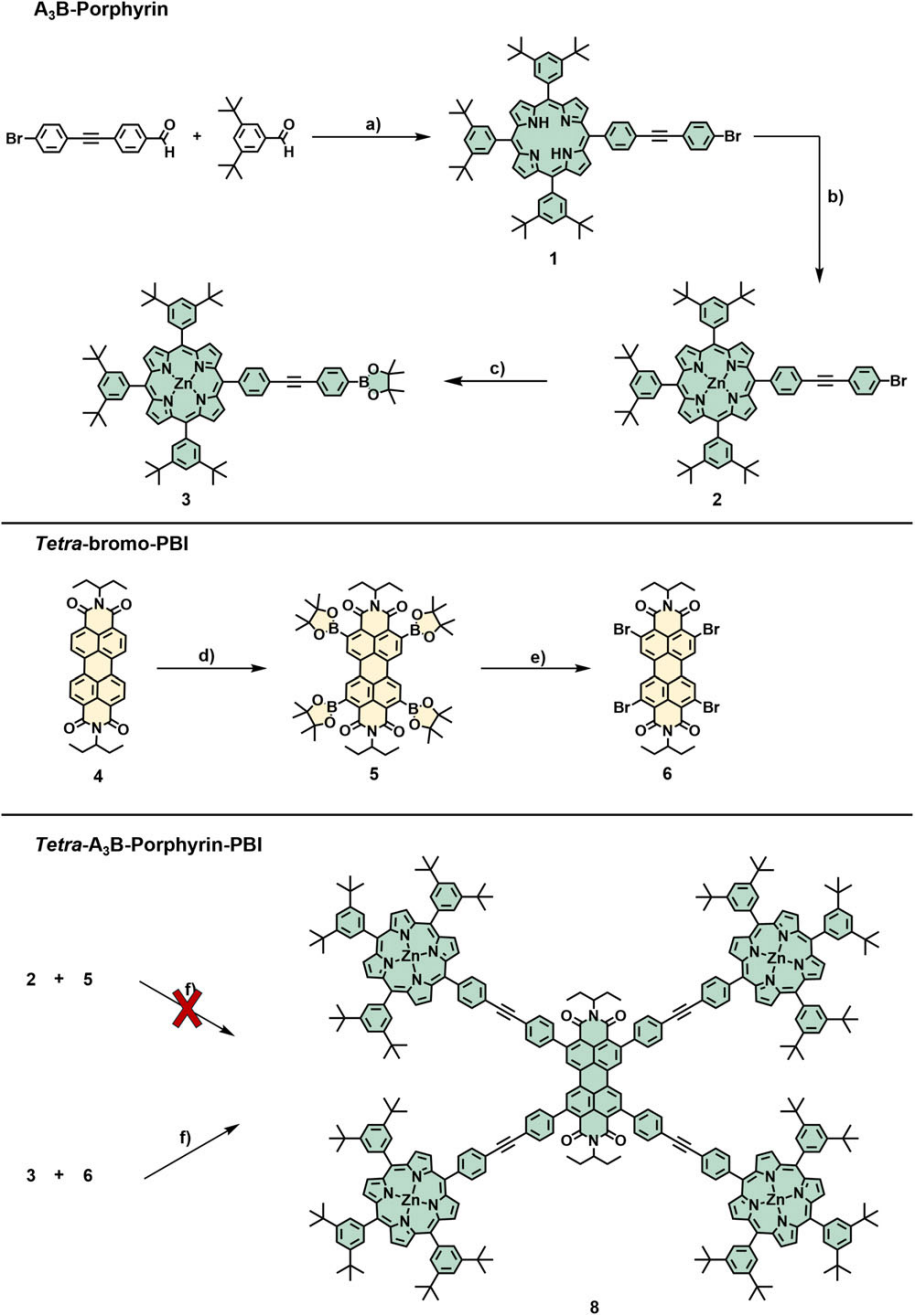
Synthetic route toward the *tetra*‐A_3_B‐porphyrin PBI and the respective precursors; a) 1: I_2_, DCM, 5 min, 40 °C, MW‐radiation; 2: *para*‐chloranil, 20 min, 40 °C, 5.9%; b) Zn(OAc)_2_, MeOH/CHCl_3_, reflux, 2.5 h, 99%; c) B_2_Pin_2_, KOAc, Pd(dppf)Cl_2_, 1,4‐dioxane, 90 °C, 5 d, 82%; d–e) following literature procedures,^[^ ^26^, ^27^ ^]^ 61% (**5**), 82% (**6**); f) Pd(PPh_3_)_4_, Cs_2_CO_3_, Tol/DMF, 80 °C, 16 h, 21%.

**Figure 2:**
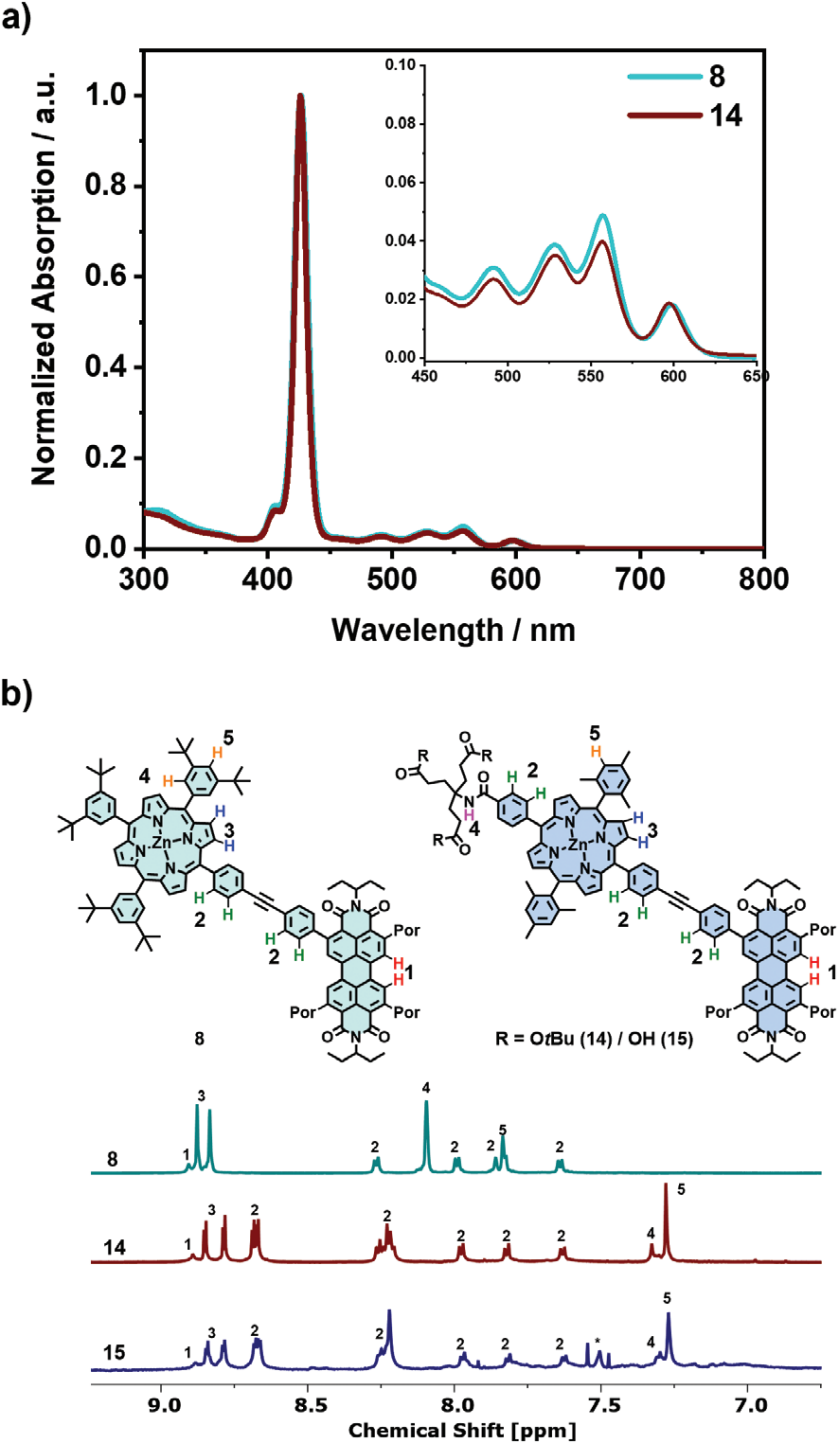
a) Normalized UV/Vis absorption spectra of **8** and **14** in THF; b) aromatic region of the ^1^H NMR of top: di‐*tert*‐butyl phenyl‐pentad **8**, middle: the *tert*‐butyl‐ester pentad **14**, amphiphilic pentad **15** (The asterisk marks a trace impurity).

We conceptualized the synthetic protocol by considering the targeted flat geometry with a possible co‐planar orientation of the porphyrin and PBI subunits. Therefore, we chose biphenyl acetylene (tolane) as the linker, which also has the benefit of comparability to our previous bola‐amphiphilic system (Figure 1b) in terms of the D‐A distance.^[^ ^22^ ^]^ The connection of the chromophores is thought to be realized by Suzuki coupling. This approach minimizes the number of fourfold reactions, which we anticipated to be the bottleneck of the synthesis. For this strategy, recent reports on the direct C─H *ortho*‐functionalization of PBIs (borylation/halogenation) have provided an ideal starting point.^[^ ^30^, ^31^ ^]^ To establish the conditions for Suzuki coupling and to gain first insights into the *ortho*‐porphyrin‐PBI conjugates, we first targeted a non‐amphiphilic pentad bearing 3,5‐di(*tert‐*butyl)phenyl substituents at the *meso‐*positions of the porphyrins. For this synthesis (Scheme 1), the primary free‐base porphyrin **1** was obtained by statistical synthesis from the respective aldehydes and pyrrole in 6% yield. Subsequent metalation with Zn(OAc)_2_ in MeOH/CHCl_3_ gave the desired porphyrin **2** in excellent yield. The initial reaction with 5 equiv. porphyrin **2** and the suitable borylated‐PBI **5** with Pd(PPh_3_)_4_ as the catalyst and Cs_2_CO_3_ as the base in DMF/toluene mixtures only yielded the desired pentad in trace amounts. Therefore, bromo porphyrin **2** was transformed into the respective boronic ester **3** via a Miyaura reaction using Pd(dppf)_2_Cl_2_ as the catalyst and KOAc as the base in 1,4‐dioxane. The reaction proceeded very slowly, despite heating to 110 °C. Nevertheless, full conversion to **3** was observed after 5 days. The borylated PBI was transformed into the respective bromo‐PBI **6** via copper(II) catalysis.^[^ ^30^ ^]^ Subjecting **3** and **6** to the same Suzuki conditions yielded the desired pentad **8** in 23% yield. Purification was achieved by size‐exclusion gel‐permeation chromatography and standard column chromatography on silica gel using toluene/THF mixtures as the mobile phase. The obtained pentad is very soluble in common organic solvents, giving rise to deep red solutions. Compound **8** was unambiguously characterized by ^1^H NMR (Figure 2b) and ^13^C NMR as well as high‐resolution mass spectrometry (HRMS) (Supporting Information).

**Scheme 2:**
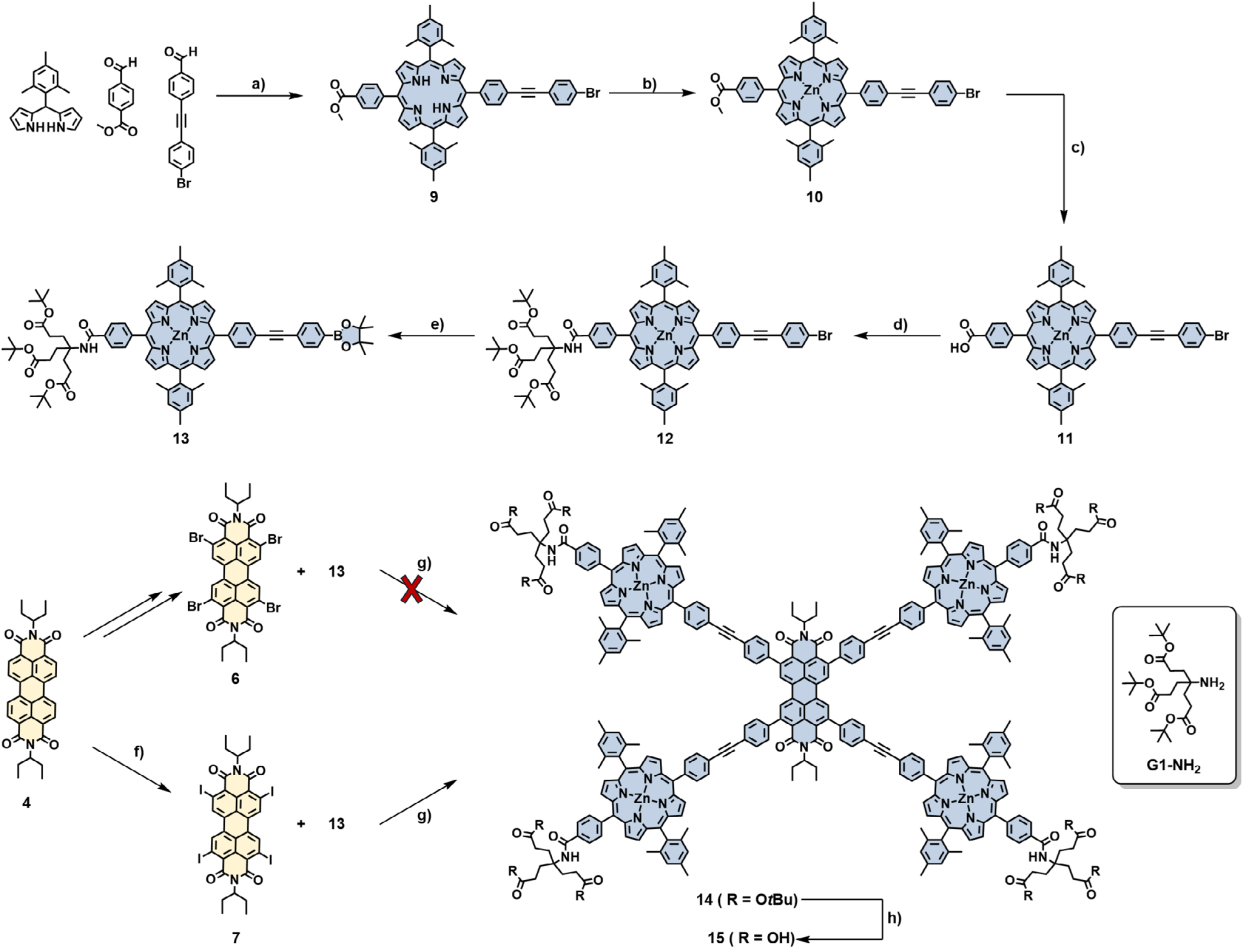
Overview over the synthetic route toward the amphiphilic pentad; a) 1: BF_3_•Et_2_O, CHCl_3_,/EtOH, rt, 2 hours; 2: DDQ, rt, 75 minutes, 18%; b) Zn(OAc)_2_, MeOH/CHCl_3_, reflux, 1:45 hour, 99%; c) LiOH, THF/H_2_O, rt., 22 hours, 99%; d) G1‐NH_2_, HOBt•H_2_O, DCC, DMF, rt., 7 days, 38%; e) B_2_Pin_2_, KOAc, Pd(dppf)Cl_2_, 1,4‐dioxane, 90 °C, 5 days, 87%; f) According to literature procedure,^[^ ^28^ ^]^ 16%; g) Pd(PPh_3_)_4_, Cs_2_CO_3_, Tol/DMF, 60 °C, 16 hours, 44%; h) 1: HCOOH, rt., 3d, 2: Zn(OAc)_2_, THF, reflux, 48 hours, 46%.

These conditions in hand, we pursued the synthesis of the respective amphiphile **15** (Scheme 2). As oligo‐carboxylate G1‐Newkome dendrons were envisioned as the polar head group, the synthesis of a *trans*‐AB_2_C porphyrin is necessary. Consequently, porphyrin **9** was synthesized under semi‐statistical [2+2]‐conditions in 18% yield. Here, mesityl groups were used as *meso*‐substituents, as they are less prone to scrambling under these conditions. Metalation with Zn(OAc)_2_ yielded **10** quantitatively, and subsequent saponification of the methyl ester with LiOH gave carboxylic acid porphyrin **11** in very good yields. To attach the Newkome G1 dendron, amide coupling with DCC and HOBt·H_2_O as the coupling agents, afforded the dendronized porphyrin **12** in 38% yield. As expected, Suzuki coupling of bromo‐porphyrin **12** and *tetra*‐borylated PBI **5** did not yield the desired pentad. Consequently, bromo G1‐porphyrin **12** was borylated under the aforementioned Miyaura conditions, with an isolated yield of 87%. Interestingly, the purification of **13** from excess B_2_Pin_2_ was more delicate than for **3**, due to the much faster hydrolysis and protodeborylation of the boronic ester. Regrettably, employing 5 equiv. of the borylated porphyrin **13** under the above‐mentioned Suzuki reaction conditions with *tetra*‐bromo PBI **6** only gave **14** in traces, with the threefold coupled and once de‐halogenated species observed as the main product by MALDI‐MS. Consequently, to achieve faster coupling under milder conditions, *tetra*‐iodo PBI was chosen, which was obtained following a literature procedure.^[^ ^32^ ^]^ Performing the Suzuki coupling of porphyrin **13** with *tetra*‐iodo PBI **7** with the previously mentioned catalytic system at 60 °C afforded higher amounts of the pentad as judged by MALDI‐MS after 16 hours. Purification was achieved by size‐exclusion column chromatography (CHCl_3_ and toluene on BioBeads SX1) followed by silica column chromatography, yielding the desired *tetra*‐dendronized pentad **14** in 44% yield. The pentad displays good solubility in common organic solvents, affording deep red solutions. Compound **14** was fully characterized by NMR spectroscopy (Figure 2b and Figures S34–S37, as well as HRMS (Figure S73). To obtain the desired amphiphile, the cleavage of the *tert‐*butyl esters was performed under acidic conditions. Using neat formic acid at room temperature yielded the desired crude amphiphile **15**. Under these acidic conditions, the partial demetallation of the zinc is observed. Therefore, the crude pentad was re‐metalated using 8 eq. Zn(OAc)_2_ in THF at 70 °C. The re‐metalation was monitored by tracking the characteristic absorption band of the free‐base species at 650 nm by UV/Vis spectroscopy. The thereby formed re‐metalated precipitate still contains networks formed by complexation of excess zinc ions. Therefore, the by filtration obtained solid was redissolved in THF, followed by careful acidification with TFA (1 vol%) and immediate precipitation with pentane. This furnished the fully metalated and protonated pentad **15** in 46% yield. It should be noted, that the low yield is mainly due to losses during the purification procedure, while the reaction proceeds to full conversion. The deprotected pentad **15** is very soluble in basic aqueous and slightly acidic THF solutions. **15** was characterized by ^1^H NMR spectroscopy (Figure 2b) and HRMS (Figure S74). As NMR measurements had to be conducted in mixtures of THF*d_8_* and TFA‐d, which over time led to the demetallation of the porphyrin, no ^13^C NMR of pure **15** could be recorded (Figures S39 and S40). The ^1^H NMR spectra (Figure 2b) show all the desired signals in the aromatic region, and the absence of the *tert‐*butyl‐ester signals at 1.47 ppm (Figure S38).

### 2.2 Steady‐State Absorption Spectroscopy

**Figure 3:**
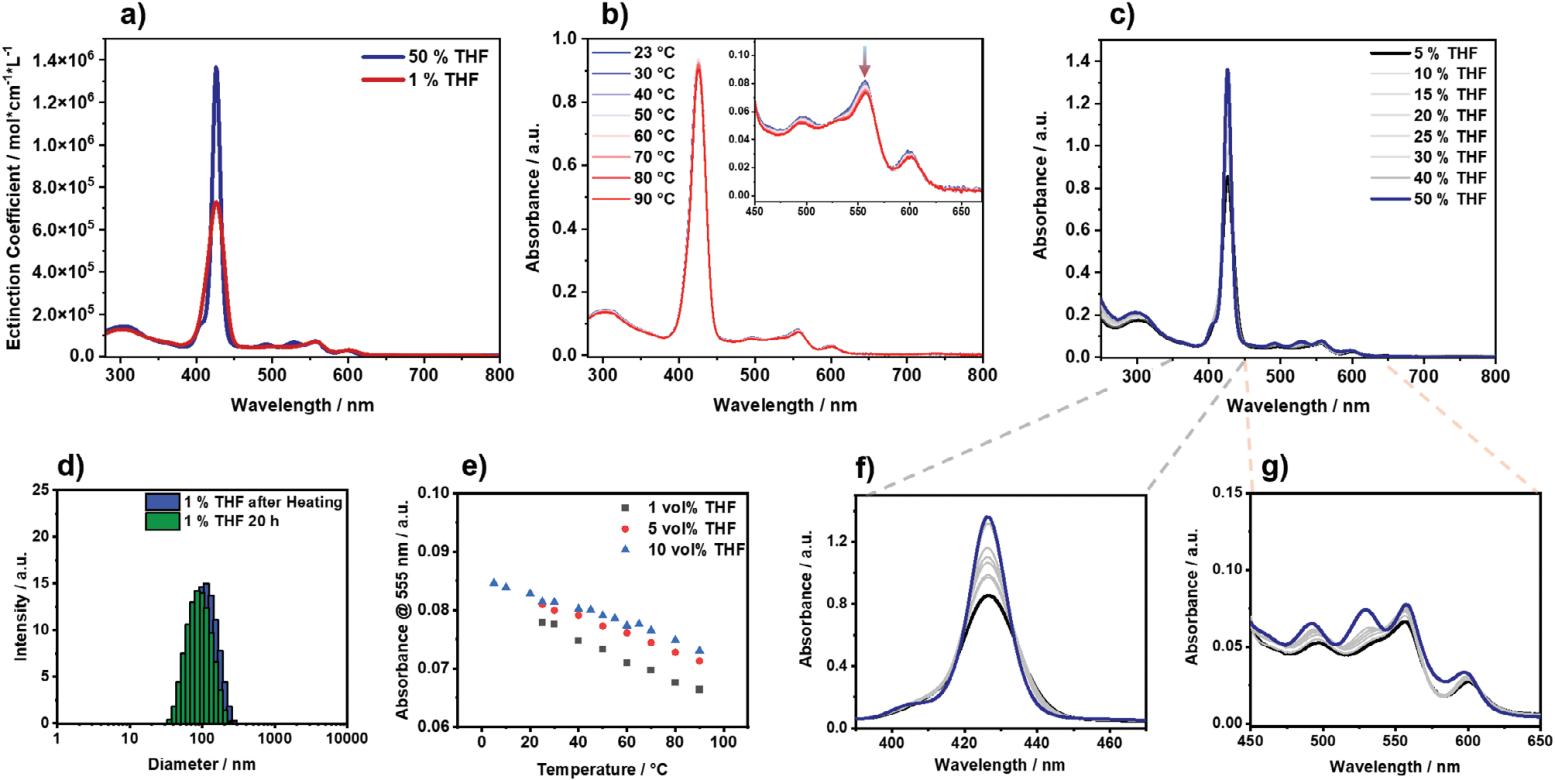
a) UV/Vis absorption spectra of **15** in 10 mm aqueous NaOH with different parts of THF as indicated; b) temperature dependent UV/Vis absorption spectra of **15** in 5 vol% THF in 10 mm NaOH (c = 1*10^−6^ m); c) UV/Vis absorption spectra of **15** in 10 mm aqueous NaOH with different parts of THF as indicated (f, g, are partial spectra of c)); For clarity, only the starting and end point are highlighted in color, for the whole data we refer to the Supporting Information, d) DLS size distribution intensity of **15** (c = 1*10^−6^ m) in 10 mm NaOH with the indicated amount of THF; e) tracing of the absorbance at 555 nm of **15** over different temperatures at different parts of THF (c = 1*10^−6^ m).

For all new conjugates, UV/Vis absorption spectra were recorded. Generally, the UV/Vis absorption spectra of all pentads are dominated by the porphyrin absorption owing to the 4:1 ratio of porphyrin to PBI. The spectra almost resemble the weighted sum of those of the individual chromophores, indicating only small ground‐state interactions. In detail, for pentad **8**, spectra were recorded in THF (Figure 2a). The characteristic features of the porphyrin as well as the PBI absorption appear, with the Soret‐band shown at 427 nm, and the Q‐bands at λQ(0,1) = 558 nm and λQ(0,0) = 598 nm. The features of the PBI can be observed at λPBI(0,1) = 492 nm and λPBI(0,0) = 528 nm. For the dendronized pentad **14**, the absorbance in THF (Figure 2a) displays similar features as **8** with only small differences in the absorption maxima at 426, 492, 529, 557, and 598 nm. Similarly, the oligo‐carboxylic acid dendronized pentad **15** (Figure 3a, blue) shows the same features at 426, 492, 529, 557, and 597 nm in a 1 to 1 (v/v) mixture of 10 mm aqueous NaOH and THF and exhibits an extinction coefficient in the range of **14**. This mixture was chosen as it provides a sufficiently high pH to effectively deprotonate the carboxylic acids, giving a homogeneous system. Consequently, this shows that the amphiphilic pentads are well solubilized at this concentration in the THF/NaOH(aq) 1 to 1 mixture, as they don't show any changes compared to **14**, which can be correlated with the presence of aggregated species.

Notably, all pentads exhibit only barely observable fluorescence emission. This is in agreement with other porphyrin PBI systems connected via the imide‐ or bay‐position by short linkers, which indicates efficient photoinduced energy‐ or charge‐transfer.^[^ ^14^ ^]^ However, porphyrin PBI triads linked to the imide position using tolane linkers still exhibit fluorescence. This indicates that despite the distance established by the tolane linker, the processes are highly efficient through the *ortho*‐position, which is in agreement with other literature known *ortho‐*donor‐PBI conjugates.^[^ ^33^, ^34^ ^]^

### 2.3 Aggregation Studies

As in both porphyrins^[^ ^35^, ^36^ ^]^ and PBIs,^[^ ^37^ ^]^ aggregation leads to characteristic changes in their absorption behavior,^[^ ^38^ ^]^ absorption spectroscopy was used to obtain first insights into the self‐assembly of the amphiphilic pentad. Therefore, the spectra of pentad **15** with the same concentration but varying amounts of THF in aqueous 10 mm NaOH solutions were recorded (Figure 3), as this forces the self‐assembly–individualization process. The excess base was used again to ensure uniform deprotonation of the pentads. In aqueous solutions with 5 vol% THF (Figure 3, black), the spectrum shows a broadened Soret band with a maximum of 427 nm. The PBI bands appear at 497 nm and as a shoulder at ≈538 nm. The porphyrin Q‐bands can be observed at 556 and 601 nm. Upon stepwise addition of more THF (up to 50 vol%; Figure 3, blue), the absorption of the Soret band gains in intensity and sharpness. The Q‐bands gain in intensity, and the Q(0,0) transition displays a slight blue shift to 597 nm, whereas the Q(0,1) transition does not show any significant shifts. Further, both observable PBI bands display a blue shift of ΔλPBI(0,1) = 5 nm and ΔλPBI(0,0) = 9 nm. Upon further addition of larger amounts of THF (up to 95 vol%), a decrease in absorbance and broadening of the Soret band is evident, indicating re‐aggregation and subsequent precipitation out of solution (Figure S54). These trends in the aggregation–individualization processes are analogous to similar bola‐amphiphilic systems but less drastic as the system is not fully water soluble.^[^ ^22^ ^]^ The initial shifts in the porphyrin bands can be explained by the coordination of THF to the metal center of the porphyrin, while the changes in the PBI absorption stem from PBI‐centered aggregation. Furthermore, the kinetic and thermodynamic stability of the aggregates was investigated. For this, time‐dependent absorption spectra were recorded of a 1*10^−6^ m solution of **15** in 10 mm NaOH/1 vol% THF (Figure S55). Here, no drastic changes were observed over the course of 20 h, indicating kinetic stability of the aggregates. To complement this, temperature‐dependent UV/Vis spectra of 1*10^−6^ m solutions of **15** were obtained (Figure 3e and Figures S57 and S58). While the spectra with 1 vol% of THF only reveal marginal changes, the spectra of the sample containing 5 vol% and 10 vol% THF indicate starting individualization. However, no full disaggregation was reached. Furthermore, cooling the 10 vol% THF sample to 5 °C did not yield fully aggregated species, indicating that the entire aggregation‐individualization process cannot be thermally induced at these concentrations. Tracing the absorbance of **15** at 555 nm over different temperatures with the respective THF contents (Figure 3e) shows a mostly linear correlation, corroborating the incomplete individualization. Lastly, the influence of the variation of the pH on the absorbance of the aggregates was investigated (Figure S56), where no significant changes between pH 12, pH 10, and pH 7.2 were found.

**Figure 4:**
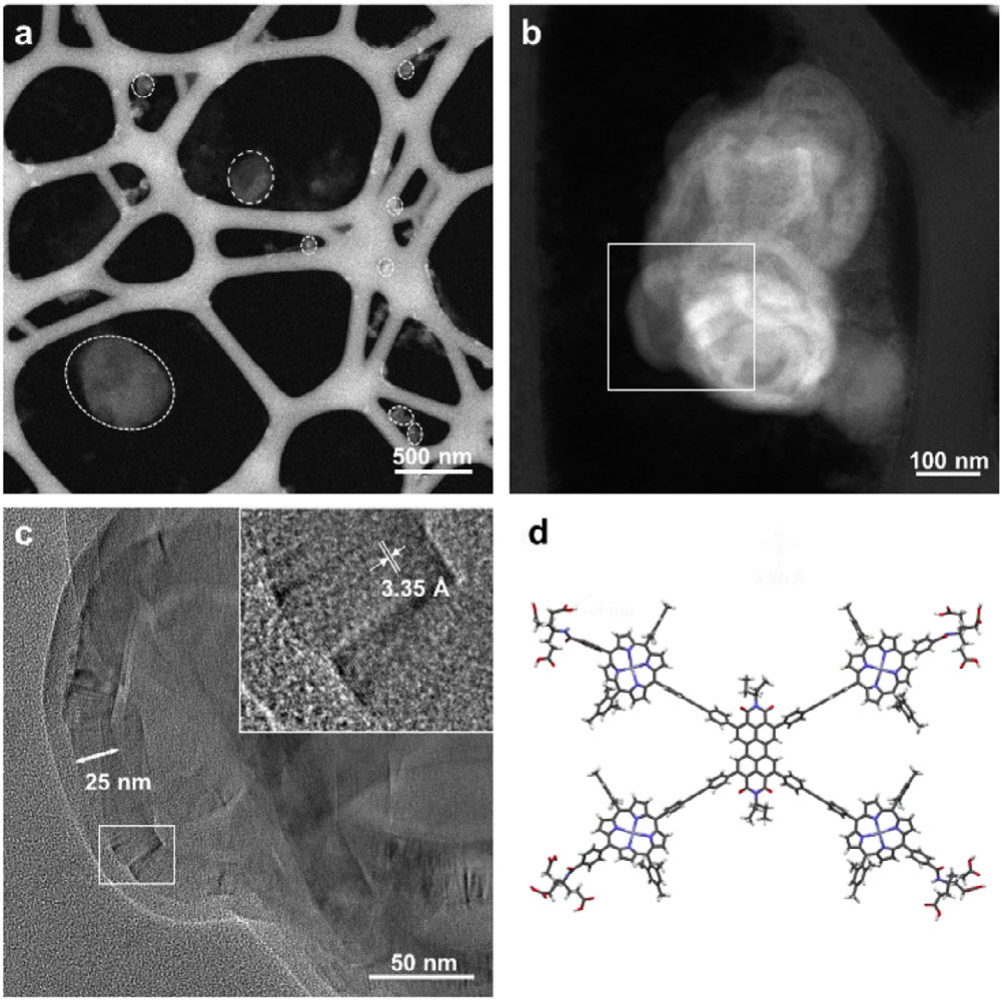
a,b) STEM‐HAADF (Z‐contrast) images and c) HRTEM image of the sodium salt of **15** (c = 0.25 mm, 5 vol% THF in H_2_O, without heating); d) geometry optimized structure (semi‐empirical‐PM3 level of theory). c) is from the identical region marked by the white box in b). The white box in (c) is magnified and shown as the inset.

To gain insights into the size and morphology of the aggregates, dynamic light scattering (DLS), scanning transmission electron microscopy (STEM) as well as transmission electron microscopy (TEM) experiments were conducted. The DLS measurement of samples of **15** containing 1 vol% THF in 10 mm NaOH (Figure 3d) reveals a broad size distribution intensity in the range of 50–300 nm. The distribution only marginally differs between aged and heated samples, indicating also the formation of rather stable structures regarding their morphology. We applied STEM high‐angle annual dark‐field (aka, Z‐contrast) imaging technique to reveal the morphology of the assemblies and aberration‐corrected high‐resolution TEM (HRTEM) imaging technique to investigate the lattice and molecular stacking in the assembles. Figure 4a–c are images of the sodium salt of **15** from a 5 vol% THF solution aged at room temperature for 16 hours. Over a large field, assembles of variable shape with sizes ranging from 30 to 600 nm can be seen on the lacy carbon support (Figure 4a), which is in agreement with the DLS results above. Despite having different morphology on the nanometer to tens of nanometer scale, a closer examination of the assembly seems to reveal that the underlying structures are coiled sheets and flakes (Figure 4b,c and Figure S75a–c,e,f), or even tubular and circular shapes (Figure S75d). The HRTEM lattice image clearly reveals that the sheets are lattices with a spacing 3.35 Å (Figure 4c), which is similar to the interlayer distance of graphite, i.e., the (002) lattice plane, when viewed edge‐on.

This most likely originates from the *π*–*π* stacking of the amphiphiles. The thickness of the *π*–*π* stacked sheets ranges from 20 to 50 nm. When preparing a sample by heating it for 2 hours at 60 °C in a sealed vial, flakes of varying morphology and only of a few nanometers thickness can be observed (Figures S75d,e and S76). As we noticed that the sample assembled at elevated temperatures showed higher stability under the electron bombardment, HRTEM images could be recorded with minimum damage, and the high spatial frequency lattice planes can be revealed in the Fourier transformed HRTEM. Interestingly, the underlying lattice spacings match well with some typical graphite spacings, which would infer a highly ordered and tightly packed structure of the amphiphiles (For a more detailed description, we refer to the Supporting Information).

To further assess the observed structures, amphiphile **15** was modelled using semi‐empirical methods (PM3) to access the amphiphiles geometry. The resulting structure (Figure 4d) underlines the assumed two‐dimensionality, with the chromophores being orientated co‐planar toward each other. Consequently, when taking the geometric structure of the amphiphile into account, a columnar stack can be hypothesized, which continuously assembles into the observed sheets and flakes. The results obtained from the HRTEM imaging suggest a tightly packed, highly regular, co‐facial alignment. Moreover, the perpendicular orientation of the amphiphile face toward the length of the sheets shows that next to the hydrophobic *π*–*π* interactions, also interactions between the carboxylates, for example ion mediated interactions, dictate the assembly of these amphiphiles. However, such an assembly structure is, in our opinion, unusual and needs further corroboration, as the interactions of the carboxylates would overcome the steric hindrance of the mesityl‐porphyrins to allow for the slipped face‐to‐face stacking. Therefore, further microscopical investigations accompanied by more sophisticated modelling are needed to verify or dismiss any exact structural propositions, which we are planning to pursue in the future.

## 3 Conclusions

In conclusion, we accomplished the synthesis of the first *ortho‐*linked porphyrin‐PBI conjugate **8** and the corresponding discotic amphiphilic pentad **15**. This was achieved by a fourfold Suzuki cross‐coupling of *tetra*‐*ortho*‐functionalized PBIs with complementary zinc‐porphyrin precursors. For the synthesis of the amphiphile, oligo‐carboxylic acid G1, Newkome dendrons were attached to the porphyrins prior to coupling. These new hybrids were unambiguously characterized by NMR spectroscopy and HRMS. The amphiphilic derivative **15** displays good solubility in acidified THF, as well as mixtures of basic water and THF. All pentads show drastically quenched fluorescence emission, indicating efficient charge‐ or energy‐transfer between the porphyrin and the PBI moieties. Investigation of the self‐assembly behavior by UV/Vis spectroscopy reveals the formation of aggregates centered around the PBI core. These aggregates are stable over time and only partially individualize at higher temperatures. Further analysis of the morphology of the system via DLS and TEM/STEM unravels that **15** assembles into coiled stacks of graphitic‐like flakes of ≈50–600 nm. Notably, the assembly is not only guided by the hydrophobic *π*–*π* interactions, but also by the interactions of the carboxylates. This new family of porphyrin‐PBI conjugates further expands the scope of integrated charge‐ and energy‐transfer amphiphiles, contrasting the structure and morphology of prior published porphyrin‐PBI amphiphiles. Furthermore, these *ortho*‐PBIs fill a gap in the library of porphyrin‐PBI systems. In the future, we aim to further investigate in great detail their photophysical properties as well as their applicability as light harvesters in photo‐redox reactions.

## 4 Experimental Section

The detailed synthetic procedures, characterization protocols, and data used for characterization are provided in the Supporting Information.

## Supporting Information

The authors have cited additional references within the Supporting Information.^[^ ^39^, ^40^ ^]^

## Conflict of Interest

The authors declare no conflict of interest.

## Supporting information



Supporting Information

## Data Availability

The data that support the findings of this study are available in the supplementary material of this article.
