# Supplementary material for: Synthesis and Self‐Assembly of a Discotic Oligo‐Carboxylate Tetra‐Porphyrin‐Perylenebisimide Amphiphile
Source: Chemistry. 2025 Jul 22;31(45):e02093. doi: 10.1002/chem.202502093 (PMC12351443; doi:10.1002/chem.202502093)
Supplement: Supplementary file 1 — Supporting Information [file CHEM-31-e02093-s001.pdf]

Table of Content

1. **Synthesis** 2
   1.1. **Materials and Methods** 2
   1.2. **Synthetic Procedures** 4
2. **NMR Spectra** 20
3. **UV/Vis / Fluorescence Spectra** 45
4. **UV/Vis Aggregation Studies** 50
5. **Dynamic Light Scattering** 54
6. **Mass Spectra** 55
7. **Microscopical Imaging** 64
8. **Theoretical Investigations** 66
9. **References** 67

1. Synthesis

1.1. Materials and Methods

Chemicals were purchased from Sigma-Aldrich, TCI and Fischer, and used without any further purification. Solvents of technical grade were distilled prior to usage. Solvents of HPLC grade were used as received. Solvents were degassed by three cycles of ultrasonication under a vacuum followed by subsequent refilling with a protective gas (Ar or N_2). Thin layer chromatography (TLC) was performed on Merck silica gel 60 F524, detected by UV light (254 nm, 366 nm). Silica gel plug filtration and column chromatography were performed on Macherey-Nagel silica gel 60 M (deactivated, 230−400 mesh, 0.04−0.063 mm). Gel permeation size exclusion chromatography was performed on BioBeads SX1 from BioRad in $CHCl_3$ and toluene.

Reactions including heating with microwave irradiation were performed in an Anton Paar Monowave 450 using Anton Paar G30 microwave vials (30 bar max. pressure). The reaction mixtures were heated as fast as possible to the desired temperature with a maximum power of 850 W and subsequently cooled to 35 °C using pressurized air prior to the described workup procedure.

NMR spectra were recorded on a Bruker Avance 400 (^1H: 400 MHz, ^{13}C: 101 MHz), a Bruker Avance 500 (^1H: 500 MHz, ^{13}C: 126 MHz), or a Bruker Avance Neo Cryo-Probe DCH (^1H: 600 MHz, ^{13}C: 150 MHz). Deuterated solvents were purchased from Sigma-Aldrich, ARMAR Isotopes, and Deutero and used as received. Chemical shifts are given in ppm at room temperature and are referenced to residual protic impurities in the solvents (^1H: $CDCl_3$: 7.24 ppm, CD_2Cl_2: 5.34 ppm, THF-d_8: 3.58 ppm, D_2O: 4.79 ppm) or the deuterated solvent itself ($^{13}C\{^1H\}$: $CDCl_3$: 77.16 ppm, CD_2Cl_2: 53.4 ppm, THF-d_8: 66.57 ppm). The resonance multiplicities are indicated as “s” (singlet), “brs” (broad singlet), “d” (doublet), “t” (triplet), “q” (quartet), and “m” (multiplet). Mass spectrometry was carried out with a Shimadzu AXIMA Confidence (MALDI-TOF, matrix: 2,5-dihydroxybenzoic acid DHB, trans-2-[3-(4-tert-butylphenyl)-2-methyl-2-propenyliden]-malononitrile, (DCTB) or without matrix (OM). High-resolution mass spectrometry (HRMS) was recorded on a LDI/MALDI-ToF Bruker Ultraflex Extreme machine or on a micrOTOF II (ESI- / APPI–TOF) focus mass spectrometer (Bruker).

UV/vis spectroscopy was carried out on a Varian Cary 5000 UV−vis−NIR spectrometer. The spectra were recorded at room temperature in deoxygenated solutions in quartz cuvettes (edge length = 1 cm) under ambient conditions. Fluorescence spectra were obtained from a Shimadzu RF-5301 PC and a NanoLog spectrofluorometer. Dynamic Light Scattering (DLS) was performed on a Zetasizer Nano Series ZEN3600 (Malvern Instruments) with a 633 nm

He-Ne laser. IR was measured on an PerkinElmer Frontier FT-IR spectrometer equipped with a PerkinElmer “Universal ATR Sampling Accessory” unit

1.2. Synthetic Procedures

The synthetic procedures for **G1-NH$_2$**[1] and **Mesityl dipyrromethan**[2] were published by us before.

4-((4-bromophenyl)ethynyl)benzaldehyde

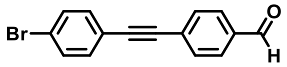

Under dinitrogen atmosphere, 1-bromo-4-iodobenzene (3.90 g, 13.8 mmol, 1.2 eq.), $Pd(PPh_3)_2Cl_2$ (204 mg, 0.29 mmol, 0.025 eq.) and CuI (87.6 mg, 0.46 mmol, 0.04 eq.) were dissolved in Et_3N (40 mL). 4-Ethynylbenzaldehyde (1.50 g, 11.5 mmol, 1.0 eq.) was dissolved in Et_3N (15 mL) and THF (5.0 mL), and the solution was added slowly over 10 min to the reaction mixture. The reaction mixture was stirred at rt. for 1 h 20 min. The volatiles were removed under reduced pressure and the residue was dissolved in DCM (50 mL). The crude product was purified via a silica gel plug (DCM → EtOAc). The volatiles were removed under reduced pressure. The product was dried *in vacuo* and compound **Bromo-Tolane** (3.08 g, 10.8 mmol, 94%) was yielded as a beige-golden powder.

^{1}H NMR (400 MHz, $CDCl_3$): δ (ppm) = δ = 10.03 (s, 1H), 7.87 (d, J = 8.5 Hz, 2H) 7.67 (d, J = 8.2 Hz, 2H), 7.52 (d, J = 8.7 Hz, 2H), 7.41 (d, J = 8.7 Hz, 2H)

^{13}C NMR (101 MHz, $CDCl_3$): δ (ppm) = 191.49, 135.74, 133.32, 132.25, 131.94,129.75, 129.34,123.49, 121.60, 92.39, 89.71

MS (APPI) m/z for $C_{15}H_{10}BrO$ $[M+H]^+$ calculated: 284.9910; found: 284.9936.

A_3B-3,5-di(*tert*butyl) phenyl-bromo-tolane-porphyrin (1)

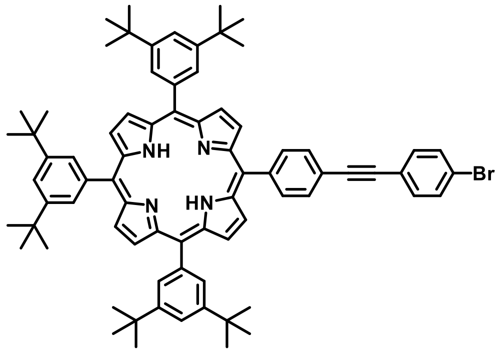

Adapting a literature procedure,[3] two microwave vials were each loaded with compound **3** (129 mg, 0.500 mmol, 1.0 eq.), 3,5-di-*tert*-butylbenzaldehyde (328 mg, 1.50 mmol, 3.0 eq.), pyrrole (140 µL, 136 mg, 2.02 mmol, 4.0 eq.) and DCM (19 mL). A solution of I_2 (25.4 mg, 0.1 mmol, 0.2 eq.) in DCM (1.0 mL) was added. The mixtures were stirred in a microwave reactor (5 min at 40 °C). In a separate flask, *para*-chloranil (369 mg, 1.5 mmol, 3.0 eq.) was added to DCM (20 mL) and heated to reflux. After that, the mixtures from both microwave tubes were poured into the prepared *para*-chloranil solution, and the resulting solution was stirred under reflux for 20 min. After cooling down to rt., the mixture was purified via a silica gel plug (DCM + 1% Et_3N) followed by column chromatography (*iso*-hexane/DCM 3:1 to 2:1, + 1% Et_3N). The volatiles were removed under reduced pressure and compound **4** (66.7 mg, 59.0 µmol, 5.9%) was yielded as a dark violet powder.

^{1}H NMR (400 MHz, $CDCl_3$): δ (ppm) = 8.91-8.83 (m, 8H), 8.23 (d, J = 8.3 Hz, 2H), 8.08-8.07 (m, 6H), 7.91 (d, J = 8.3 Hz), 7.80-7.78 (m, 3H), 7.59-7.53 (m, 4H), 1.53-1.52 (m, 54H), -2.70 (s, 2H)

^{13}C NMR (101 MHz, $CDCl_3$): δ (ppm) = 148.89, 141.36, 134.70, 133.33, 131.91, 129.93, 129.83, 122.82, 122.40, 121.68, 121.18, 118.66, 90.78, 35.20, 31.89.

HRMS (MALDI-TOF, dctb) m/z for $C_{76}H_{81}BrN_4$: $[M]^+$ calculated: 1128.5639; found: 1128.5612.

UV/Vis (THF): $\varepsilon(\lambda_{max})$ = 460 000 (420), 21 000 (516), 13 000 (551), 7 300 (594), 6 400 (649) $L{*}mol^{-1}{*}cm^{-1}$ (nm).

Fluorescence (THF, RT, exc. 423 nm) λ_{max} [nm] (rel. Int. [%]): 652 (100), 717 (21).

Zinc-A$_3$B-3,5-di(*tert*butyl) phenyl-bromo-tolane-porphyrinato zinc (II) (2)

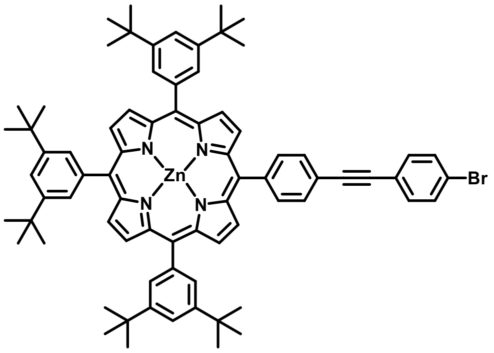

$Zn(OAc)_2$ (32.4 mg, 177 µmol, 4.0 eq.) was dissolved in methanol (5.0 mL) and added to a solution of compound **4** (50.0 mg, 44.2 µmol, 1.0 eq.) in chloroform (12.5 mL). The mixture was stirred under reflux for 2.5 h. After cooling down to rt., the crude product was purified via a silica gel plug (DCM + 0.1% Et_3N). The volatiles were removed under reduced pressure, yielding compound **5** (53.1 mg, 44.5 µmol, quant.) as a pink-violet powder.

^{1}H NMR (400 MHz, $CDCl_3$): δ (ppm) = 9.02-8.94 (m, 8H), 8.24 (d, J = 8.3 Hz), 8.09-8.08 (m, 6H), 7.91 (d, J = 8.3 Hz, 2H), 7.80-7.78 (m, 3H), 7.59-7.53 (m, 4H), 1.54-1.52 (m, 54H)

^{13}C NMR (101 MHz, $CDCl_3$): δ (ppm) = 150.71, 150.61, 150.57, 149.89, 148.74, 143.65, 141.92, 134.57, 133.32, 132.64, 132.50, 132.39, 131.90, 131.55, 129.96, 129.82, 129.75, 122.96, 122.75, 122.52, 122.21, 121.00, 119.80, 90.87, 89.39, 35.21, 31.92.

HRMS (APPI) m/z for $C_{76}H_{79}N_4Zn$: $[M]^+$ calculated: 1190.4774; found: 1190.4778.

UV/Vis ($CHCl_3$): quantitatively (λ_{max}) = 423, 549, 587 nm.

Fluorescence ($CHCl_3$, exc. 423 nm) λ_{max} [nm] (rel. Int. [%]): 595 (94), 645 (100).

A$_3$B-3,5-di(*tert*butyl) phenyl-bromo tolane porphyrinato zinc (II) (3)

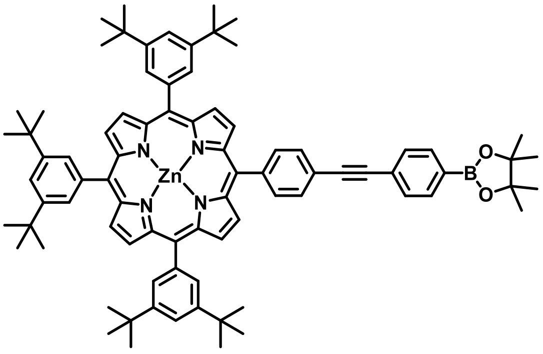

To a 10 mL microwave vial, **2** (20 mg, 16.8 µmol), B_2Pin_2 (6.4 mg, 25.1 µmol, 1.5 eq.), KOAc (4.9 mg, 50.4 µmol, 3 eq.) and Pd(dppf)Cl_2 (1.2 mg, 1.68 µmol) were added, and the vial was evacuated and refilled with nitrogen three times. Then, degassed anhydrous dioxane (0.5 mL) was added and the reaction mixture was heated to 90 °C for 5 d. Following, the reaction mixture was allowed to cool to room temperature and the reaction mixture was passed through a short silica plug (eluent CH_2Cl_2). Purification of the crude product by column chromatography (SiO_2, CH_2Cl_2/Hex 1/1→ CH_2Cl_2) yielded the desired borylated porphyrin as purple powder (17.1 mg, 13.8 µmol, 82 %).

^{1}H NMR (400 MHz, $CDCl_3$) δ = 9.04 – 9.03 (m, 4H), 8.98 – 8.95 (m, 4H), 8.24 (d, *J*=8.2, 2H), 8.12 – 8.07 (m, 6H), 7.94 (d, *J*=8.2, 2H), 7.88 (d, *J*=8.2, 2H), 7.82 – 7.78 (m, 3H), 7.69 (d, *J*=8.1, 2H), 1.53 (s, 36H), 1.53 (s, 18H), 1.40 (s, 12H).

^{13}C NMR (101 MHz, $CDCl_3$) δ = 150.53, 150.44, 150.42, 149.79, 148.58, 148.55, 143.36, 141.82, 141.80, 134.74, 134.41, 132.48, 132.32, 132.22, 131.46, 130.94, 129.89, 129.68, 129.62, 126.09, 122.76, 122.57, 122.31, 120.84, 119.75, 90.92, 90.52, 84.04, 67.55, 35.07, 31.78, 29.73, 24.95.

HRMS (MALDI-TOF, dctb) m/z for $C_{82}H_{91}BN_4O_2Zn$: $[M]^+$ calculated: 1238.6521; found: 1238.6534.

UV/Vis (THF): ε(λ_{max}) = 660 000 (426), 24 000 (557), 12 000 (597) L*mol^{-1}*cm^{-1} (nm).

Fluorescence (THF, RT, exc. 423 nm) λ_{max} [nm] (rel. Int. [%]): 603 (100), 655 (70).

N,N′-di(ethylpropyl)-2,5,8,11-tetra(4′,4′,5′,5′-tetramethyl-1′,3′,2′-dioxaborolan-2′-yl)perylene-3,4:9,10-bis(dicarboximide) (5)

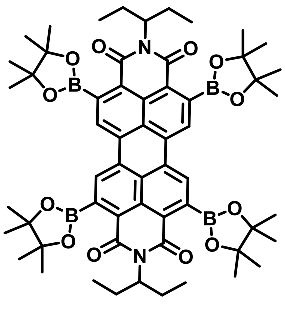

5

Tetra-borylated PBI **5** was prepared according to a literature procedure,[4] and the spectroscopic data is in accordance with the ones reported in 61 % yield.

^{1}H NMR (400 MHz, $CDCl_3$) δ (ppm) = 8.52 (s, 4H), 4.98 – 4.85 (m, 2H), 2.25 – 2.10 (m, 4H), 2.01 – 1.87 (m, 4H), 1.54 (s, 48H), 0.92 (t, *J*=7.5, 12H).

^{13}C NMR (101 MHz, $CDCl_3$) δ (ppm) = 165.92, 138.56, 133.31, 128.22, 127.11, 126.87, 125.94, 84.61, 58.15, 25.17, 11.74.

MS (APPI) m/z for $C_{58}H_{75}B_4N_2O_{12}$ $[M+H]^+$ calculated: 1035.5687; found: 1035.5732

N,N′-di(ethylpropyl)-2,5,8,11-tetrabromoperylene-3,4:9,10-bis(dicarboximide) (6)

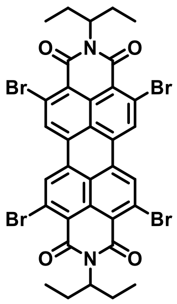

6

Tetra-bromo PBI **6** was prepared according to a literature procedure,[5] and the spectroscopic data is in accordance with the ones reported in 82 % yield.

^{1}H NMR (400 MHz, $CDCl_3$) δ (ppm) = 8.77 – 8.70 (m, 4H), 5.11 – 5.01 (m, 2H), 2.30 – 2.16 (m, 4H), 2.07 – 1.91 (m, 4H), 0.94 (t, *J*=7.5, 12H).

^{13}C NMR (101 MHz, $CDCl_3$) δ (ppm) = 161.26, 132.82, 132.27, 131.64, 129.42, 124.68, 121.57, 59.09, 25.06, 11.58.

HRMS (APPI) m/z for $C_{34}H_{27}Br_4N_2O_4$ $[M+H]^+$ calculated: 842.8699; found: 842.8695

N,N′-di(ethylpropyl)-2,5,8,11-tetraiodoperylene-3,4:9,10-bis(dicarboximide) (7)

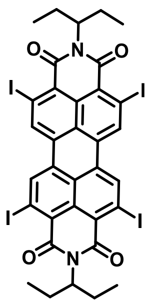

7

Tetra-iodo PBI **7** was prepared according to a literature procedure,[6] and the spectroscopic data is in accordance with the ones reported in 16 % yield.

^{1}H NMR (400 MHz, $CDCl_3$) δ = 9.10 (s, 4H), 5.13 – 5.01 (m, 2H), 2.33 – 2.12 (m, 4H), 2.10 – 1.90 (m, 4H), 0.94 (t, *J*=7.6, 12H).

^{13}C NMR (101 MHz, $CDCl_3$) δ = 161.07, 138.72, 132.00, 131.53, 125.90, 123.57, 101.39, 59.35, 25.01, 11.55.

MS (APPI) m/z for $C_{34}H_{27}I_4N_2O_4$ $[M+H]^+$ calculated: 1034.8144; found: 1034.8156

Tetra-(3,5-di(*tert*butyl) phenyl-bromo-tolane-porphyrin)-perylenebisimide (8)

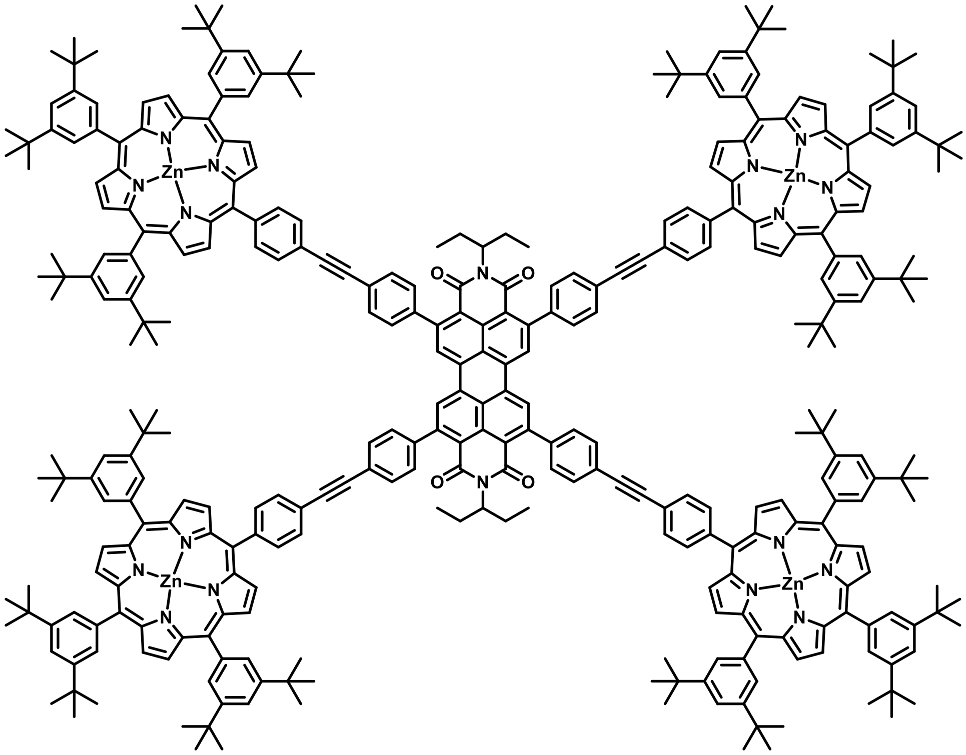

To a 10 mL microwave tube PBI **6** (4.1 mg, 4.83 µmol, 1 eq), porphyrin **3** (30.0 mg, 24.2 µmol, 5.0 eq.), Cs_2CO_3 (9.4 mg, 29 µmol, 6.0 eq.) and $Pd(PPh_3)_4$ (2.2 mg, 1.9 µmol, 0.4 eq.) were added and the vial was evacuated and refilled with nitrogen three times. Then, a mixture of degassed toluene (2.0 mL) and DMF (1.0 mL) was added, and the mixture was stirred for 16 h at 80 °C. After cooling to rt., the mixture was filtered through a short silica gel plug (CH_2Cl_2/Hexanes 1:1) and the solvent was removed under reduced pressure. Then, the crude product was purified by SEC (BioBeads SX1, $CHCl_3$ Ø 2.5 cm, ↑ 60 cm) and subsequent column chromatography (SiO_2, CH_2Cl_2/Hexanes 1:2), yielding the desired product **8** as a red solid (5.0 mg, 1.0 µmol, 21 %)

^{1}H NMR (600 MHz, $CDCl_3$): *δ* (ppm) = 9.02-8.99 (m, 32H), 8.58 (s, 4H), 8.27 (d, *J* = 7.9 Hz, 8H), 8.10-8.08 (m, 24H), 7.99 (d, *J* = 7.9 Hz, 8H), 7.90 (d, *J* = 7.9 Hz, 8H), 7.79-7.76 (m, 12H), 7.61 (d, *J* = 7.9 Hz, 8H), 4.97-4.93 (m, 2H), 2.21-2.16 (m, 8H), 1.88-1.82 (m, 12H), 1.54-1.50 (m, 216H).

^{13}C NMR ^{13}C NMR (151 MHz, THF-*d₈*) δ = 150.29, 150.17, 150.16, 149.58, 148.17, 147.34, 147.32, 144.14, 142.91, 142.80, 142.79, 134.46, 132.87, 131.64, 131.47, 131.41, 131.30, 131.21, 130.76, 129.54, 129.44, 129.41, 128.63, 127.58, 125.58, 122.56, 122.37, 121.70, 121.60, 120.42, 119.07, 113.56, 90.09, 89.96, 57.41, 46.34, 34.68, 34.67, 31.10, 31.08, 10.76.

HRMS (MALDI-TOF, dctb) m/z for $C_{338}H_{344}N_{18}O_4Zn_4$: $[M]^+$ calculated: 4974.4428; found: 4974.4398.

UV/Vis (THF): $\varepsilon(\lambda_{max})$ = 2070000 (427), 57 000 (492), 72 000 (528), 83 000 (558), 41 000 (599) $L{*}mol^{-1}{*}cm^{-1}$ (nm).

AB$_2$C-methylbenzoate-mesityl-bromo-tolane-porphyrin (9)

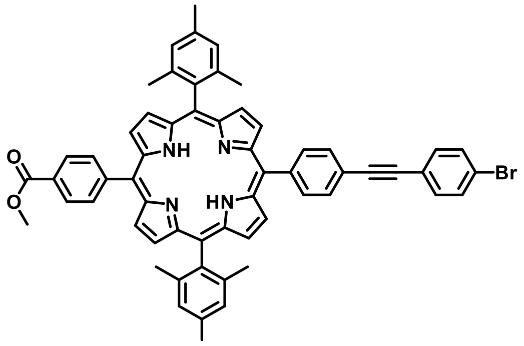

9

Under an argon atmosphere, mesityldipyrromethan (529 mg, 2.00 mmol, 2.0 eq.), 4-((4-bromophenyl)ethynyl)benzaldehyde (286 mg, 1.00 mmol, 1.0 eq.) and methyl 4-formylbenzoate (165 mg, 1.01 mmol, 1.0 eq.) were dissolved in $CHCl_3$ (300 mL) and EtOH (5.00 mL), and the solution was deoxygenated with argon for 15 min. Under exclusion of light, $BF_3{\cdot}OEt_2$ (114 µL, 128 mg, 0.90 mmol, 0.9 eq.) was added, and the mixture was stirred at rt. After 2 h, DDQ (639 mg, 2.81 mmol, 2.8 eq.) was added, and the reaction mixture was stirred again at rt. for 1 h 15 min. Then, the solvent was removed under reduced pressure. The crude product was purified via a silica gel plug (DCM + 0.1% Et_3N) followed by silica gel flash column chromatography (*iso*-hexane/DCM 5:1 to 1:1). The product was further purified via a column chromatography (iso-hexane/DCM 2:1 to 1:1, + 0.1% Et_3N). The volatiles were removed under reduced pressure and compound **9** (170 mg, 182 µmol, 18%) was obtained as a dark violet powder.

^{1}H NMR (400 MHz, $CDCl_3$): δ (ppm) = 8.81 – 8.71 (m, 8H), 8.44 – 8.42 (m, 2H), 8.32 – 8.30 (m, 2H), 8.24 – 8.21 (m, 2H), 7.93 – 7.90 (m, 2H), 7.59 – 7.53 (m, 4H), 7.29 (s, 4H), 4.11 (s, 3H), 2.63 (s, 6H), 1.84 (s, 12H), -2.63 (s, 2H) ppm.

^{13}C NMR (101 MHz, $CDCl_3$): δ (ppm) = 167.50, 146.99, 142.44, 139.50, 138.40, 138.01, 134.72, 134.68, 133.31, 131.92, 130.12, 129.70, 128.06, 127.96, 122.88, 122.55, 122.40, 118.88, 118.85, 118.18, 90.66, 89.65, 52.57, 21.78, 21.62.

HRMS (MALDI-TOF, dctb) m/z for $C_{60}H_{47}BrN_4O_2$: [M]+ calculated: 934.2877; found: 934.2859.

UV/Vis (THF): ε(λ_{max}) = 472 000 (419), 21 000 (514), 10 000 (548), 6 000 (591), 5 000 (648) L*mol^{-1}*cm^{-1} (nm).

Fluorescence (THF, exc. 420 nm) λmax [nm] (rel. Int. [%]): 651 (100), 717 (24)

AB$_2$C-methylbenzoate-mesityl-bromo-tolane-porphyrinato zinc(II) (10)

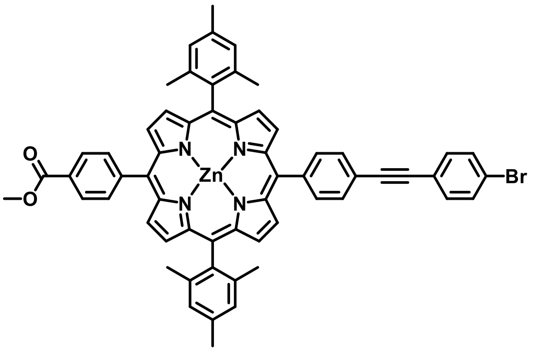

10

Zn(OAc)$_2$·(48.9 mg, 223 µmol, 4.1 eq.) was dissolved in MeOH (6.0 mL), and was added to a solution of free-base porphyrin **9** (50.7 mg, 54.2 µmol, 1.0 eq.) in $CHCl_3$ (15 mL). The reaction mixture was stirred under reflux for 1:45 h. After cooling down to room temperature, the crude product was filtered thorugh a silica gel plug (DCM + 0.1% Et_3N). The volatiles were removed under reduced pressure, and the metalated porphyrin **10** was (56.6 mg, 56.6 µmol, 99%) was obtained as a pink-violet powder.

^{1}H NMR (400 MHz, $CDCl_3$): δ (ppm) = 8.90 – 8.79 (m, 8H), 8.43 – 8.41 (m, 2H), 8.33 – 8.31 (m, 2H), 8.25 – 8.23 (m, 2H), 7.92 – 7.90 (m, 2H), 7.58 – 7.53 (m, 4H), 7.29 (s, 4H), 4.10 (s, 3H), 2.63 (s, 6H), 1.83 (s, 12H).

^{13}C NMR (101 MHz, $CDCl_3$): δ = 167.55, 150.23, 150.18, 149.94, 149.68, 147.86, 143.30, 139.35, 139.00, 137.74, 134.65, 134.59, 133.30, 132.34, 132.14, 131.90, 131.26, 131.20, 129.97, 129.41, 127.89, 127.85, 122.81, 122.46, 122.25, 119.81, 119.74, 119.11, 90.78, 89.49, 52.51, 21.79, 21.62.

HRMS (MALDI-TOF, dctb) m/z for $C_{60}H_{45}BrN_4O_2Zn$: [M]+ calculated: 996.2012; found: 996.1986.

UV/Vis (THF): ε(λ_{max}) = 596 000 (426), 23 000 (557), 8 000 (599) L*mol^{-1}*cm^{-1} (nm).

Fluorescence (THF, exc. 426 nm) λmax [nm] (rel. Int. [%]): 606 (100), 655 (68).

AB$_2$C-benzoic acid-mesityl-bromo-tolane-porphyrinato zinc(II) (11)

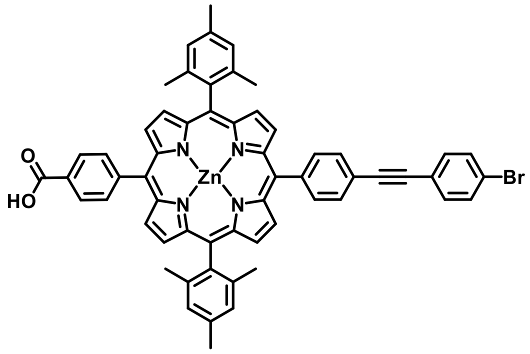

11

LiOH (81.5 mg, 3.40 mmol, 60 eq.) was dissolved in H_2O (3.40 mL) and was added to a solution of compound **10** (56.6 mg, 56.6 µmol, 1.0 eq.) in THF (13 mL). The mixture was stirred at room temperature for 28 h. The volatiles were removed under reduced pressure, and the residue was dissolved in DCM (150 mL). The organics were washed with H_2O (3 x 100 mL) and the volatiles of the organic phase were removed under reduced pressure. The crude product was purified twice via a short column chromatography (DCM → EtOAc → EtOAc + 1% acetic acid). The volatiles were removed under reduced pressure, and compound **11** (55.4 mg, 56.2 µmol, 99%) was obtained as a pink-violet powder.

^{1}H NMR (400 MHz, $CDCl_3$): δ (ppm) = 8.90 – 8.80 (m, 8H), 8.49 – 8.47 (m, 2H), 8.37 – 8.35 (m, 2H), 8.25 – 8.23 (m, 2H), 7.93 – 7.91 (m, 2H), 7.59 – 7.53 (m, 4H), 7.29 (s, 4H), 2.64 (s, 6H), 1.83 (s, 12H).

^{13}C NMR (101 MHz, $CDCl_3$): δ = 150.23, 150.16, 149.93, 149.64, 143.34, 139.35, 139.03, 137.71, 134.66, 133.30, 132.34, 131.90, 131.27, 131.18, 129.96, 128.42, 127.85, 122.81, 122.47, 122.23, 119.79, 119.74, 90.79, 89.48, 21.80, 21.61.

HRMS (MALDI-TOF, dctb) m/z for $C_{59}H_{43}BrN_4O_2Zn$: [M]+ calculated: 982.1855; found: 982.1821.

UV/Vis (THF): ε (λ_{max}) = 326 000 (426), 13 000 (557), 4 000 (599) L*mol^{-1}*cm^{-1} (nm).

Fluorescence (THF, exc. 426 nm) λ_{max} [nm] (rel. Int. [%]): 606 (100), 655 (70).

AB$_2$C-Newkome-G1-benzamide-mesityl-bromo-tolane-porphyrinato zinc(II) (12)

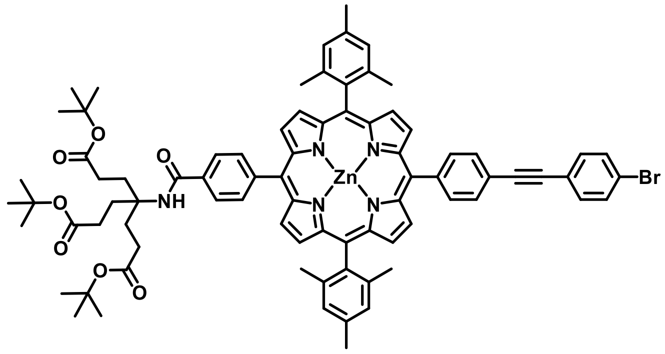

12

Carboxylic acid porphyrin **11** (30.3 mg, 30.8 µmol, 1.0 eq.), **G1-NH$_2$** (20.1 mg, 48.4 µmol, 1.6 eq.) and HOBt·H_2O (7.13 mg, 46.6 µmol, 1.5 eq.) were dissolved in DMF (10.0 mL), and the mixture was cooled down to 0 °C. DCC (8.54 mg, 41.4 µmol, 1.3 eq.) was dissolved in DMF (5.00 mL) and added to the mixture. The reaction mixture was allowed to warm up to rt., and was stirred for 7 d. The mixture was diluted by DCM (100 mL) and washed with water (3 x 80 mL). The aqueous phase was extracted with DCM (3 x 50 mL) and the combined organics were dried over $MgSO_4$. The volatiles were removed under reduced pressure and the residue was purified via a silica gel plug (DCM), followed by column chromatography (EtOAc/DCM 1:35 to 1:25). After drying *in vacuo*, compound **12** (16.3 mg, 11.8 µmol, 38%) was obtained as a pink powder.

^{1}H NMR (400 MHz, $CDCl_3$): δ = 8.90 – 8.79 (m, 8H), 8.30 – 8.28 (m, 2H), 8.25 – 8.23 (m, 2H), 8.16 – 8.14 (m, 2H), 7.92 – 7.90 (m, 2H), 7.58 – 7.52 (m, 4H), 7.29 (s, 4H), 2.64 (s, 6H), 2.46 (t, J = 7.9 Hz, 6H), 2.27 (t, J = 7.7 Hz, 6H), 1.84 (s, 12H), 1.49 (s, 27H).

^{13}C NMR (101 MHz, $CDCl_3$): δ = 173.41, 167.13, 150.19, 149.88, 143.33, 139.38, 139.05, 137.72, 134.65, 134.59, 134.33, 133.30, 132.28, 131.90, 131.17, 129.97, 127.85, 125.36, 122.81, 122.47, 122.23, 119.73, 119.35, 90.79, 89.48, 81.05, 58.24, 30.52, 30.24, 28.29, 21.80, 21.63.

HRMS (MALDI-TOF, dhb) m/z for $C_{81}H_{82}BrN_5O_7Zn$: [M]+ calculated: 1379.4684; found: 1379.4646.

UV/Vis (THF): ε (λ_{max}) = 630 000 (426), 25 000 (557), 9 000 (598) L*mol^{-1}*cm^{-1} (nm).

Fluorescence (THF, exc. 426 nm) λ_{max} [nm] (rel. Int. [%]): 605 (100), 655 (72)

AB$_2$C-Newkome-G1-benzamide-mesityl-pinacolatoboron-tolane-porphyrinato zinc(II) (13)

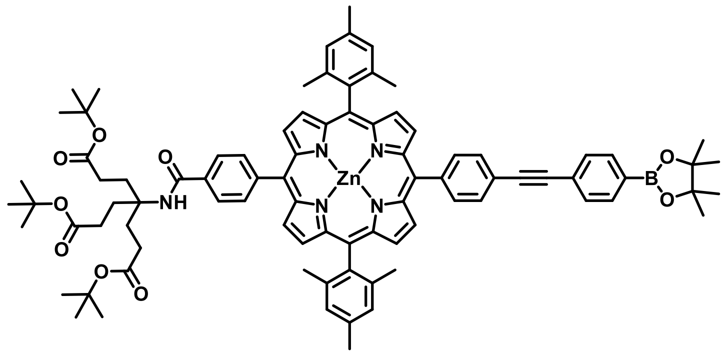

13

To a 10 mL microwave vial G1-bromo-Porphyrin **12** (50.0 mg, 36.2 µmol), B_2Pin_2 (13.8 mg, 54.2 µmol, 1.5 eq.), KOAc (10.6 mg, 108 µmol, 3 eq.) and Pd(dppf)Cl_2 (2.6 mg, 3.6 µmol, 0.1 eq.) were added, the vial was sealed, and evacuated and refilled with nitrogen three times. Then, degassed dry 1,4-Dioxane (2 mL) was added, and the reaction mixture was heated to 90 °C for 5 days, until TLC indicated full consumption of the starting material. Following, the mixture was allowed to cool to room temperature, and was then filtered through a pad of celite. Purification by a short silica plug filtration (EtOAc:*iso*-Hexane 1:3) followed by trituration with MeOH gave the desired borylated G1-porphyrin **13** as a purple powder (45 mg, 31.5 µmol, 87 %)

^{1}H NMR (500 MHz, THF-d_8) δ (ppm) = 8.84 – 8.79 (m, 4H), 8.69 (d, *J*=4.6, 4H), 8.27 – 8.20 (m, 6H), 7.94 – 7.91 (m, 2H), 7.86 – 7.80 (m, 2H), 7.68 – 7.63 (m, 2H), 7.34 (s, 1H), 7.31 (s, 4H), 2.62 (s, 6H), 2.44 – 2.39 (m, 6H), 2.28 – 2.22 (m, 6H), 1.86 (s, 12H), 1.48 (s, 27H), 1.37 (s, 12H).

^{13}C NMR (126 MHz, THF-d_8) δ (ppm) = 173.12, 171.51, 167.00, 150.59, 150.48, 150.42, 146.97, 144.72, 140.50, 139.73, 138.02, 135.72, 135.47, 135.36, 134.92, 132.52, 132.44, 131.35, 130.91, 130.80, 130.28, 128.41, 126.89, 126.12, 123.09, 120.06, 119.96, 119.46, 84.57, 80.21, 58.66, 30.89, 30.51, 30.32, 30.10, 28.25, 28.11, 21.86, 21.47.

HRMS (MALDI-TOF, dctb) m/z for $C_{87}H_{94}BN_5O_9Zn$: [M]+ calculated: 1427.6431; found: 1427.6444

Tetra (*t*Bu-Newkome-G1 zinc porphyrin)-PBI pentad (14)

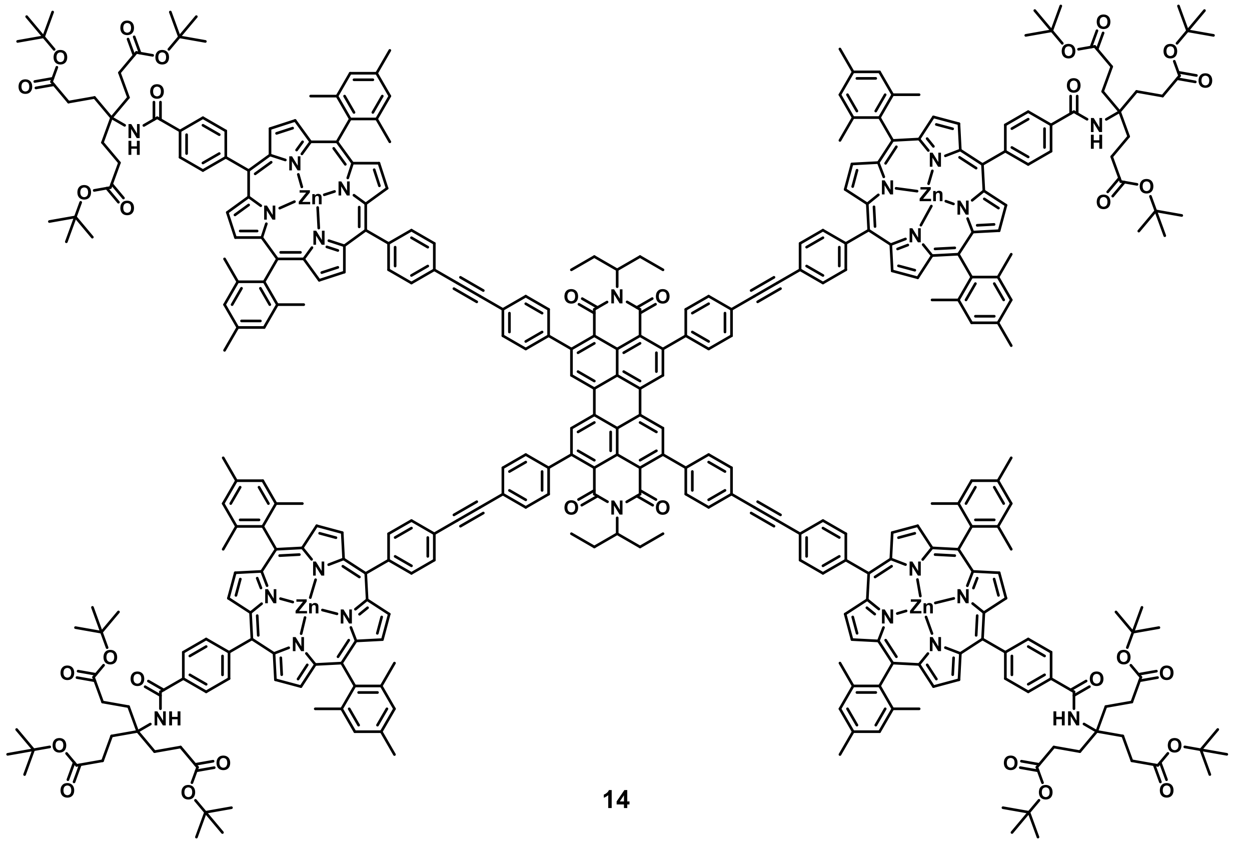

14

To a 10 mL microwave vial, borylated-porphyrin **13** (230 mg, 161.0 µmol, 6.7 eq.), *tetra*-iodo-PBI **7** (25 mg, 24.4 µmol), Cs_2CO_3 (47 mg, 143 µmol, 6 eq.) and $Pd(PPh_3)_4$ (11 mg, 9.8 µmol, 0.4 eq.) were added, and the vial evacuated and backfilled with nitrogen three times. Then, a degassed mixture of toluene and DMF (6 mL / 3 mL) was added, and the reaction mixture was heated to 60 °C for 16 h. Subsequently, the reaction mixture was allowed to cool to room temperature and was then passed through a short silica plug (eluent: Tol/THF 5:1). The crude mixture was purified by multiple manual SEC (BioBeads SX1, 2x ($CHCl_3$ Ø 2.5 cm, ↑ 60 cm) and 2x (Toluene Ø 5 cm, ↑ 120 cm), followed by another silica plug filtration (eluent Tol/THF 5:1) yielding the desired porphyrin-perylene-pentad **14** as a purple-red solid (62 mg, 10.8 µmol, 44 %)

^{1}H NMR (600 MHz, THF-*d*$_8$) δ (ppm) = 8.84 – 8.79 (m, 4H), 8.69 (d, *J*=4.6, 4H), 8.27 – 8.20 (m, 6H), 7.94 – 7.91 (m, 2H), 7.86 – 7.80 (m, 2H), 7.68 – 7.63 (m, 2H), 7.34 (s, 1H), 7.31 (s, 4H), 2.62 (s, 6H), 2.44 – 2.39 (m, 6H), 2.28 – 2.22 (m, 6H), 1.86 (s, 12H), 1.48 (s, 27H), 1.37 (s, 12H).

^{13}C NMR (151 MHz, THF-*d*$_8$) δ (ppm) = 173.12, 171.51, 167.00, 150.59, 150.48, 150.42, 146.97, 144.72, 140.50, 139.73, 138.02, 135.72, 135.47, 135.36, 134.92, 132.52, 132.44,

131.35, 130.91, 130.80, 130.28, 128.41, 126.89, 126.12, 123.09, 120.06, 119.96, 119.46, 84.57, 80.21, 58.66, 30.89, 30.51, 30.32, 30.10, 28.25, 28.11, 21.86, 21.47.

MS (MALDI-TOF, dctb) *The monoisotopic peak was not observed with high resolution due to the high molar mass and the resulting isotopic pattern. The obtained spectrum is given in **Figure S65**.*

UV/Vis (THF): $\varepsilon_{(\lambda max)}$ = 1710000 (427), 57 000 (492), 69 000 (528), 80 000 (558), 32 000 (599) L*mol^{-1}*cm^{-1} (nm).

Tetra (COOH-Newkome-G1 zinc porphyrin)-PBI pentad (15)

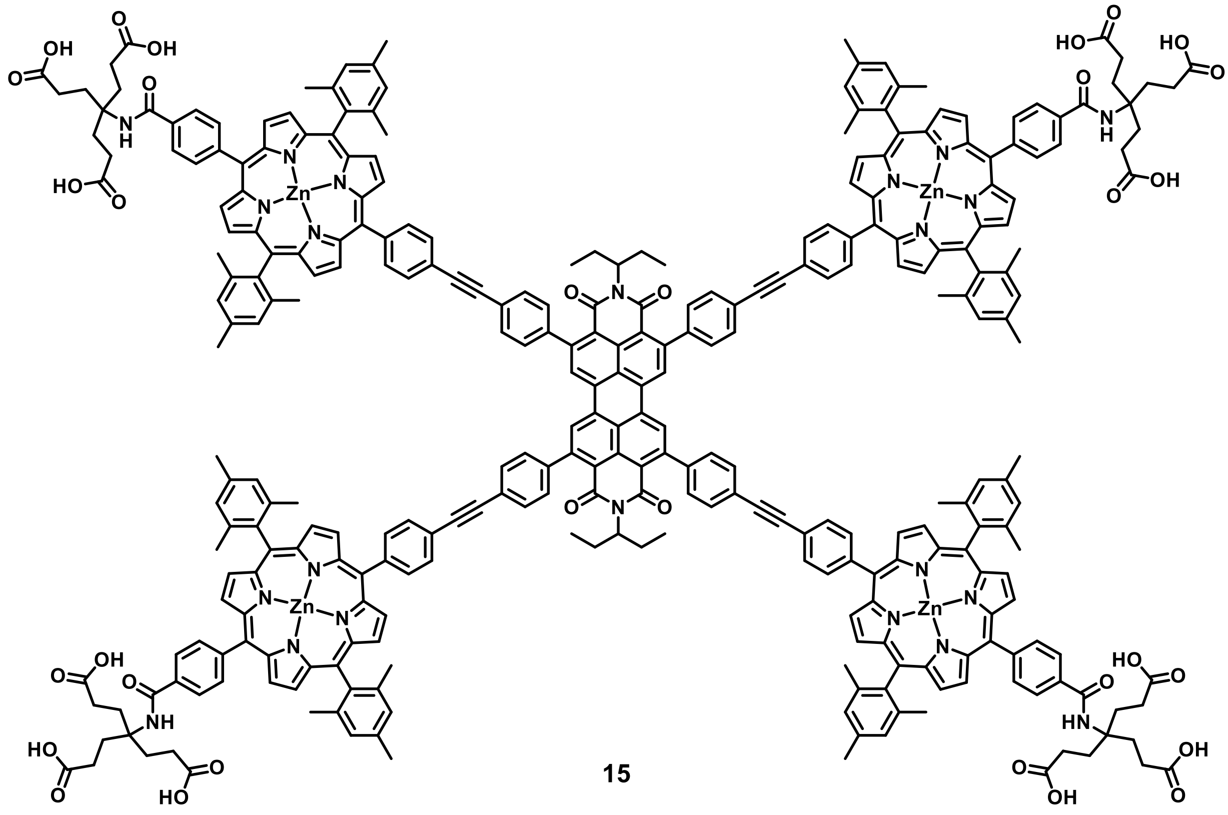

15

In a 10 mL microwave vial, **14** (27 mg, 4.7 µmol) was dissolved in formic acid (5 mL) under a nitrogen atmosphere. The reaction mixture was stirred at rt. for 3 d. Then, the solvent was removed by azeotropic co-evaporation with toluene (3x 15 mL) and the remaining solid was redispersed in THF (3 mL). $Zn(OAc)_2$ (0.7 mg, 3.76 µmol, 8 eq.) was added and the mixture was heated to 70 °C to re-metalate possible free-base porphyrin. After 48 h, the precipitate was collected by vacuum filtration and washed with H_2O (2x 20 mL) and MeOH (20 mL). The remaining solid was then redissolved in THF / TFA (1 vol%) followed by immediate precipitation with *n*-pentane. Collection via vacuum filtration gave the desired deprotected pentad as a dark red powder (11 mg, 2.18 µmol, 46 %)

^{1}H NMR: (600 MHz, THF-d_8) δ = 8.92 (s, 4H), 8.90 – 8.86 (m, 8H), 8.86 – 8.80 (m, 8H), 8.76 – 8.68 (m, 16H), 8.26 (s, 48H), 8.03 – 7.99 (m, 8H), 7.89 – 7.84 (m, 8H), 7.69 – 7.65 (m, 8H), 7.54 (s, 3H), 7.34 (s, 4H), 7.31 (s, 16H), 2.68 – 2.63 (m, 4H), 2.61 (s, 12H), 2.53 – 2.46 (m, 24H), 2.38 – 2.31 (m, 28H), 1.87 (s, 48H), 0.95 – 0.92 (m, 12H).

^{13}C NMR: *Due to the instability of the molecule towards the acidic conditions over the prolonged times required for the measurement, we were not able to acquire a suitable carbon NMR spectrum*

MS (MALDI-TOF, dctb) *The monoisotopic peak was not observed with high resolution due to the high molar mass and the resulting isotopic pattern. The obtained spectrum is given in* ***Figure S66****.*

UV/Vis (THF/10 mM NaOH (aq) 1:1 v/v): $\varepsilon_{(\lambda max)}$ = 1370000 (426), 58 000 (492), 66 000 (528), 73 000 (558), 30 000 (599) L*mol^{-1}*cm^{-1} (nm).

2. NMR Spectra

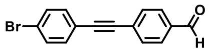

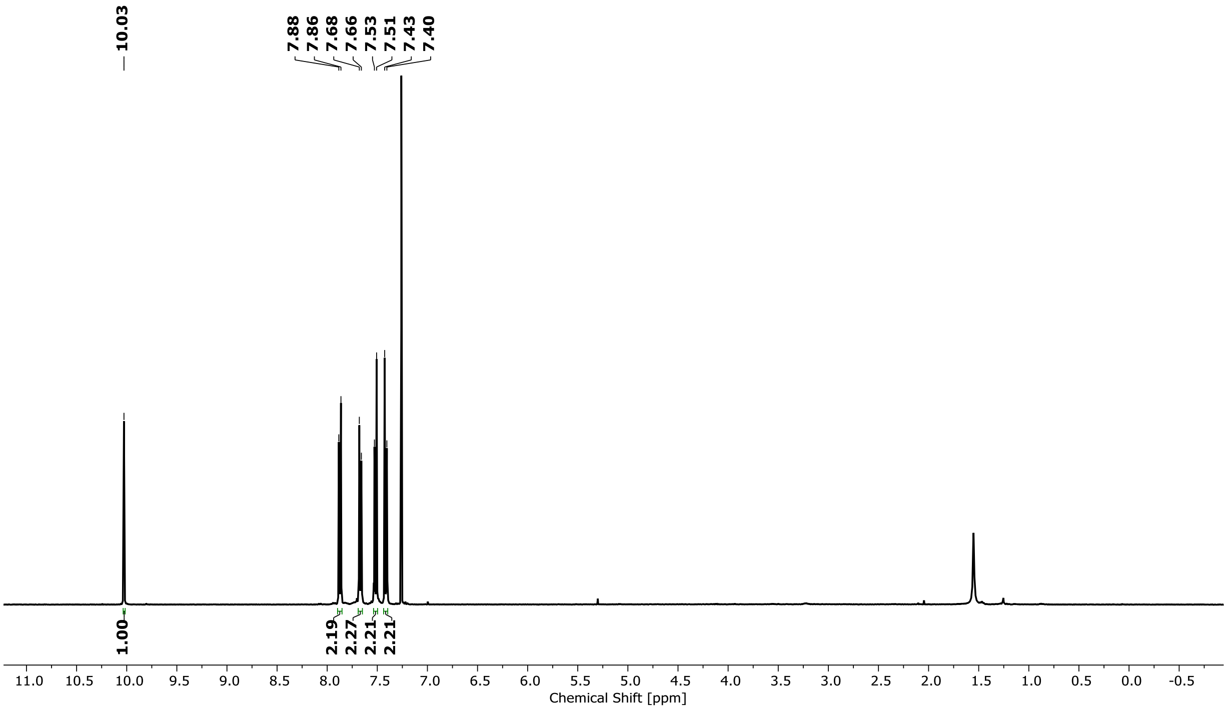

Figure S1 ^{1}H NMR (400 MHz, $CDCl_3$, rt) of **Bromo-tolane-aldehyde**

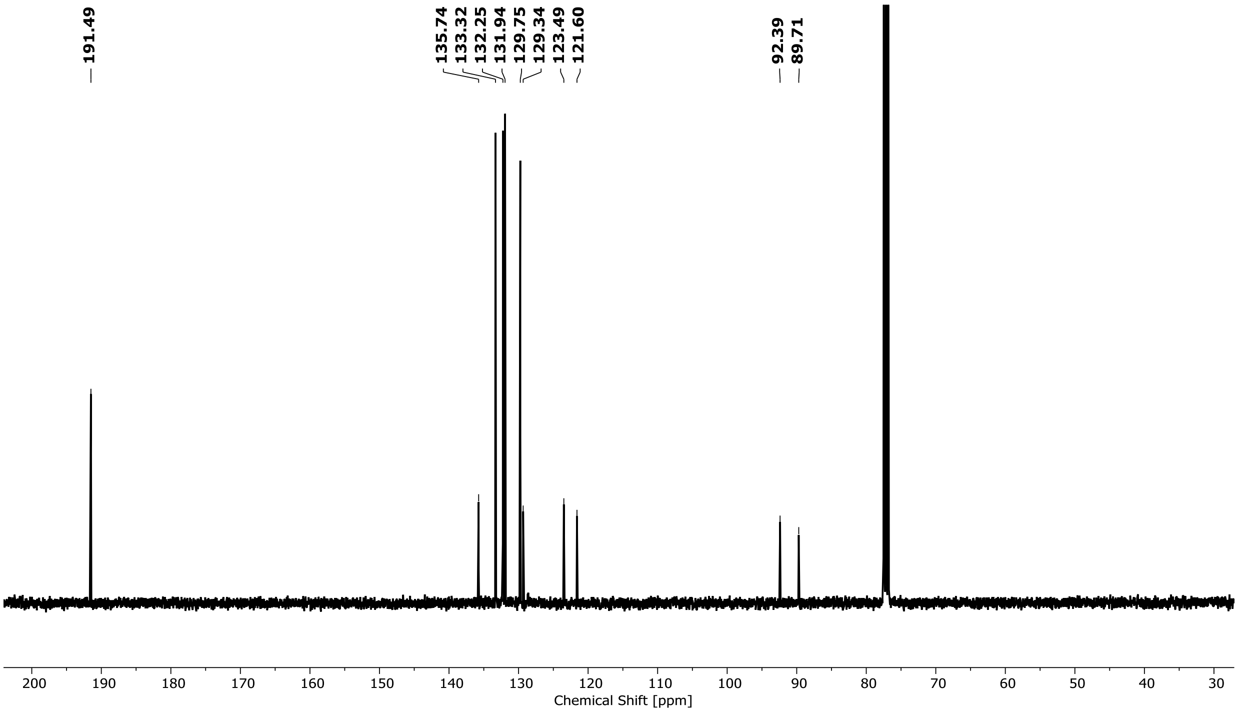

Figure S2 ^{13}C NMR (101 MHz, $CDCl_3$, rt) of **Bromo-tolane-aldehyde**

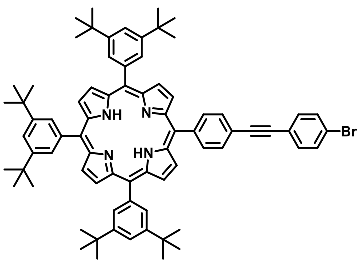

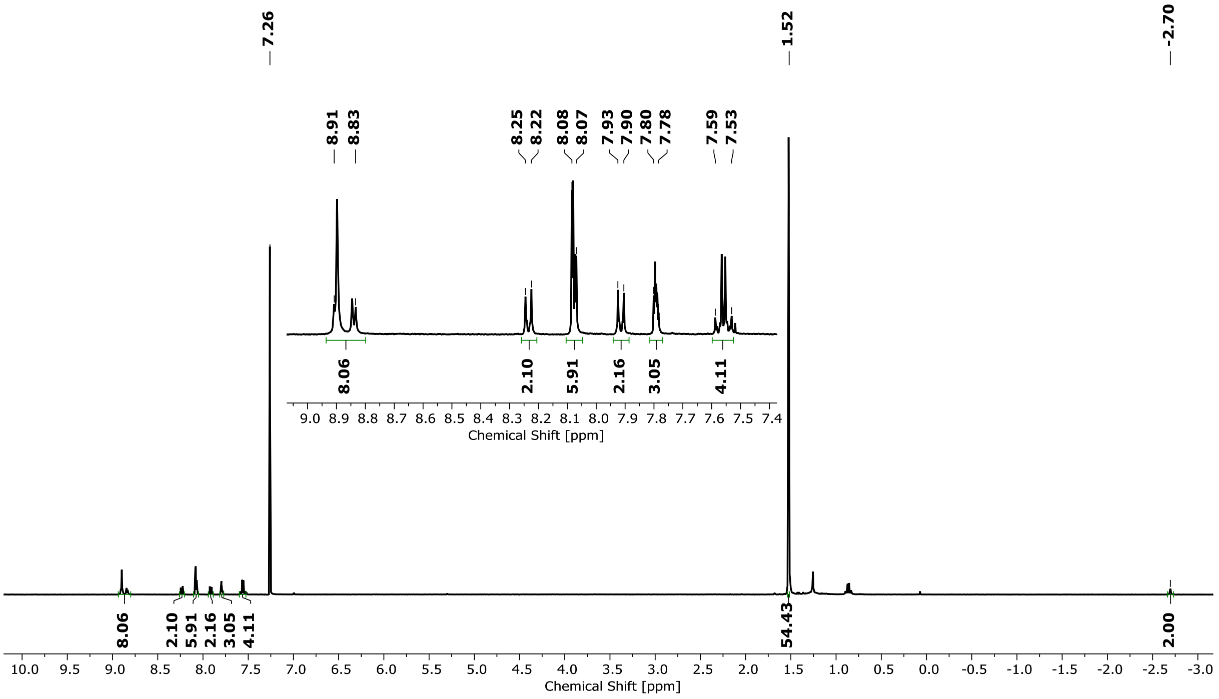

Figure S3 ^{1}H NMR (400 MHz, $CDCl_3$, rt) of **1**

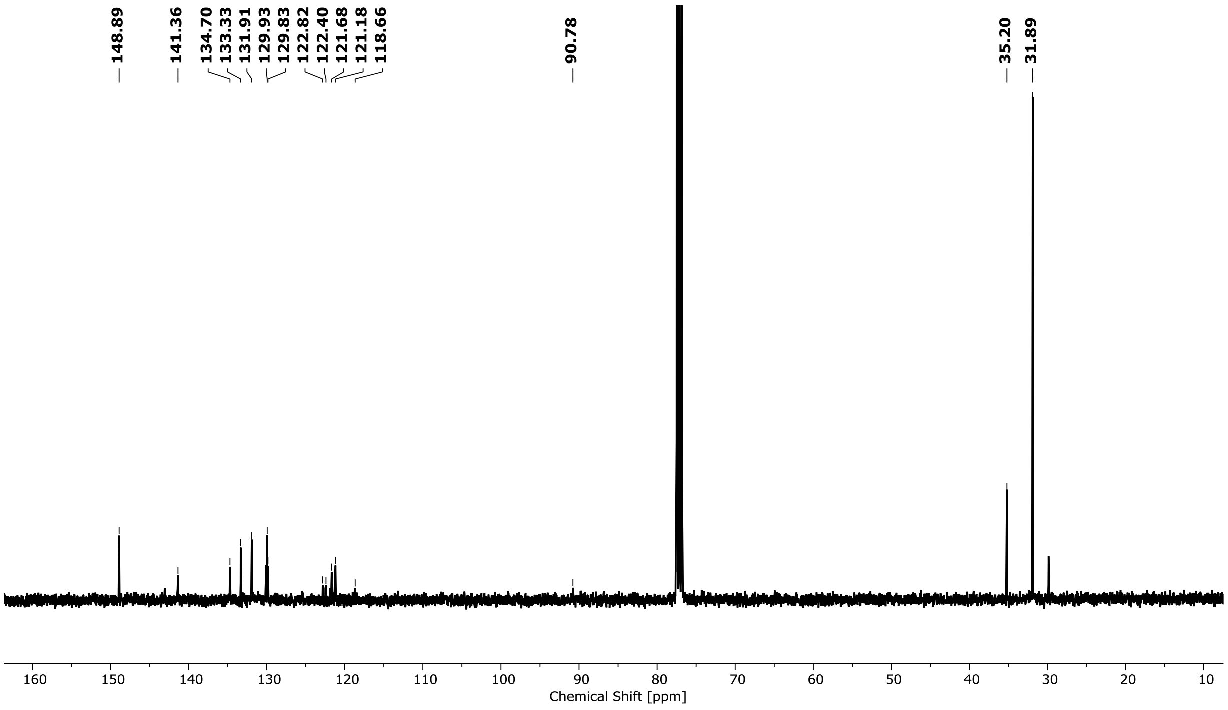

Figure S4 ^{13}C NMR (101 MHz, $CDCl_3$, rt) of **1**

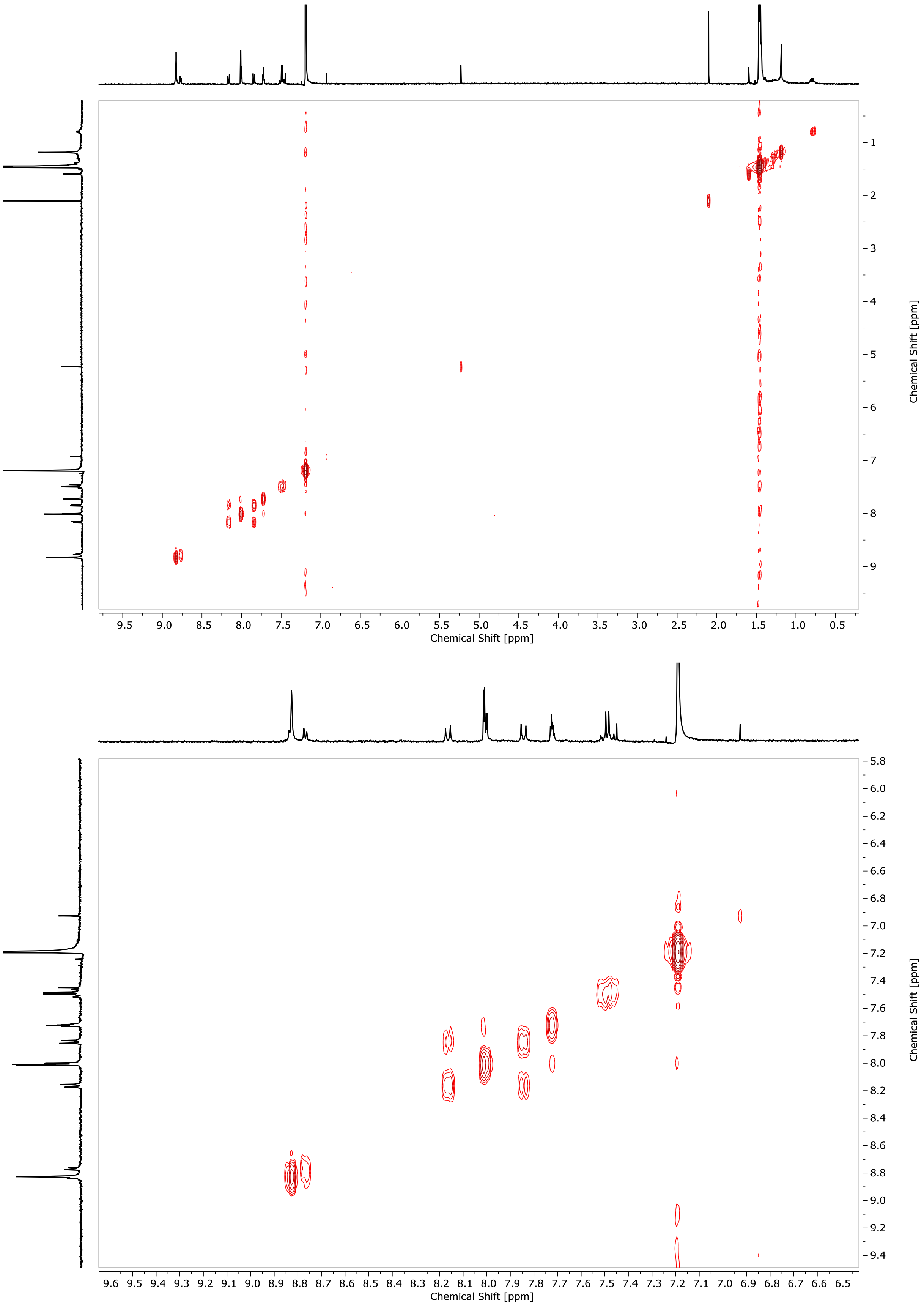

Figure S5 COSY NMR ($CDCl_3$) of **1**

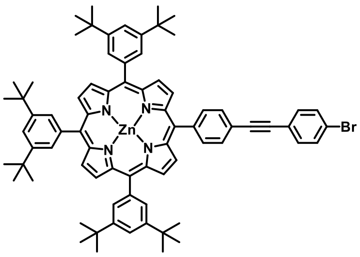

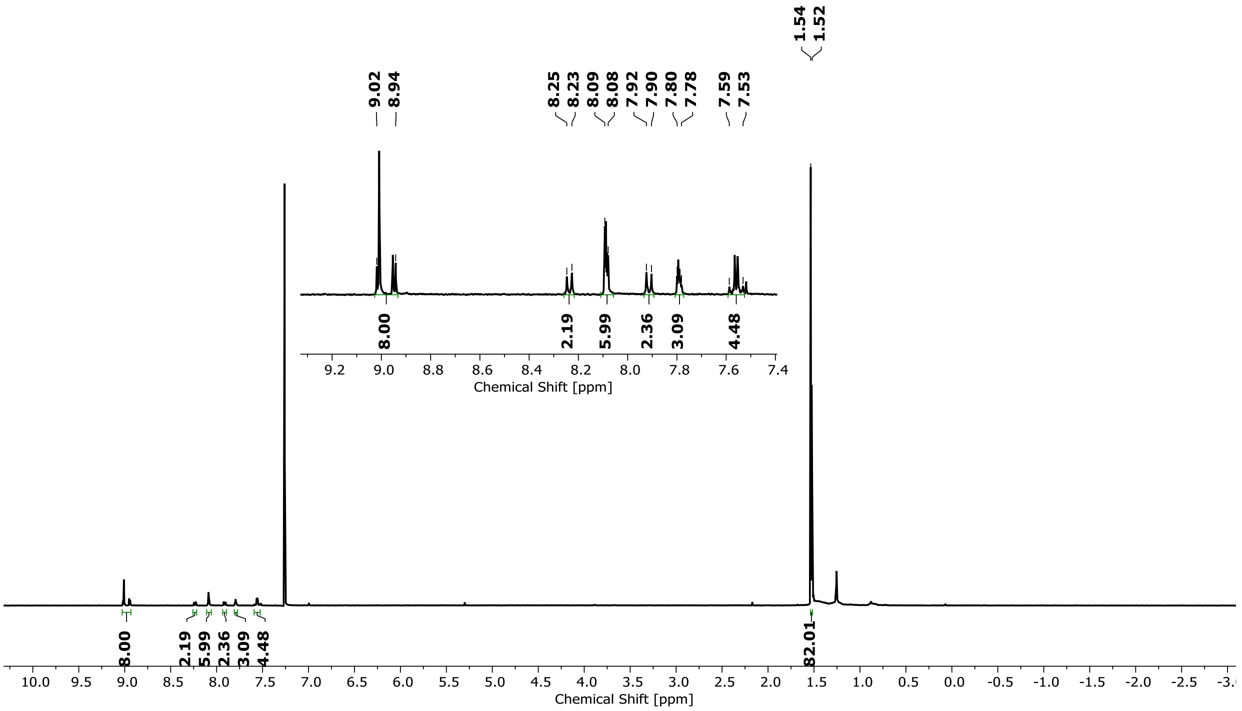

Figure S6 ^{1}H NMR (400 MHz, $CDCl_3$, rt) of **2**

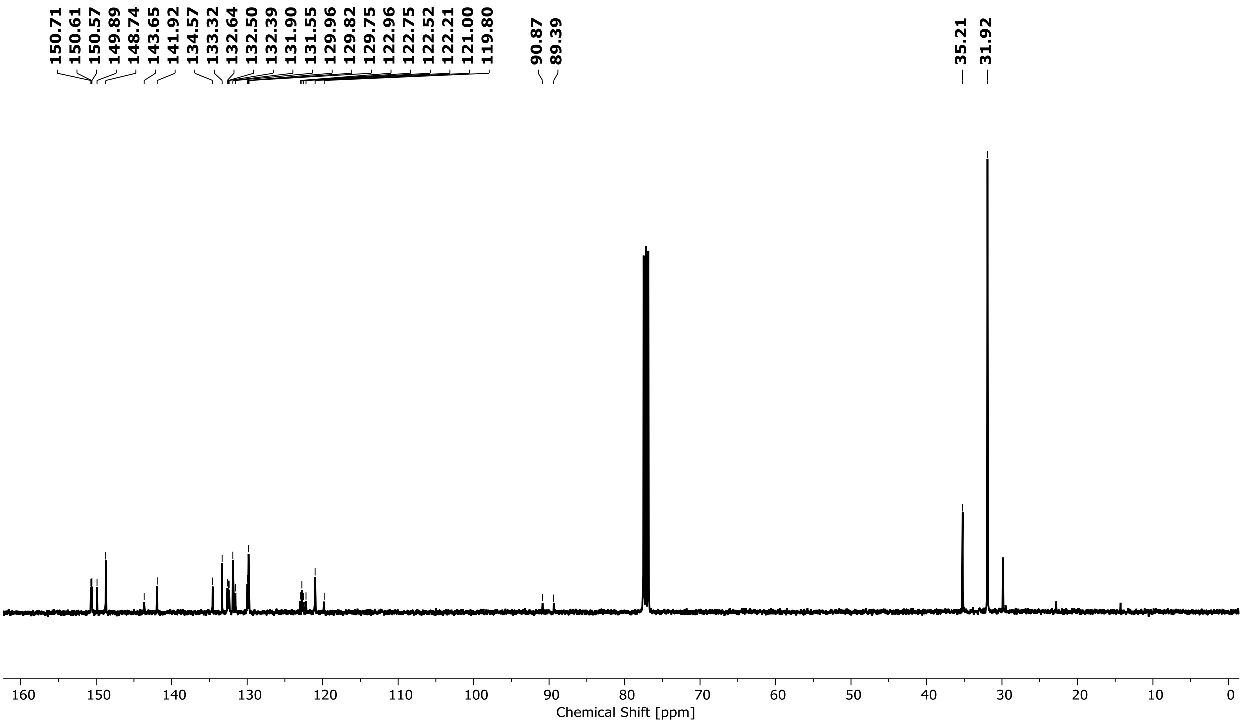

Figure S7 ^{13}C NMR (101 MHz, $CDCl_3$, rt) of **2**

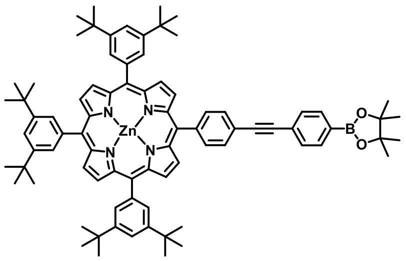

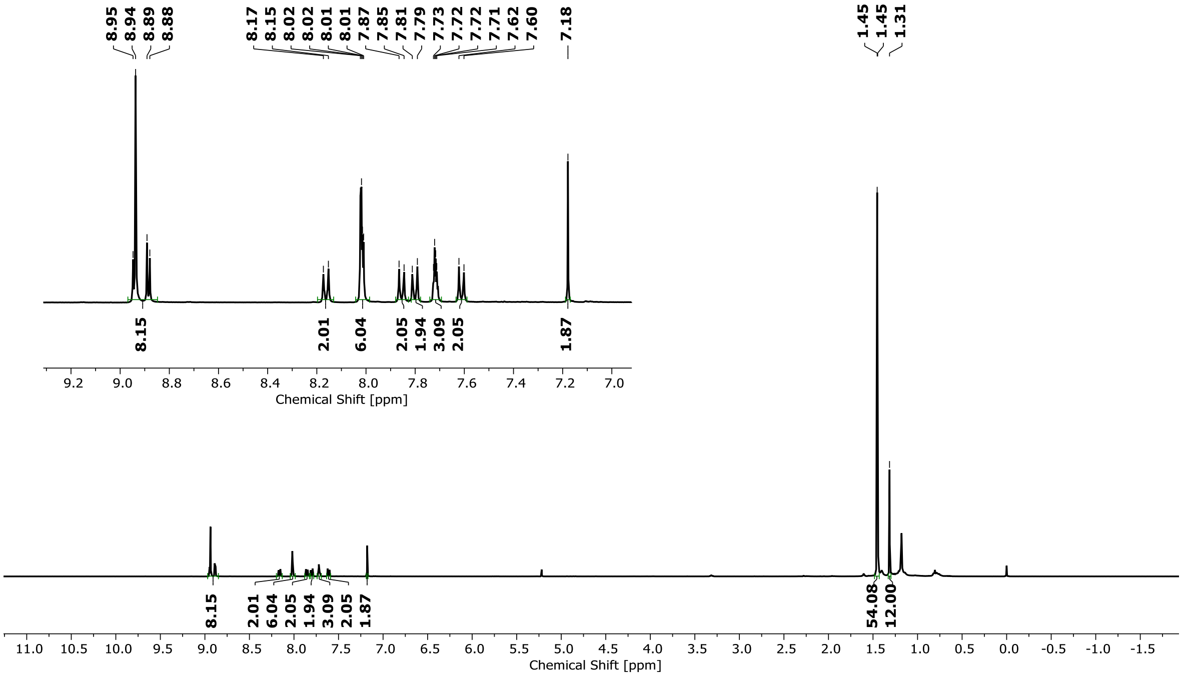

Figure S8 ^{1}H NMR (400 MHz, $CDCl_3$, rt) of **3**

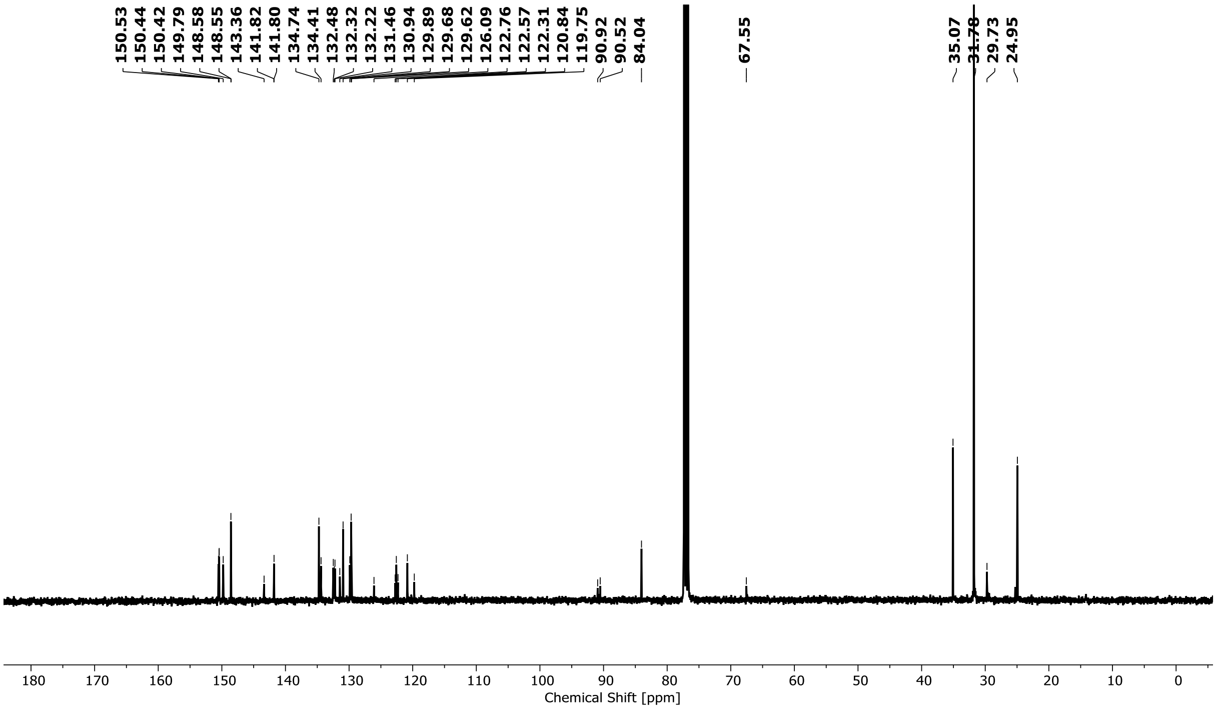

Figure S9 ^{13}C NMR (101 MHz, $CDCl_3$, rt) of **3**

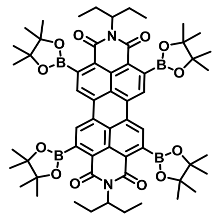

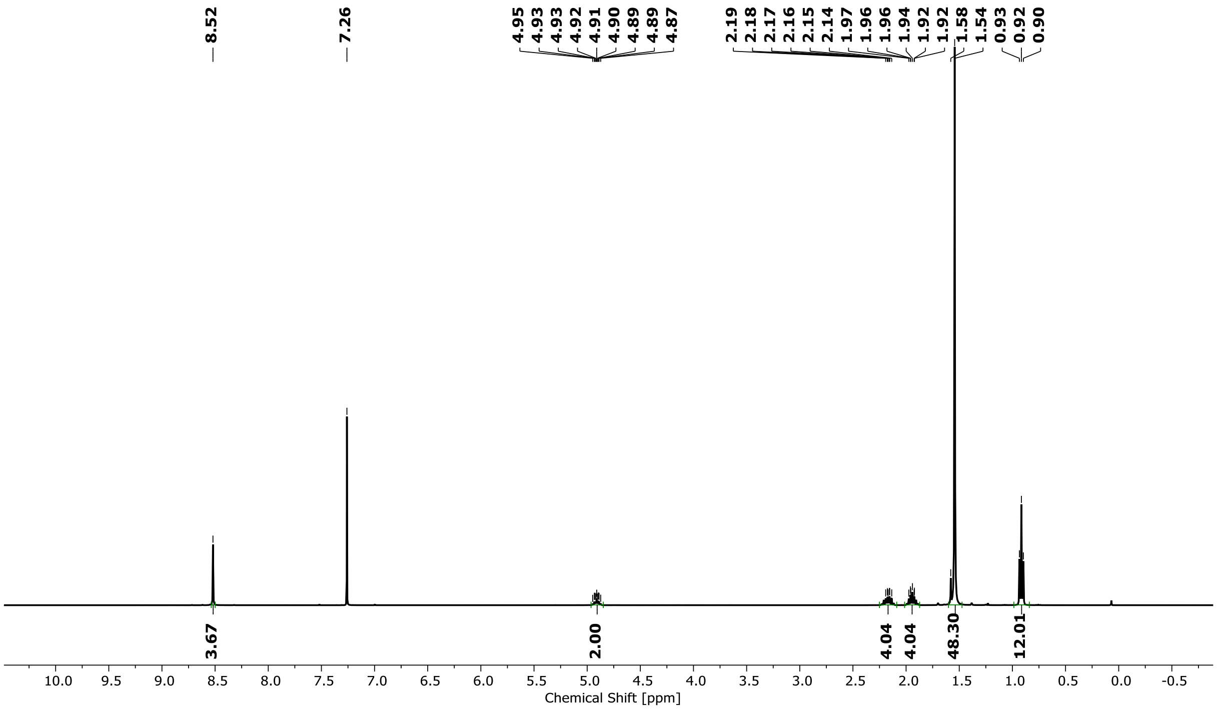

Figure S10 ^{1}H NMR (400 MHz, $CDCl_3$, rt) of **5**

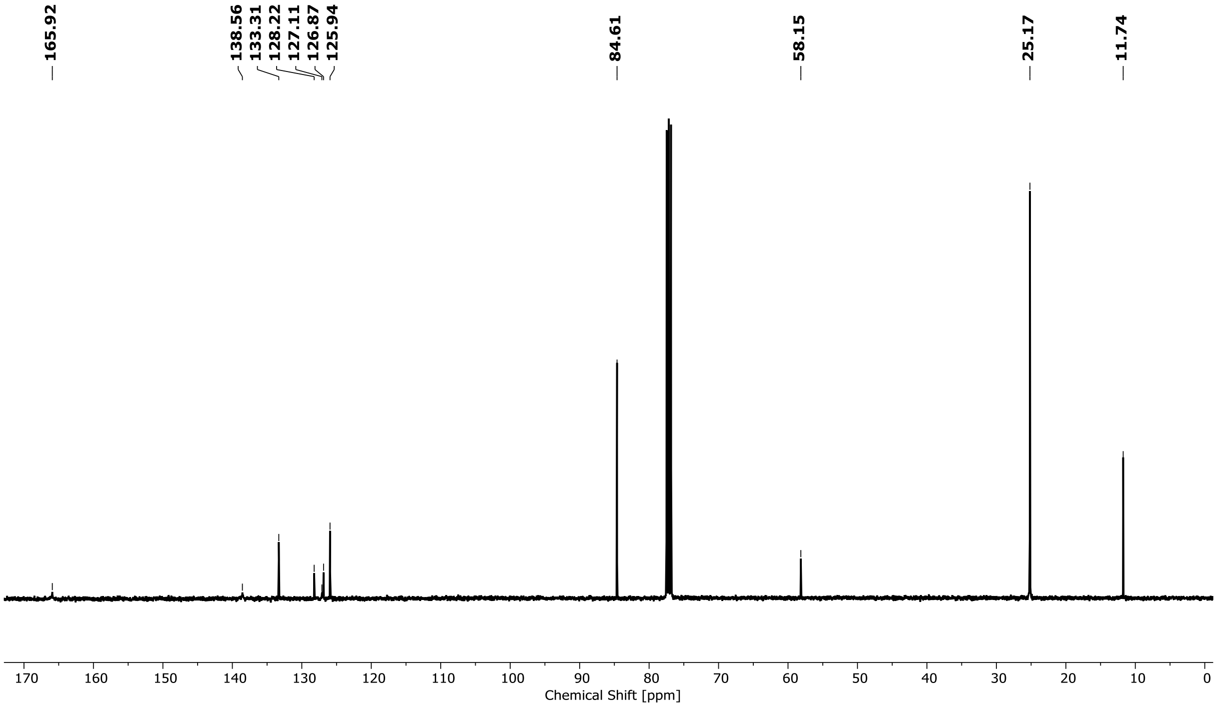

Figure S11 ^{13}C NMR (101 MHz, $CDCl_3$, rt) of **5**

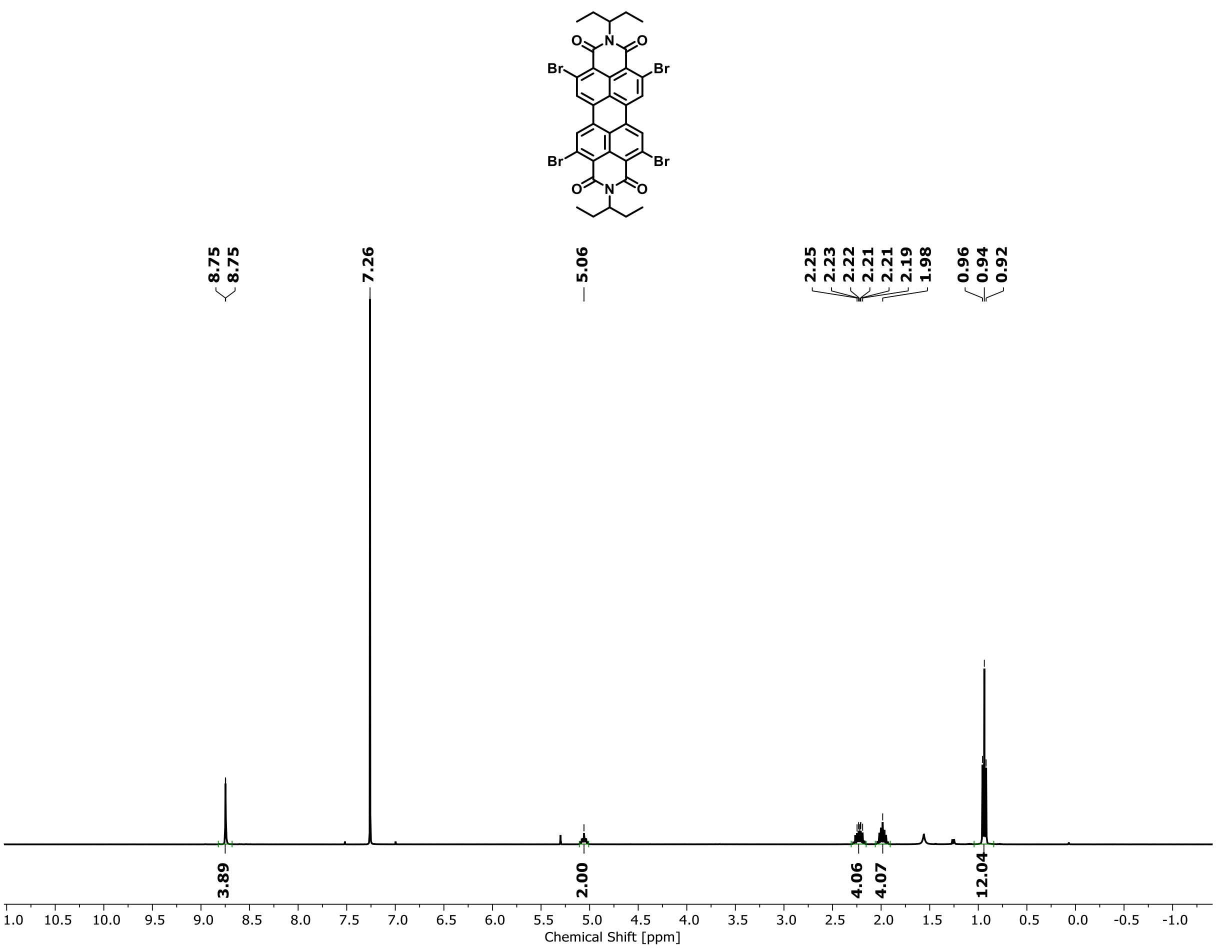

Figure S12 ^{1}H NMR (400 MHz, $CDCl_3$, rt) of **6**

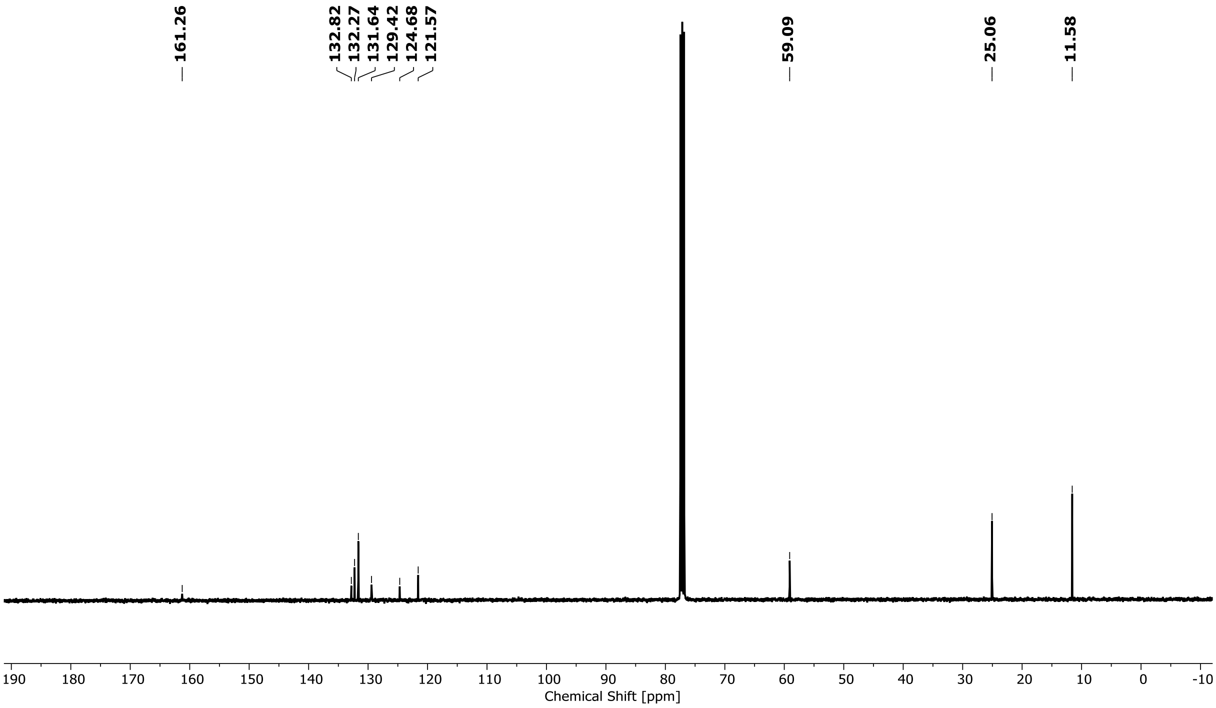

Figure S13 ^{13}C NMR (101 MHz, $CDCl_3$, rt) of **6**

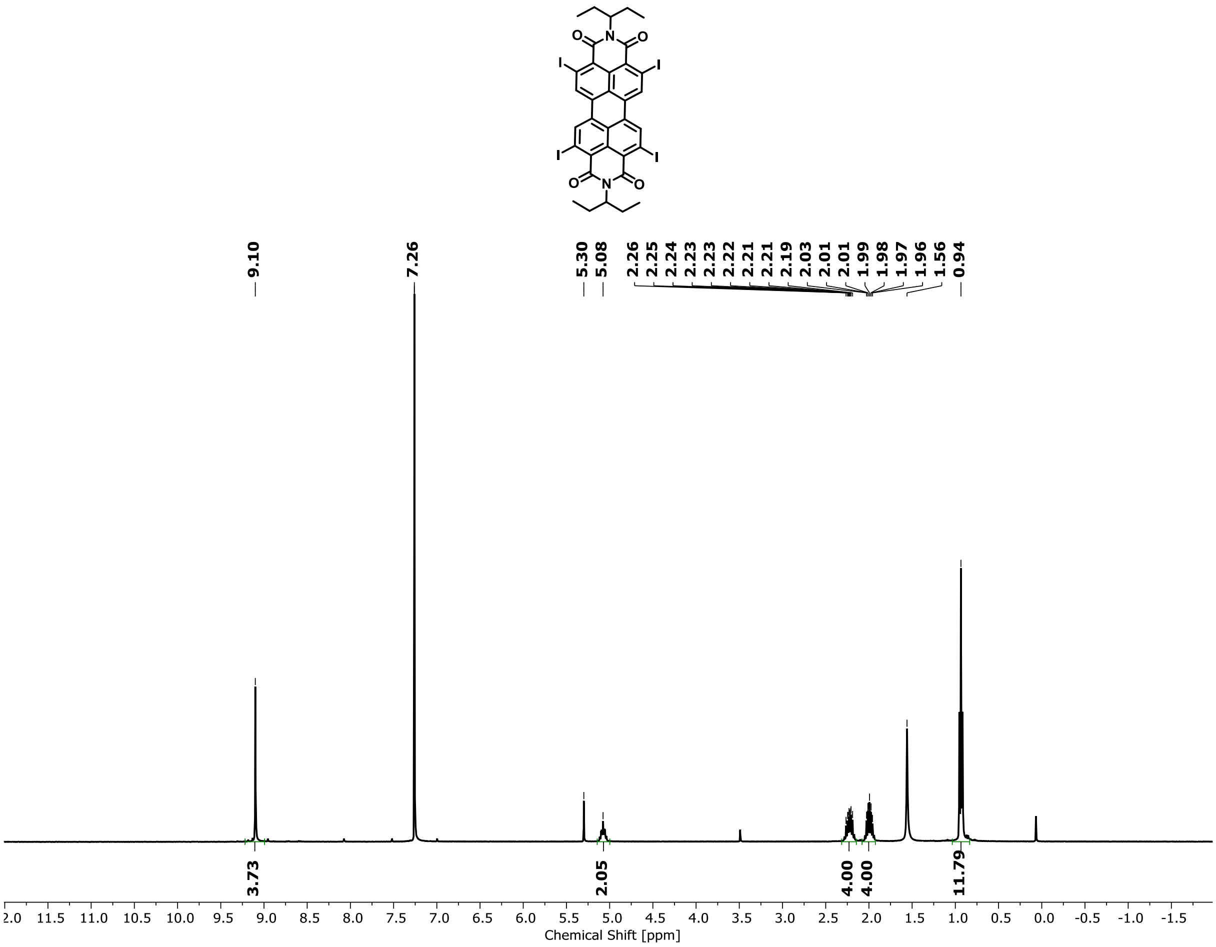

Figure S14 ^{1}H NMR (400 MHz, $CDCl_3$, rt) of **7**

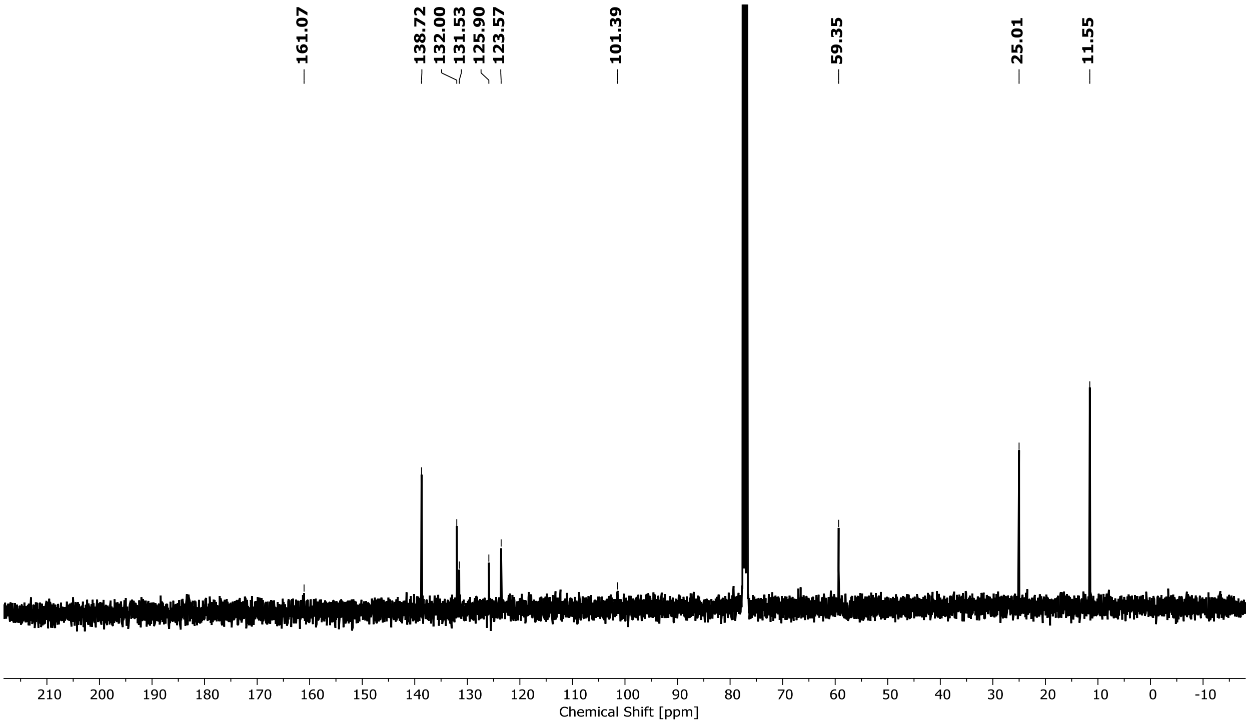

Figure S15 ^{13}C NMR (101 MHz, $CDCl_3$, rt) of **7**

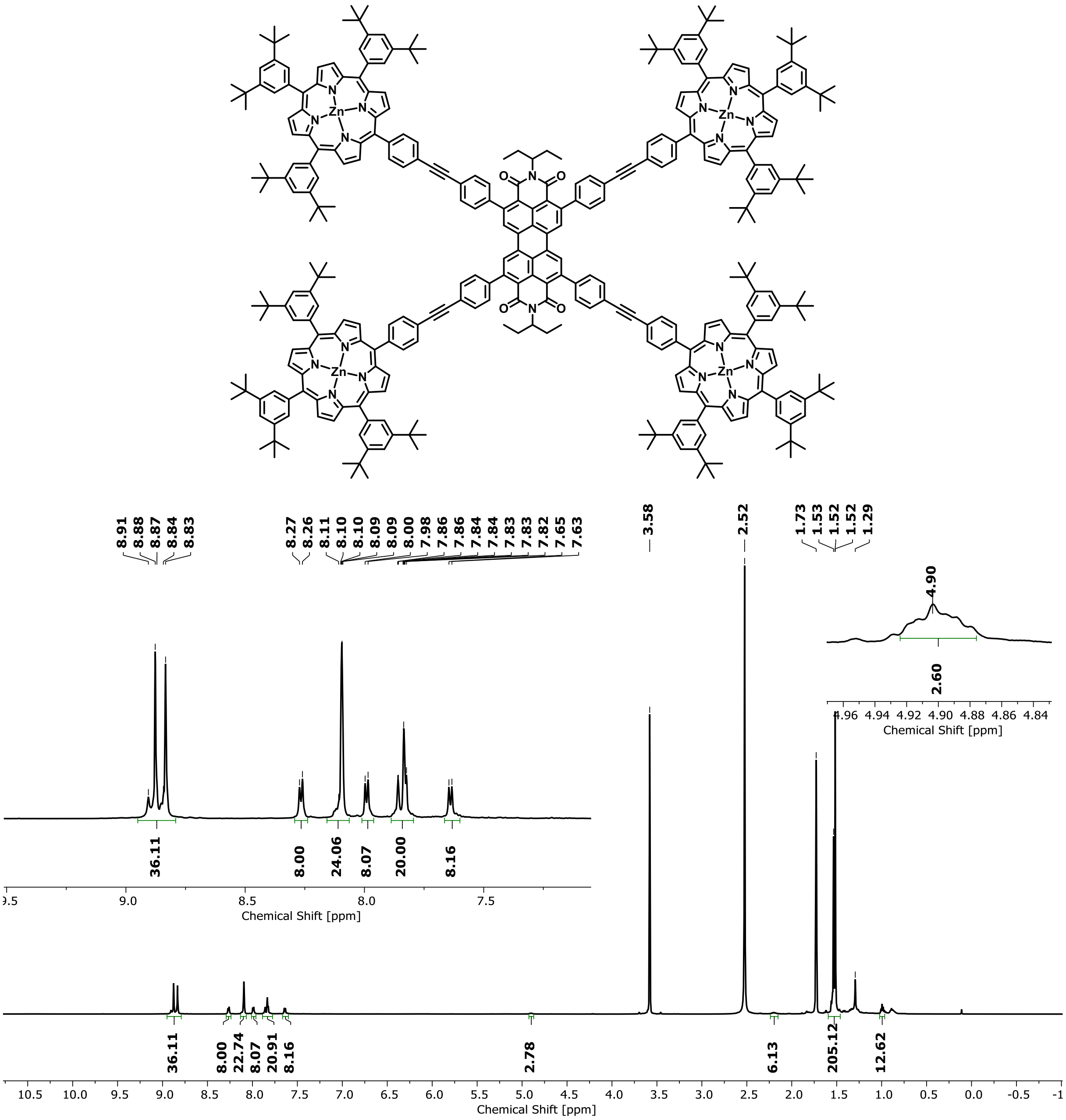

Figure S16 ^1H NMR (600 MHz, $CDCl_3$, rt) of **8**

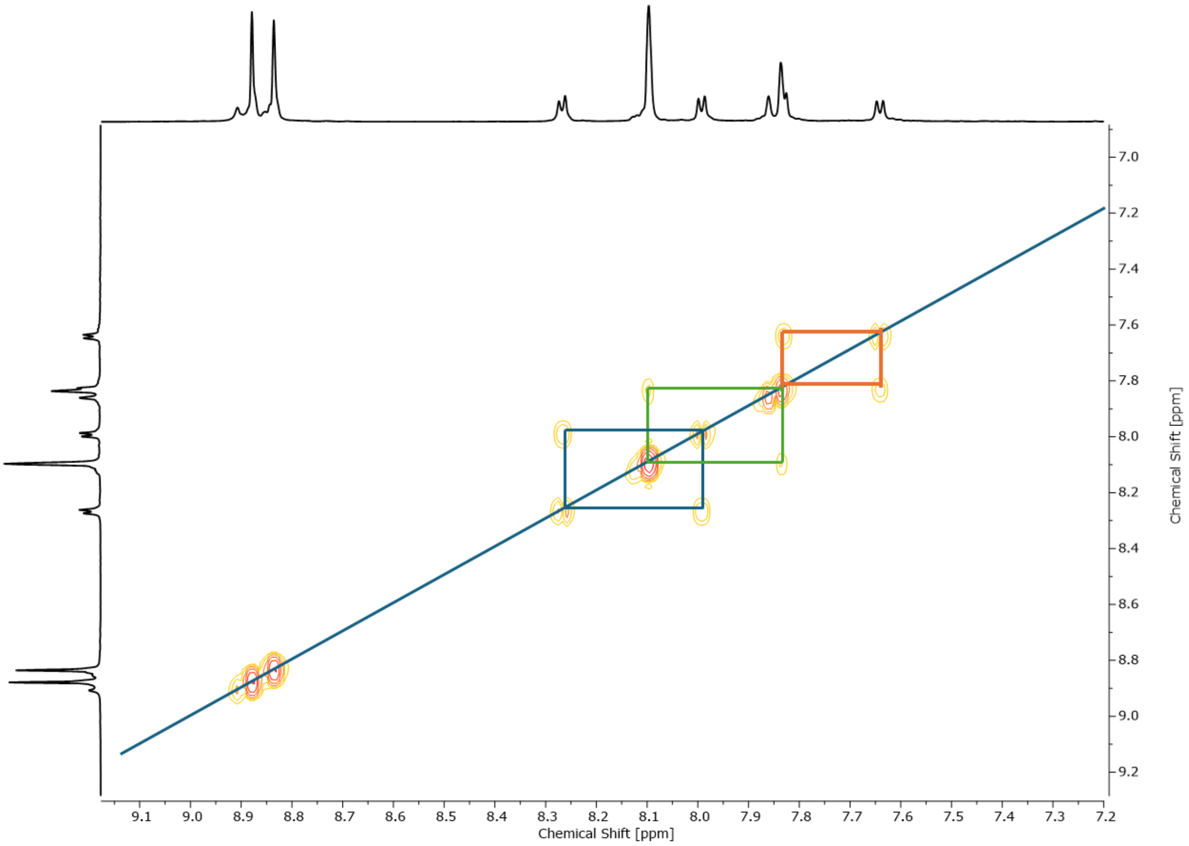

Figure S17 COSY NMR of **8**

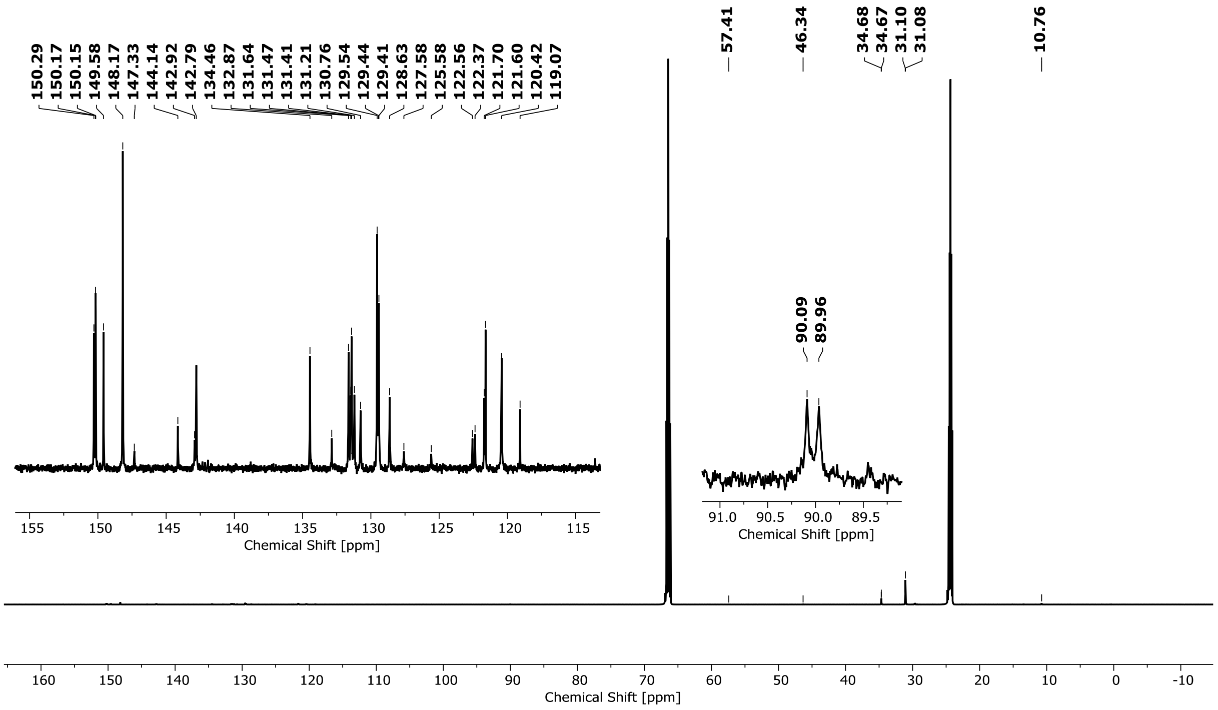

Figure S18 ^{13}C NMR (151 MHz, THF-d_8 rt) of **8**

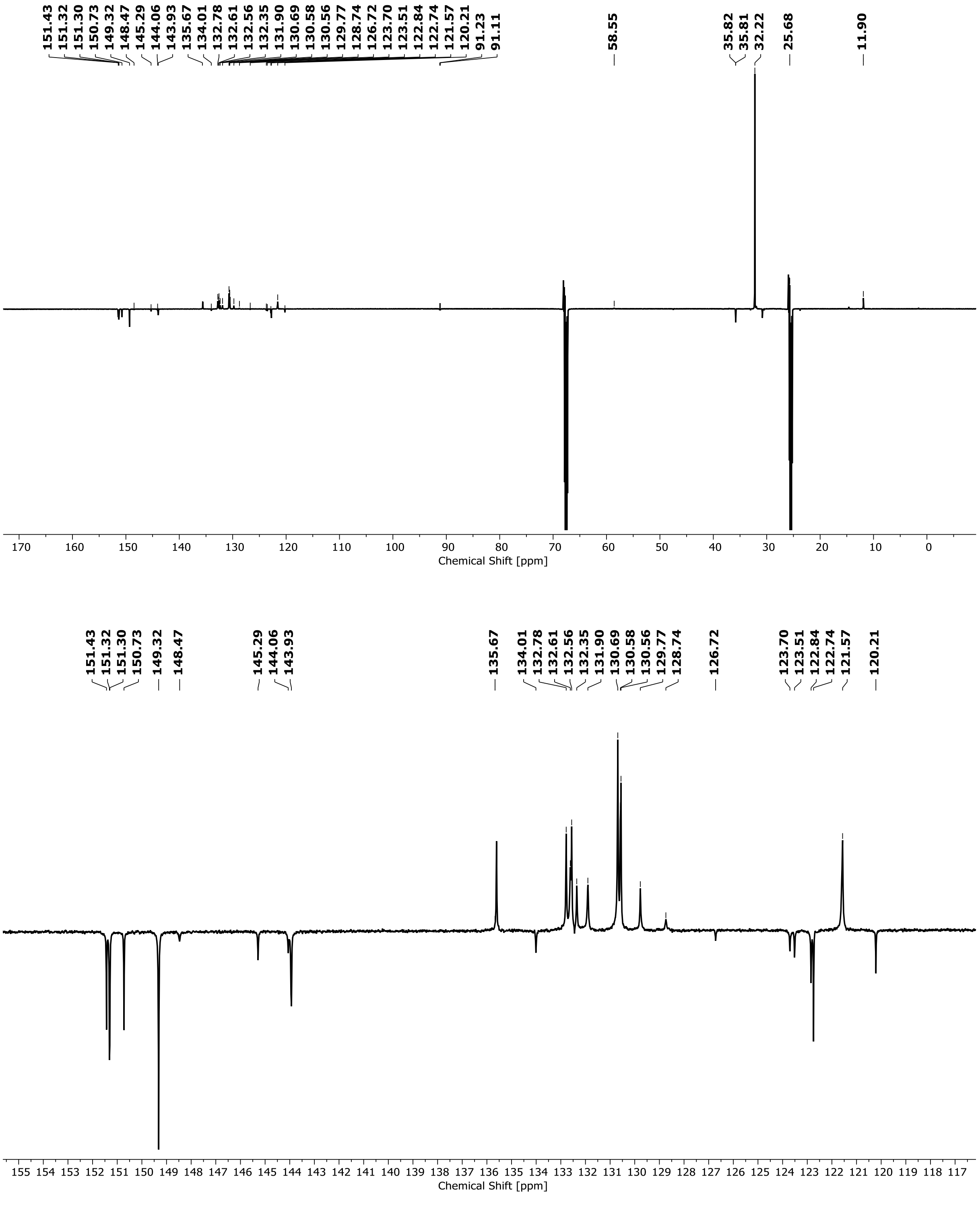

Figure S19 DEPTq NMR (151 MHz, THF-d8 rt) of **8**

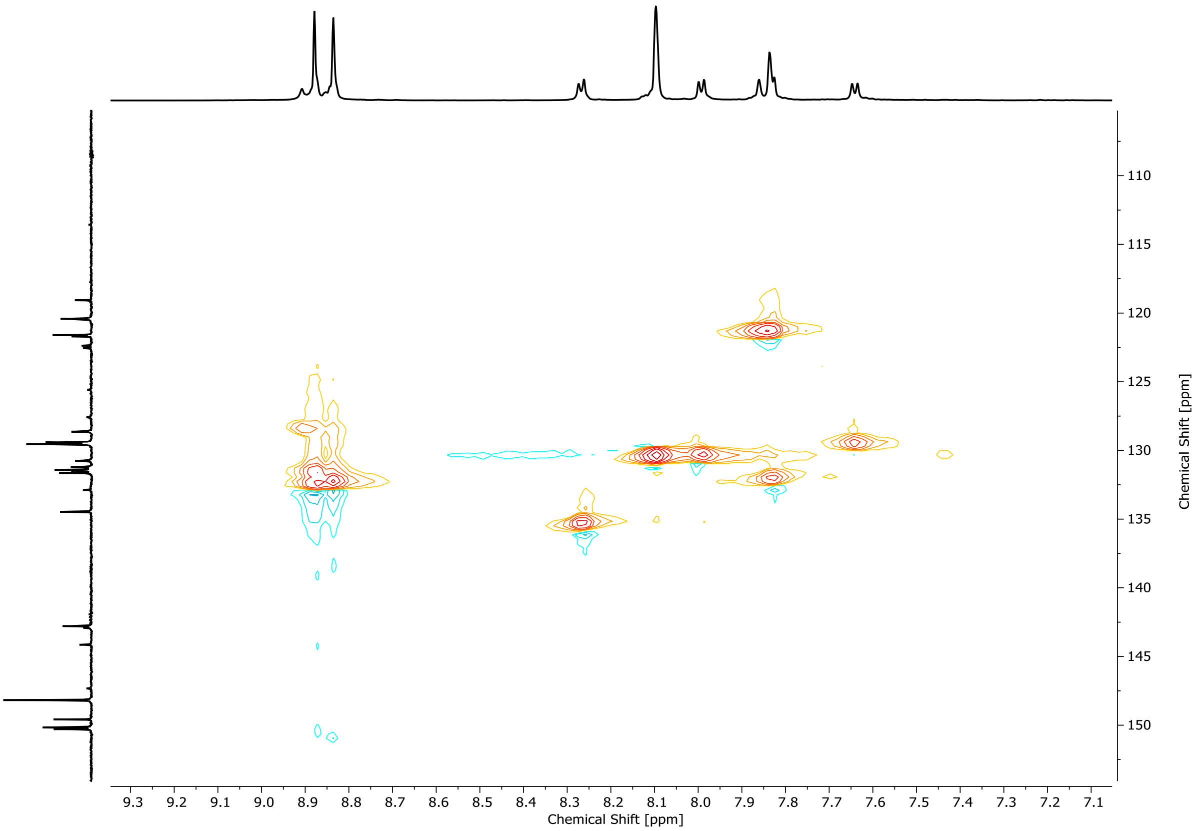

Figure S20 Partial HSQC NMR of **8** (THF-d$_8$)

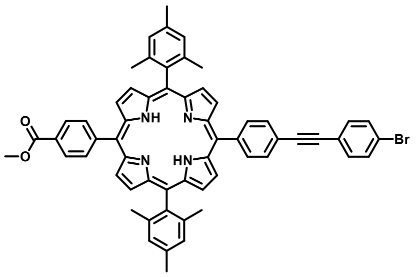

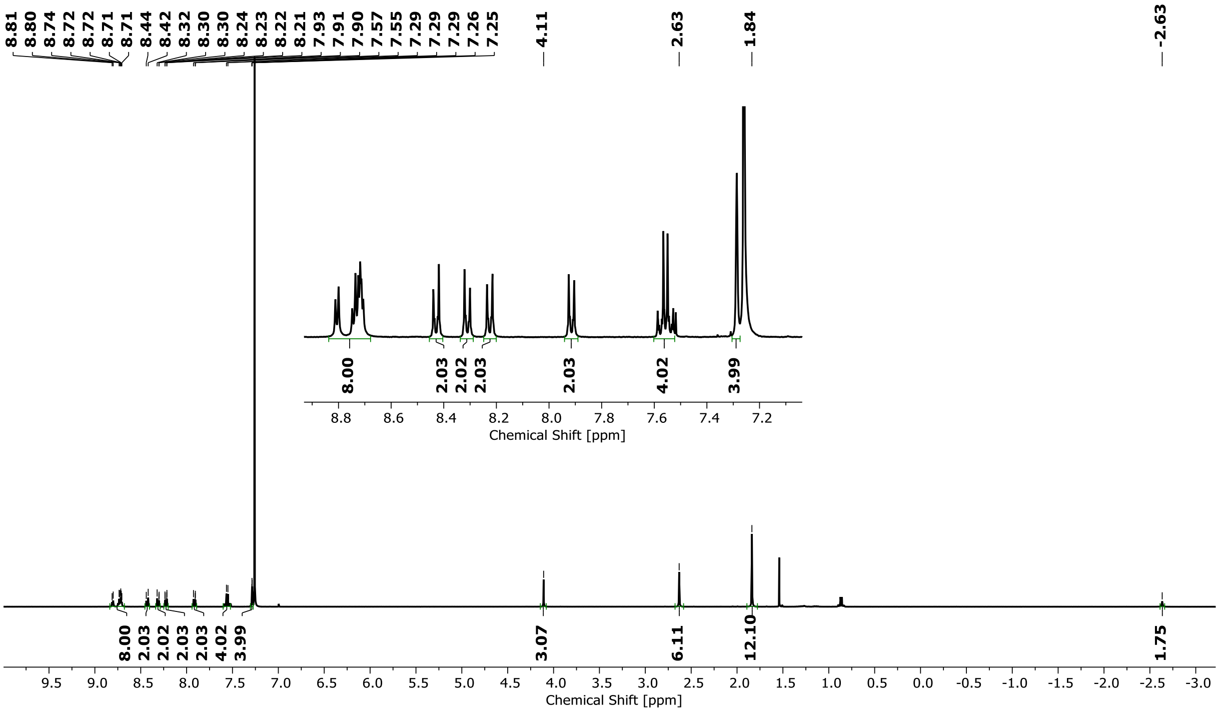

Figure S21 ^{1}H NMR (400 MHz, $CDCl_3$, rt) of **9**

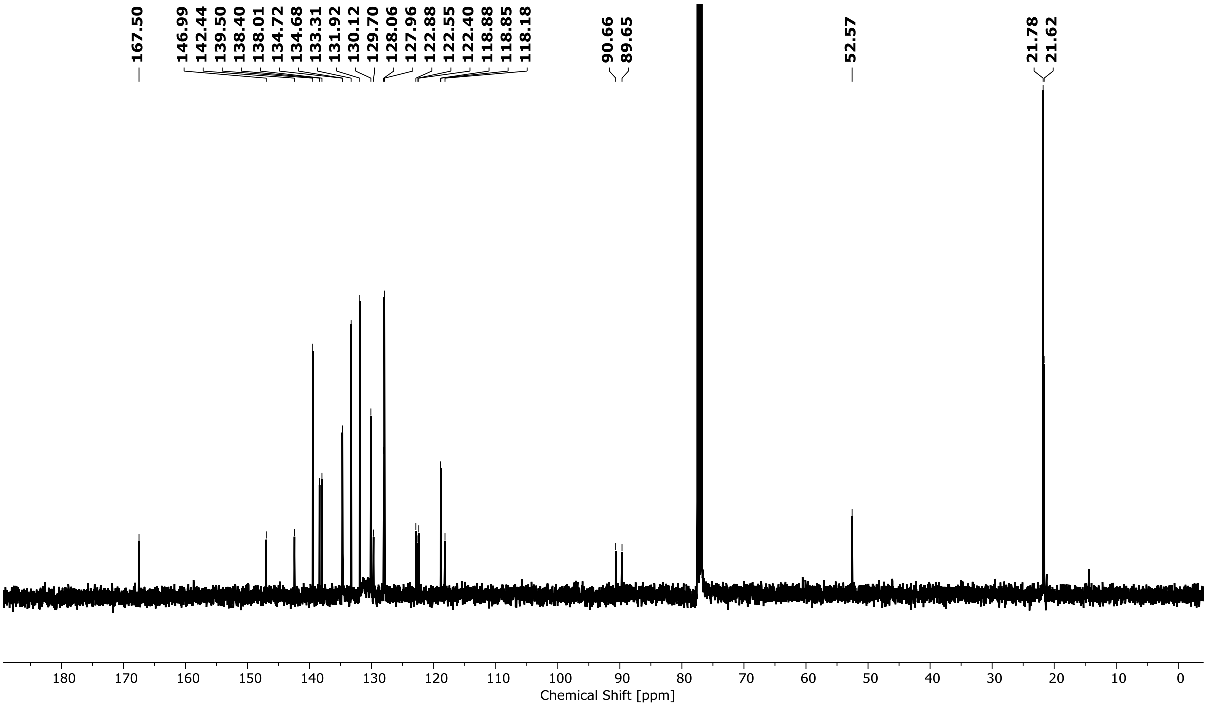

Figure S22 ^{13}C NMR (101 MHz, $CDCl_3$, rt) of **9**

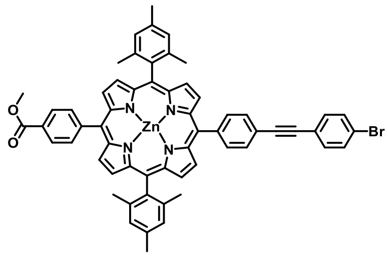

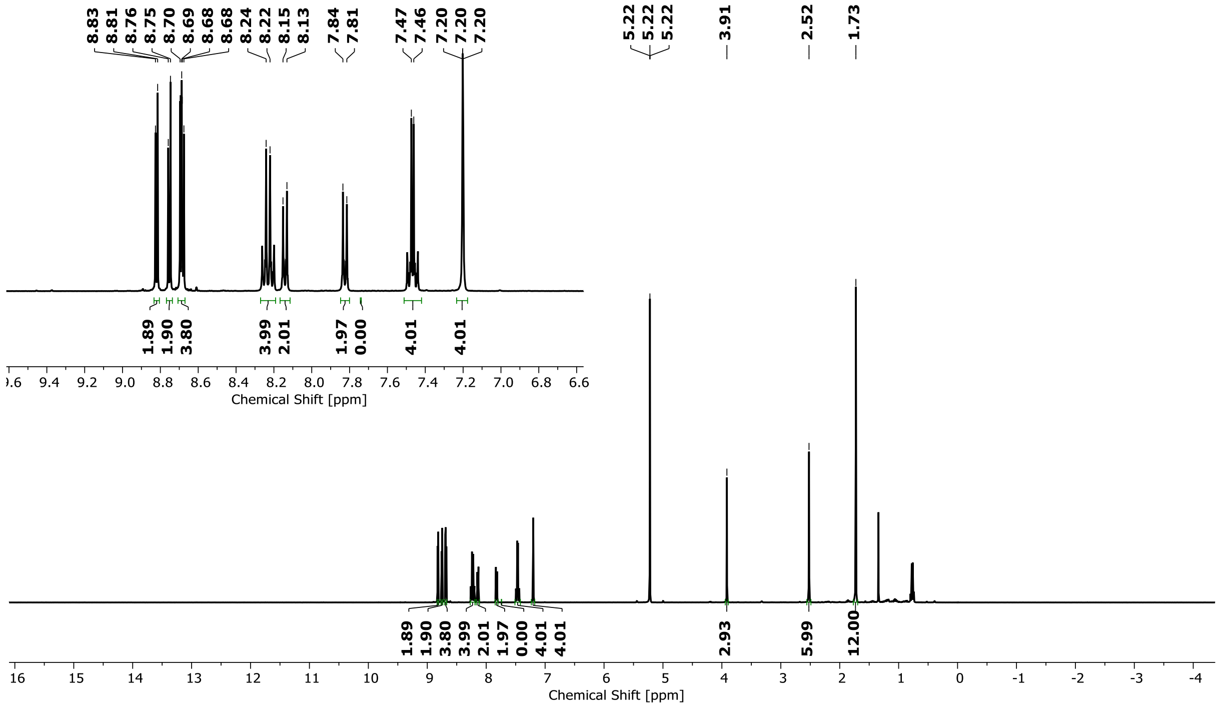

Figure S23 ^{1}H NMR (400 MHz, CD_2Cl_2, rt) of **10**

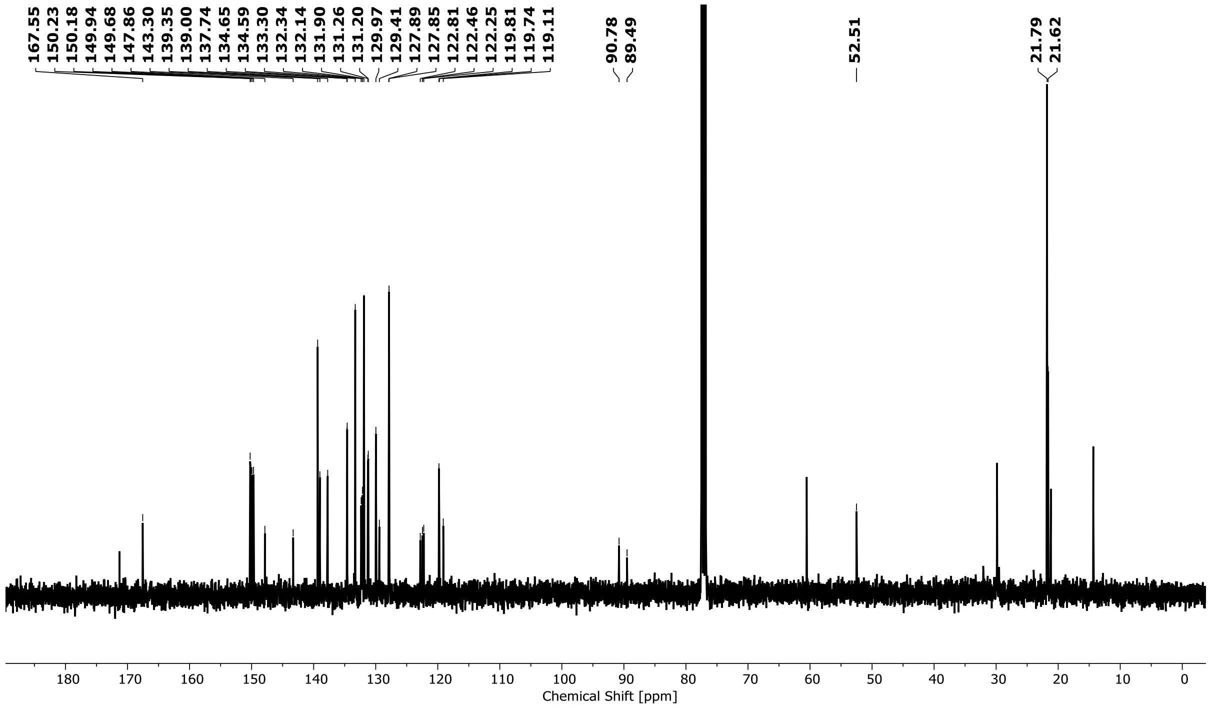

Figure S24 ^{13}C NMR (101 MHz, $CDCl_3$, rt) of **10**

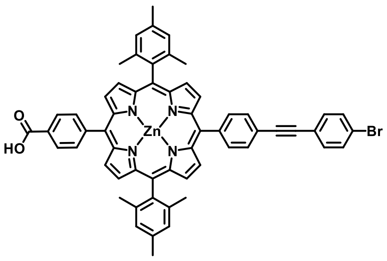

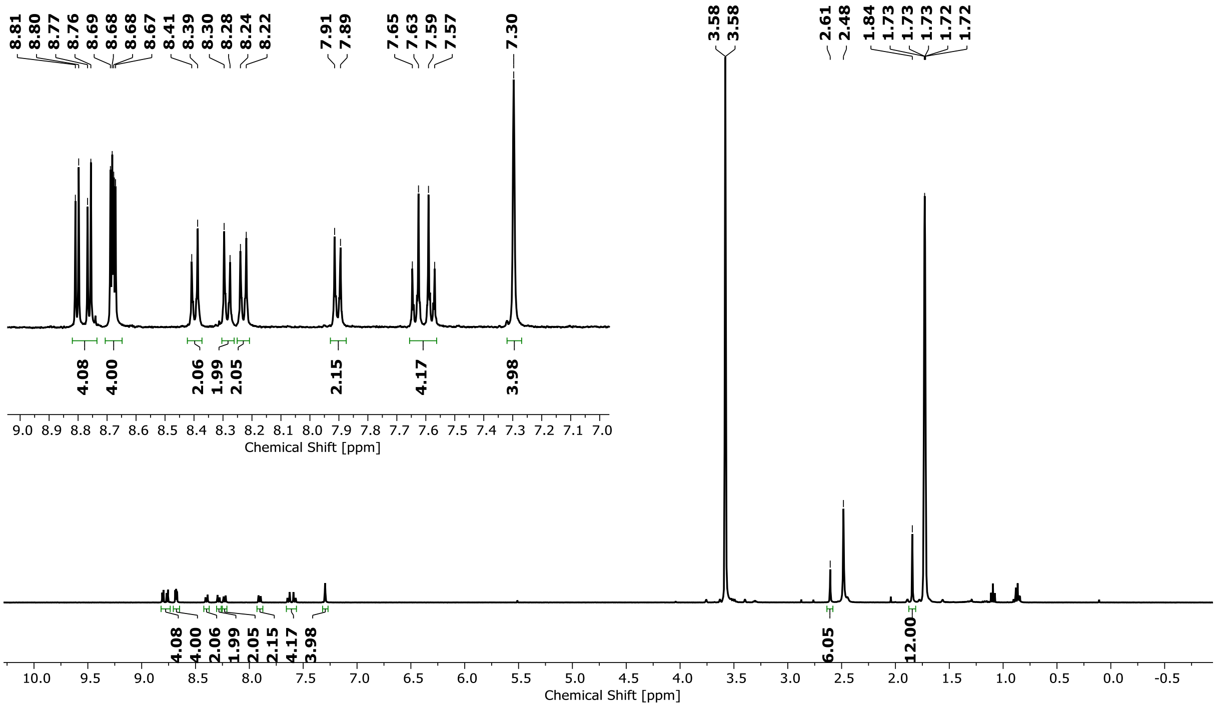

Figure S25 ^{1}H NMR (400 MHz, THF-d_8, rt) of **11**

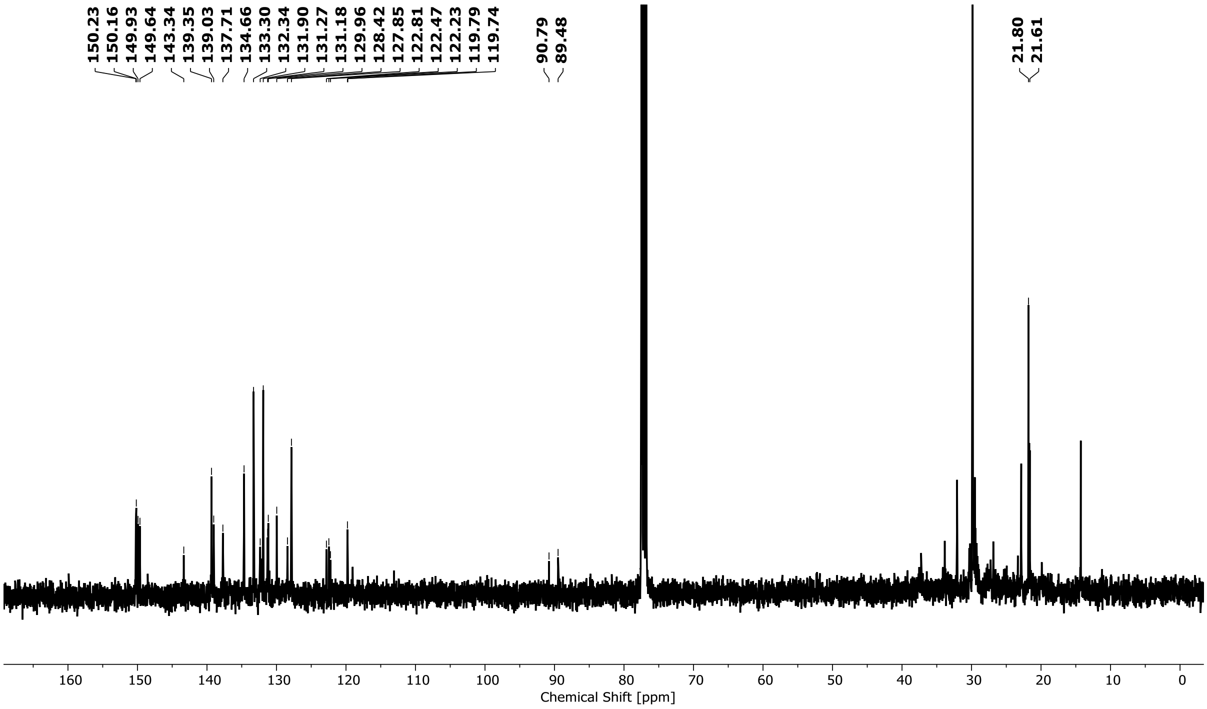

Figure S26 ^{13}C NMR (101 MHz, $CDCl_3$, rt) of **11**

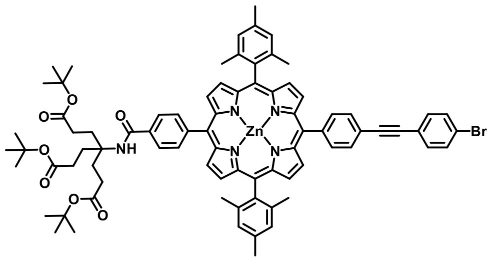

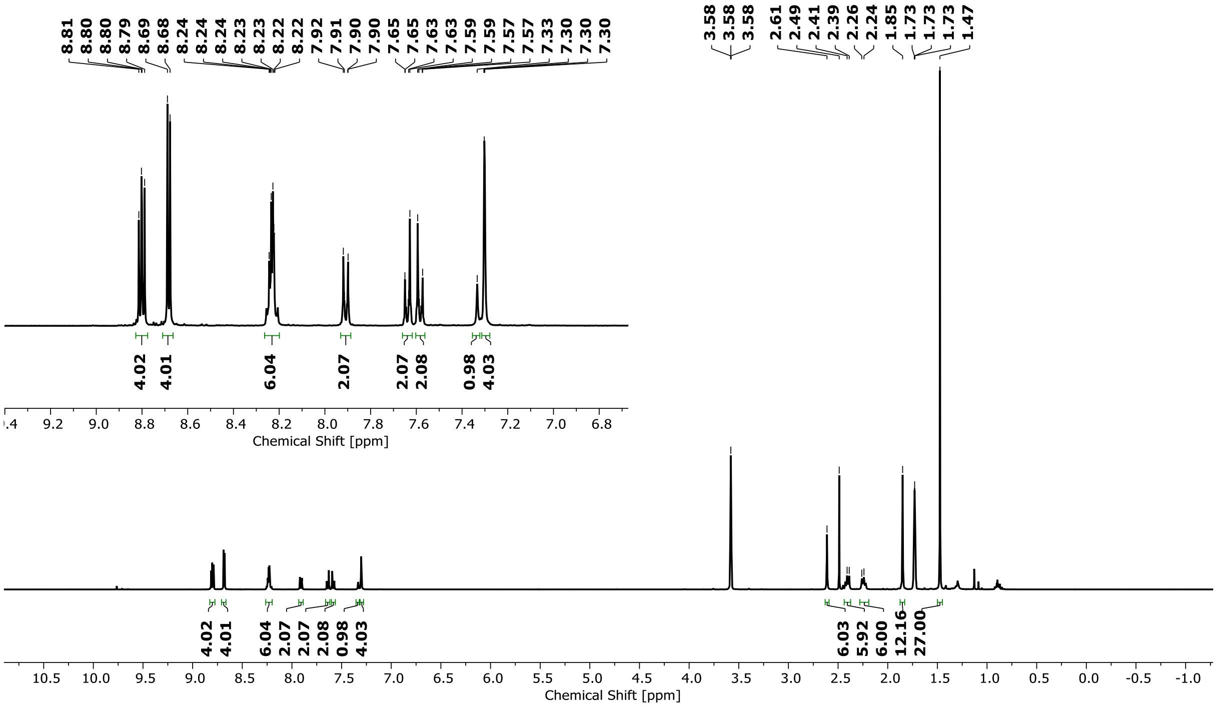

Figure S27 ^{1}H NMR (400 MHz, THF-d_8, rt) of **12**

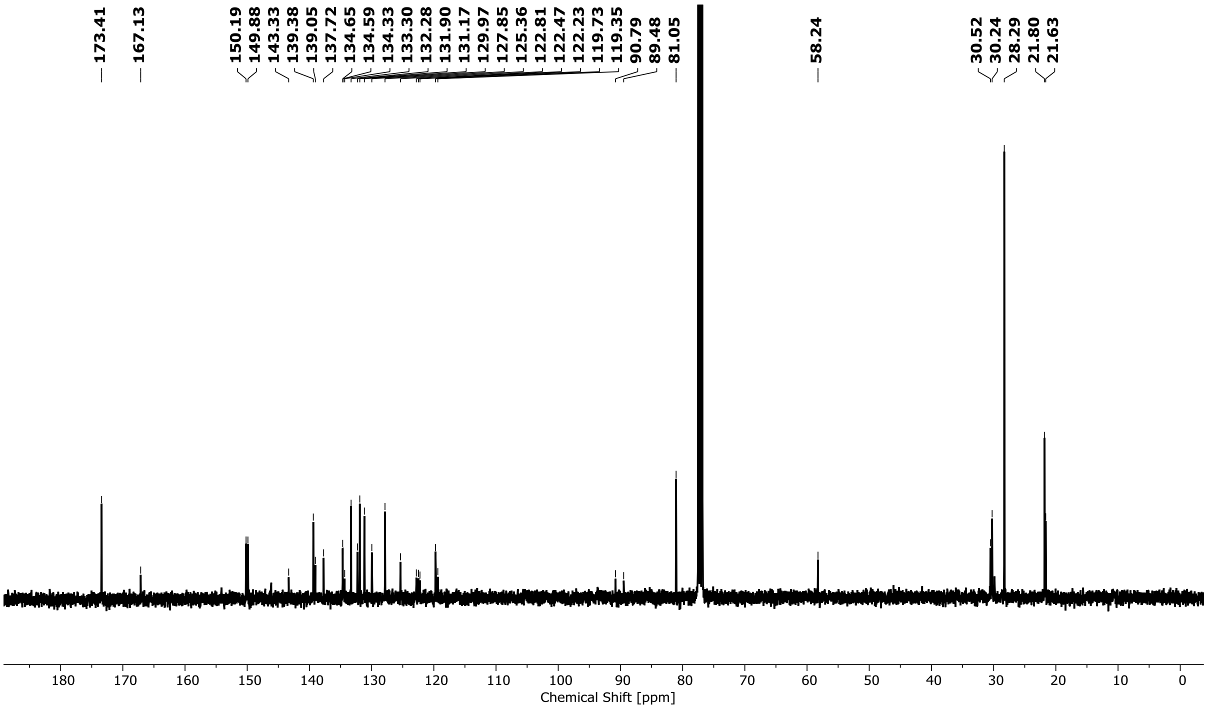

Figure S28 ^{13}C NMR (101 MHz, $CDCl_3$, rt) of **12**

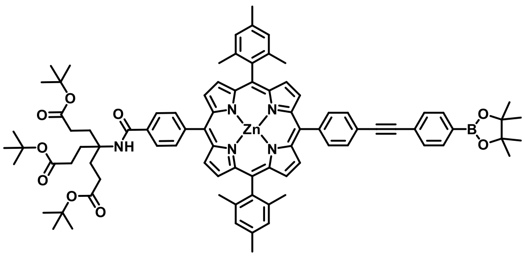

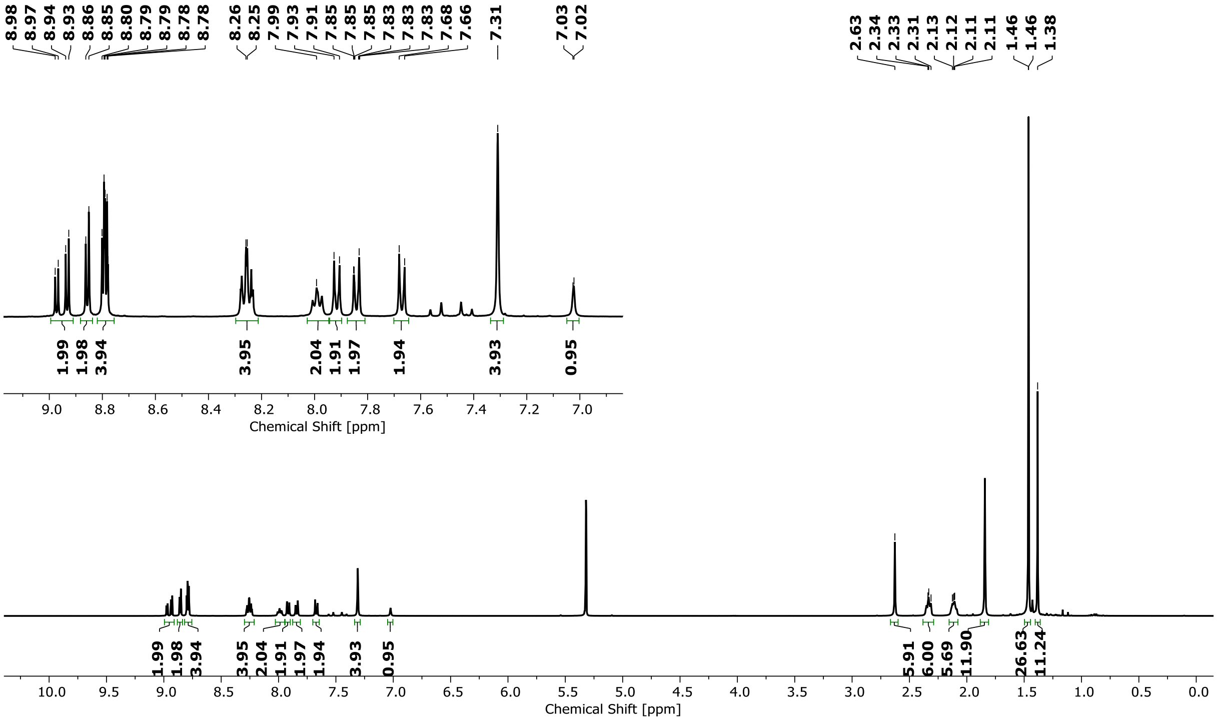

Figure S29 ^{1}H NMR (400 MHz, CD_2Cl_2, rt) of **13**

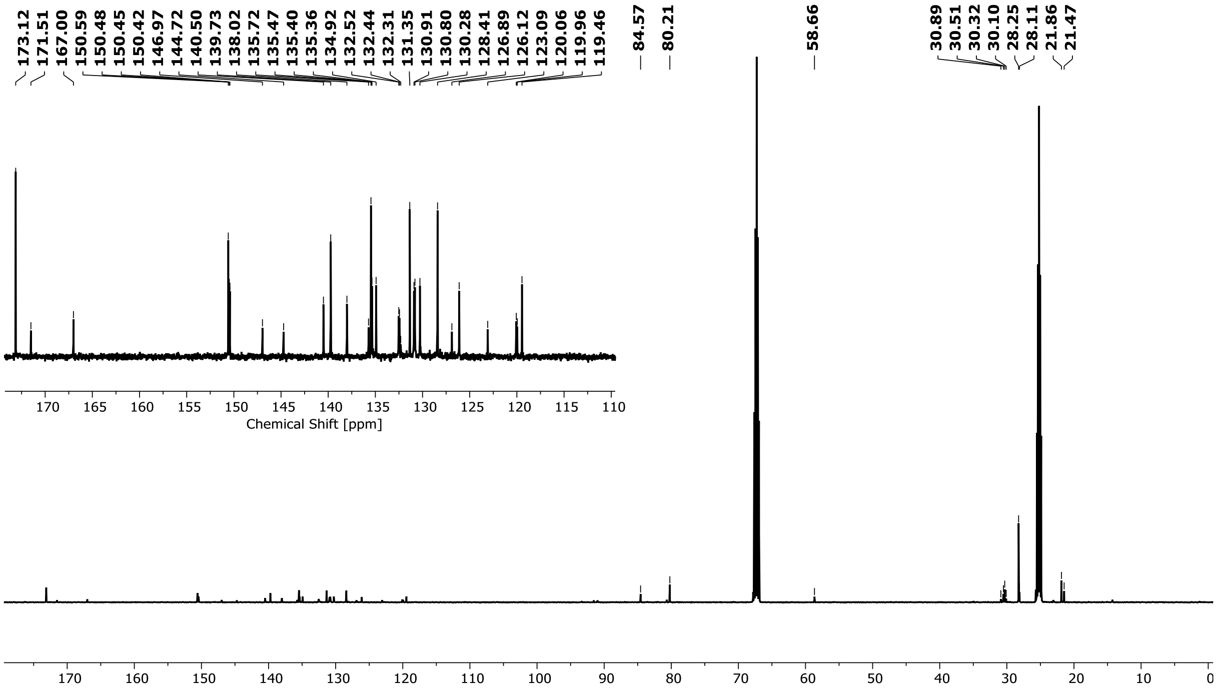

Figure S30 ^{13}C NMR (101 MHz, THF-d_8, rt) of **13**

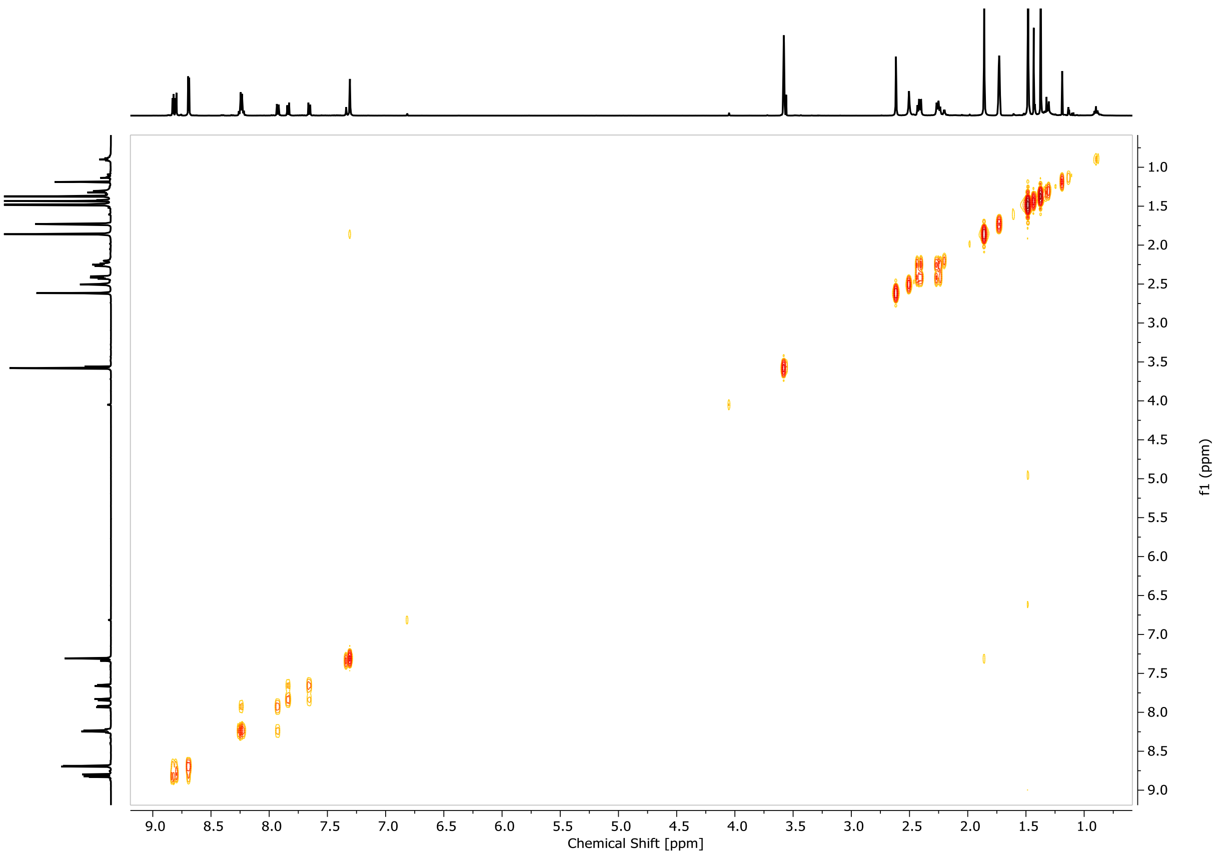

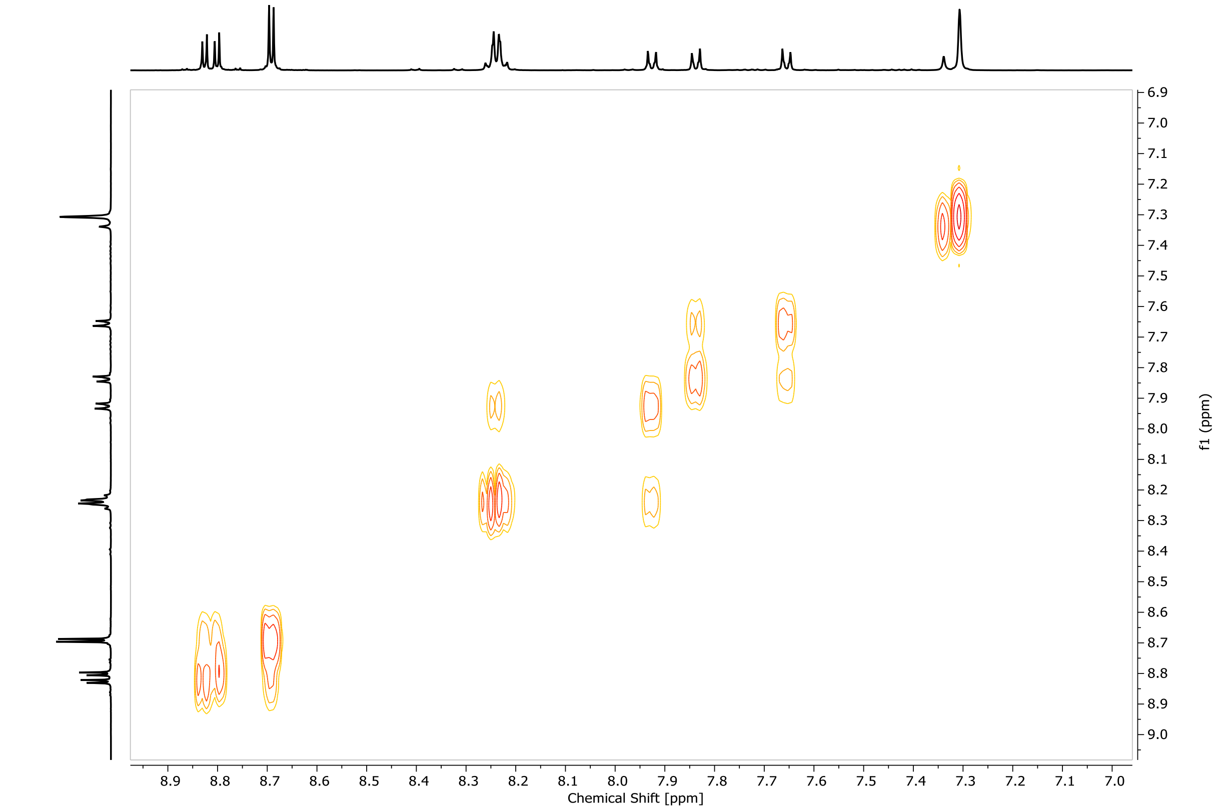

Figure S31 COSY NMR of **13**

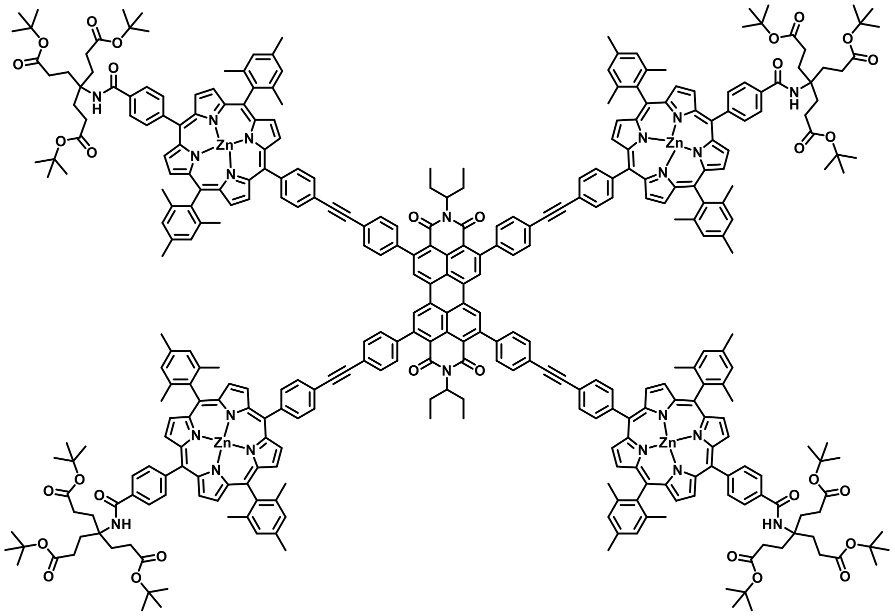

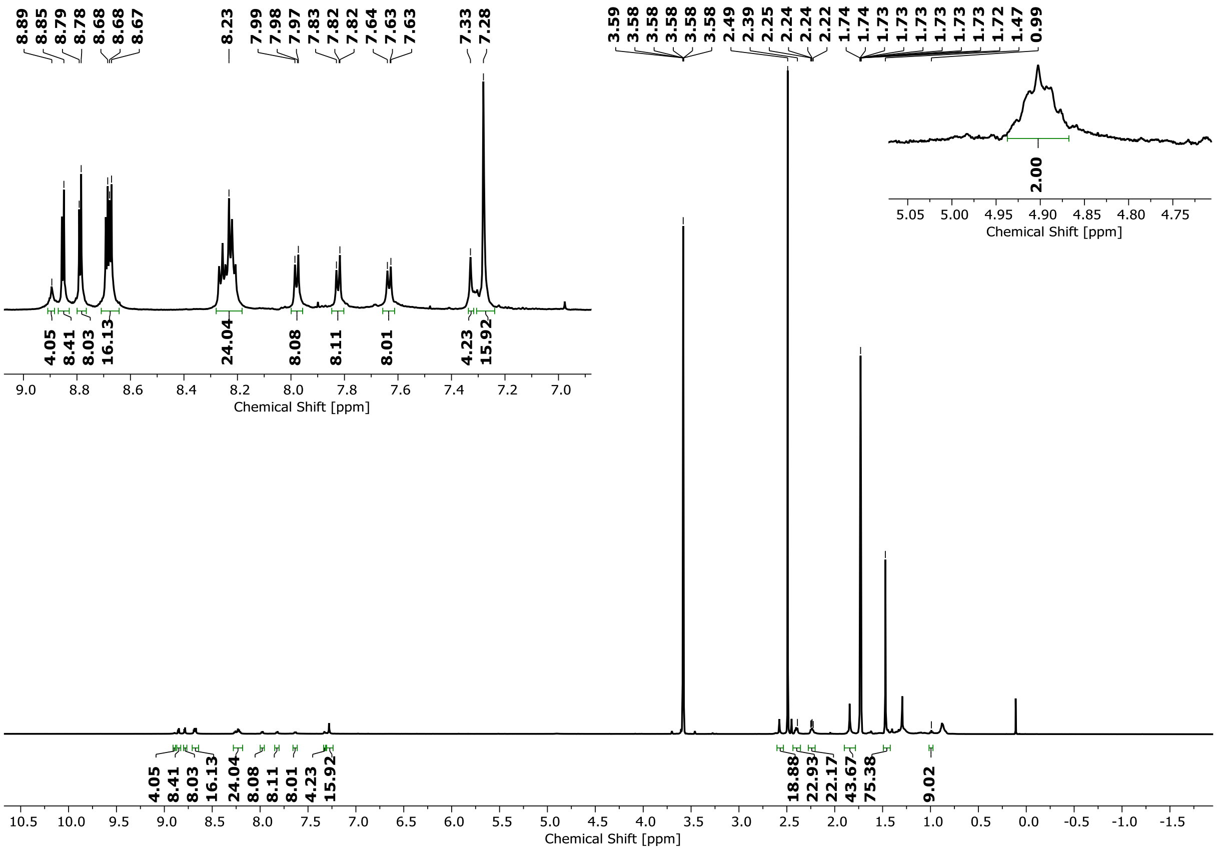

Figure S32 ^{1}H NMR (600 MHz, THF-d_8, rt) of **14** (*Despite our best efforts, due to the low concentration of the sample some signals are very weak and consequently strongly influenced by the baseline noise. Therefore, some relative integrals show some discrepancies to the expected values)*.

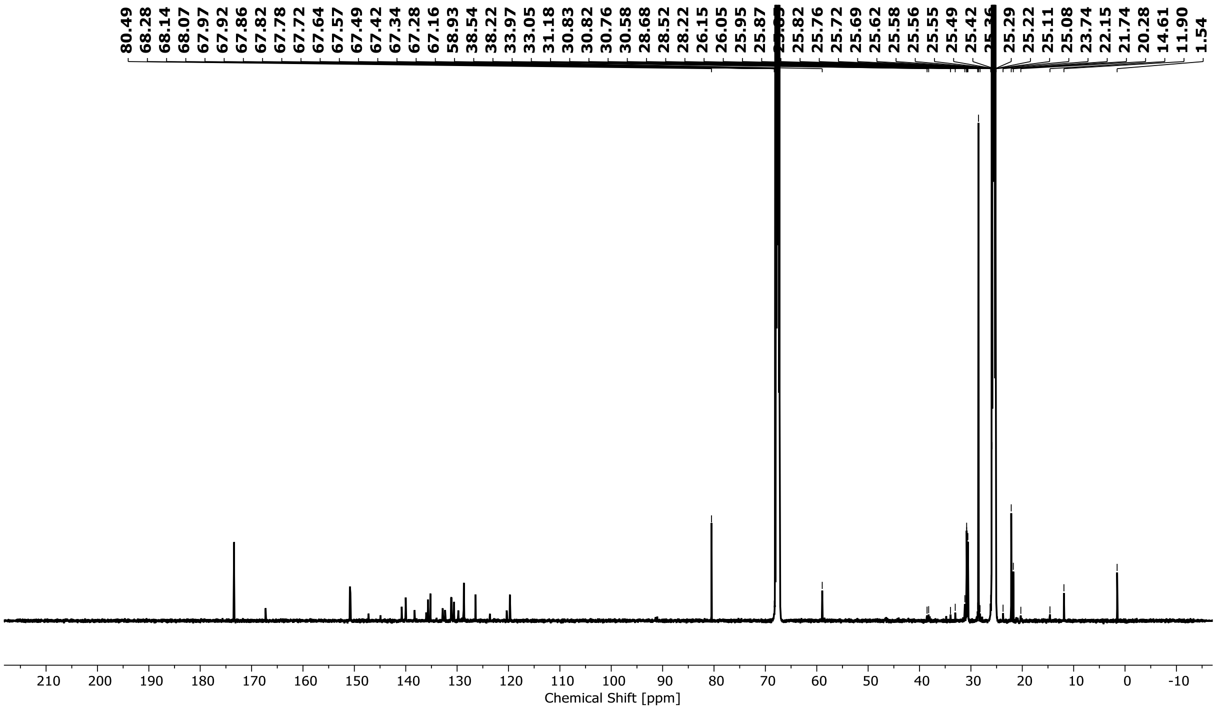

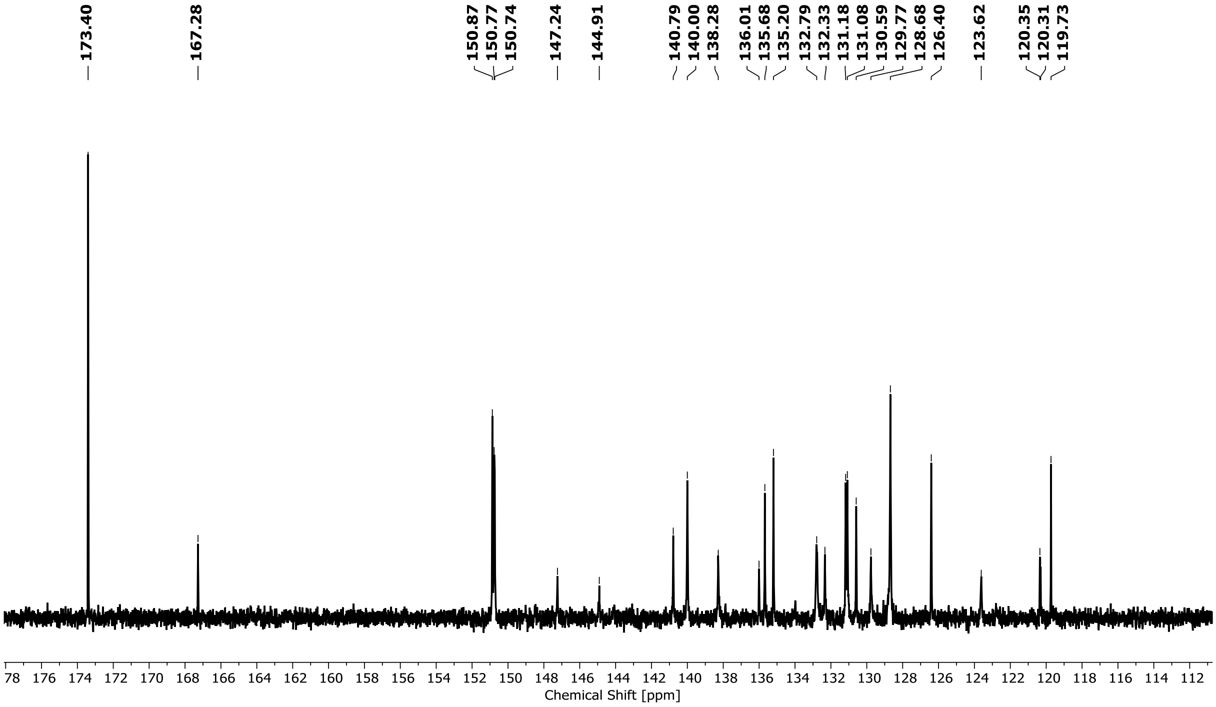

Figure S33 ^{13}C NMR (151 MHz, THF-d_8, rt) of **14**

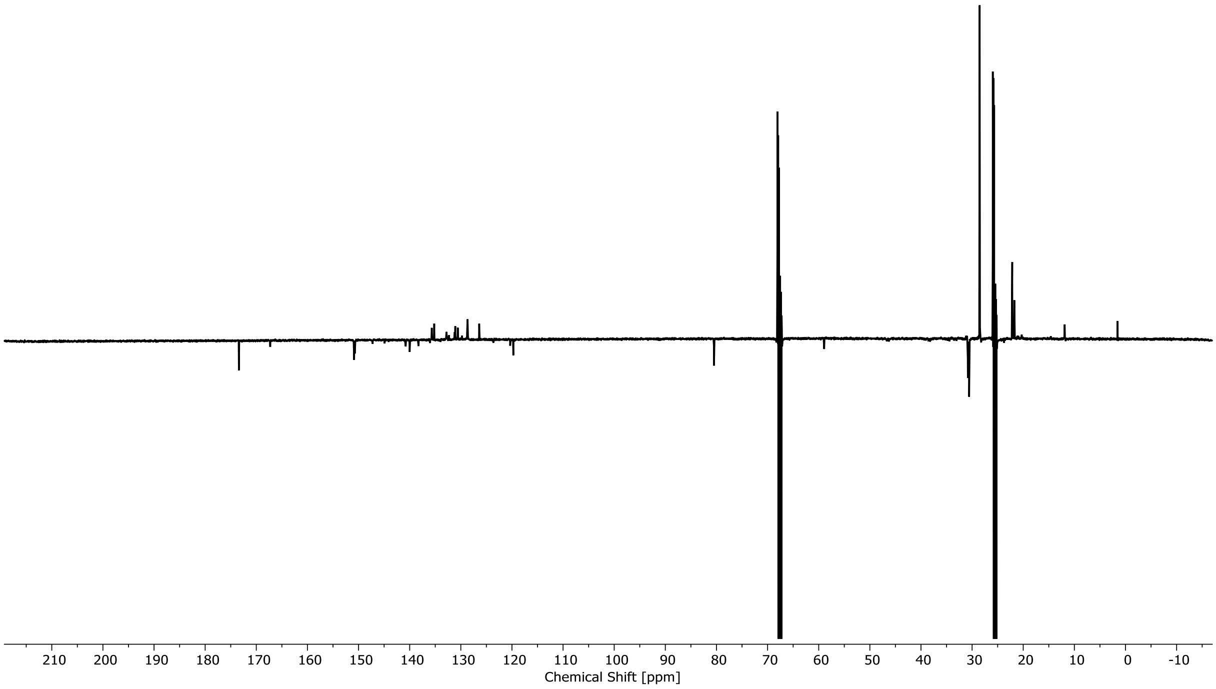

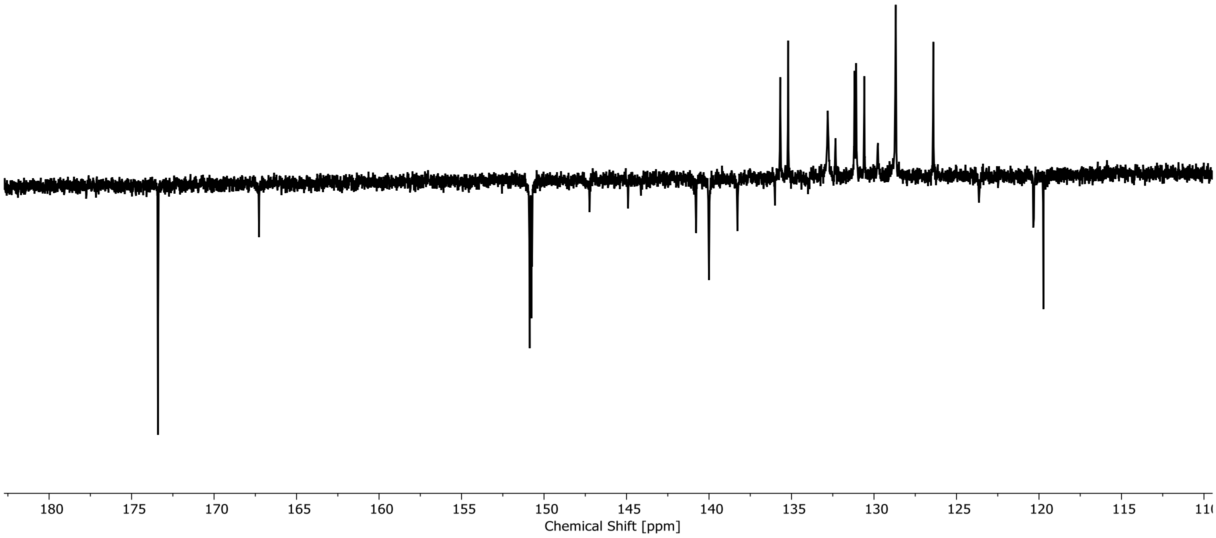

Figure S34 DEPTq-135 NMR of **14**

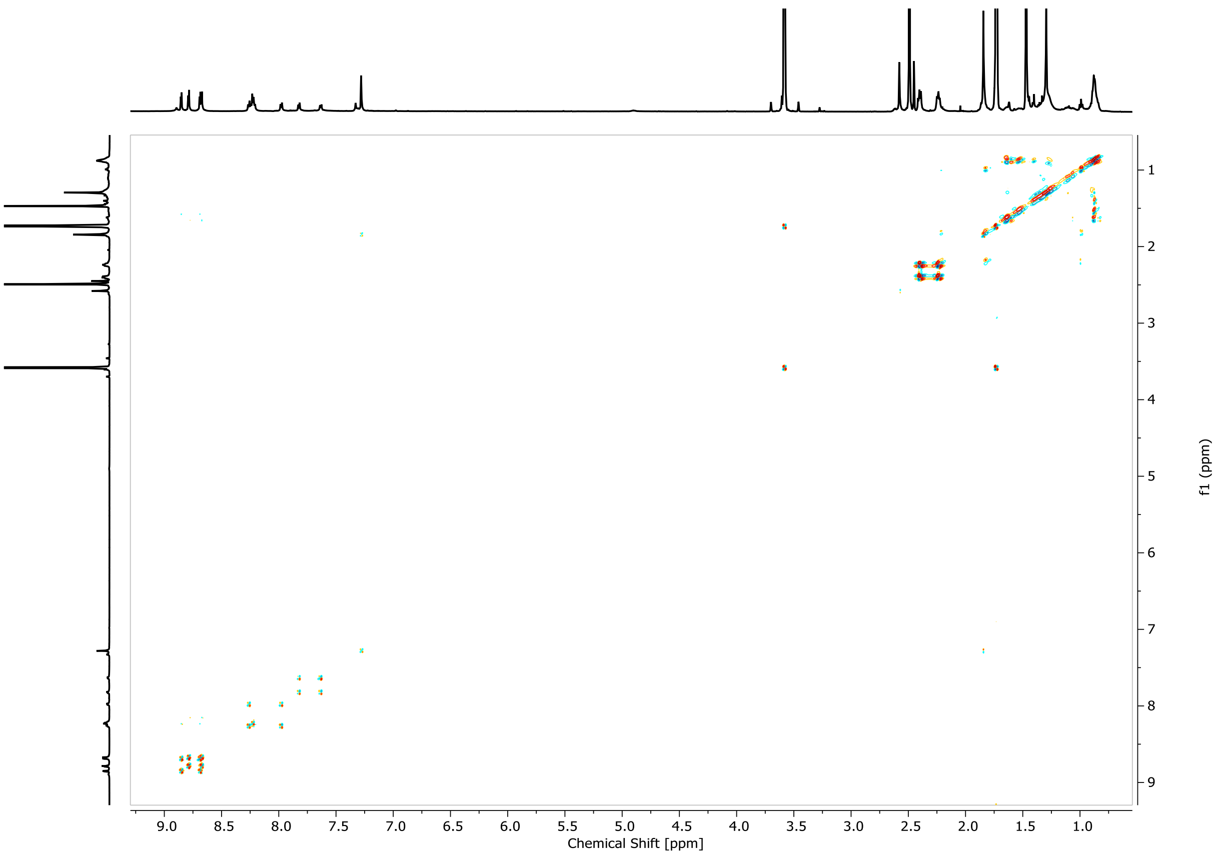

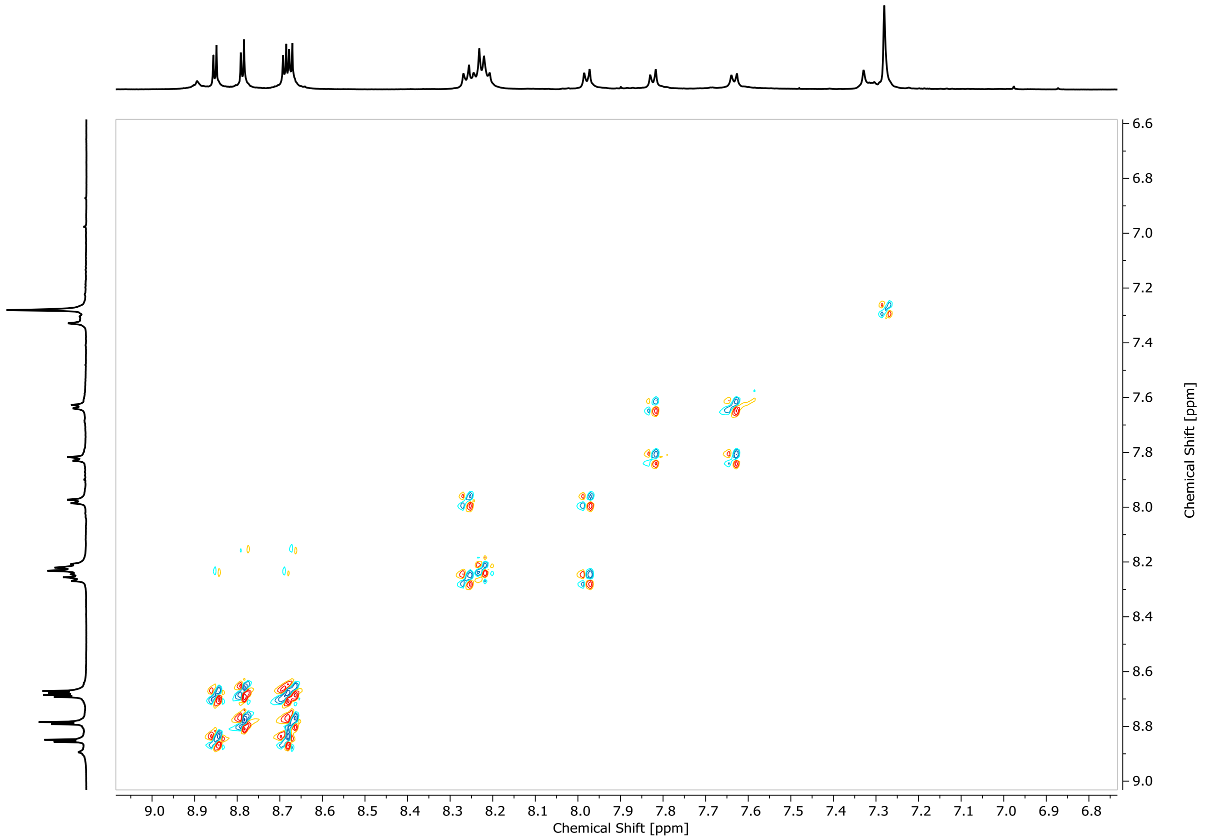

Figure S35 COSY NMR of **14**

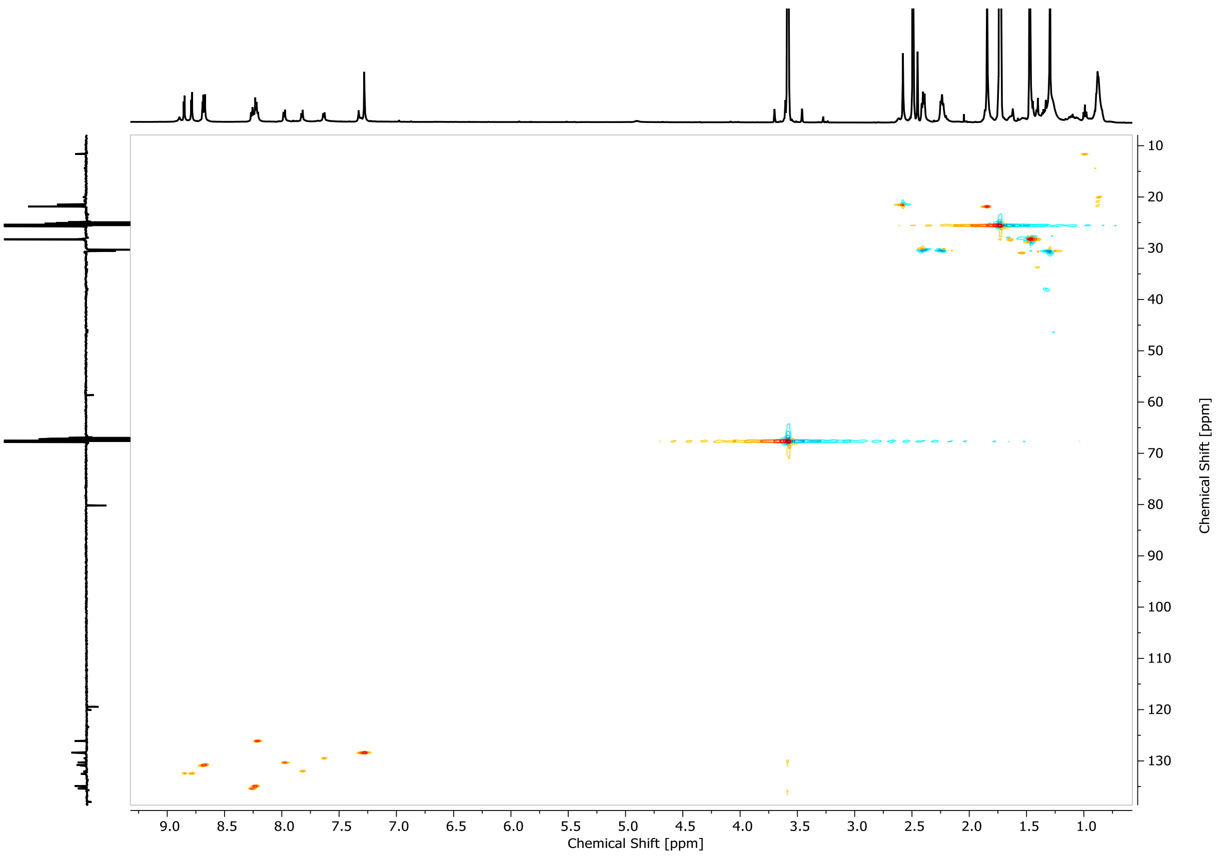

Figure S36 HSQC NMR of **14**

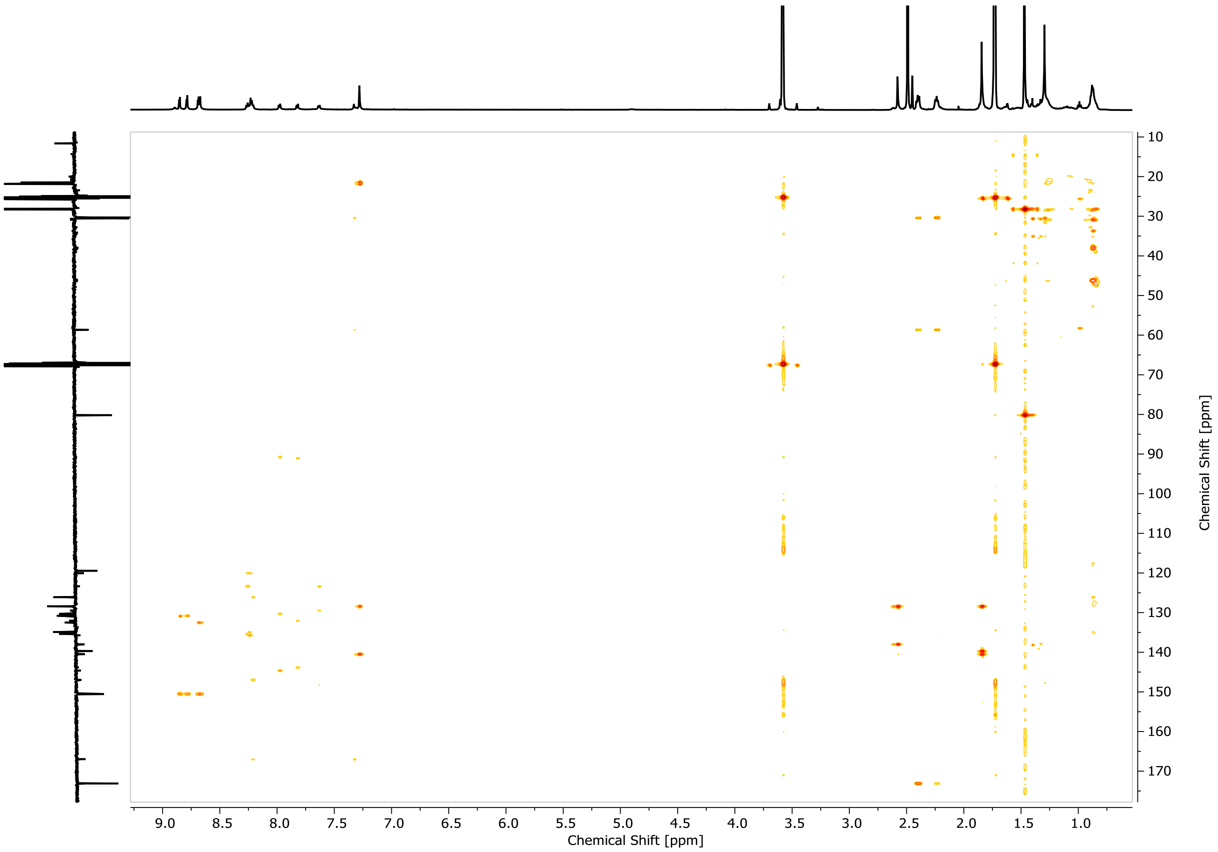

Figure S37 HMBC NMR of **14**

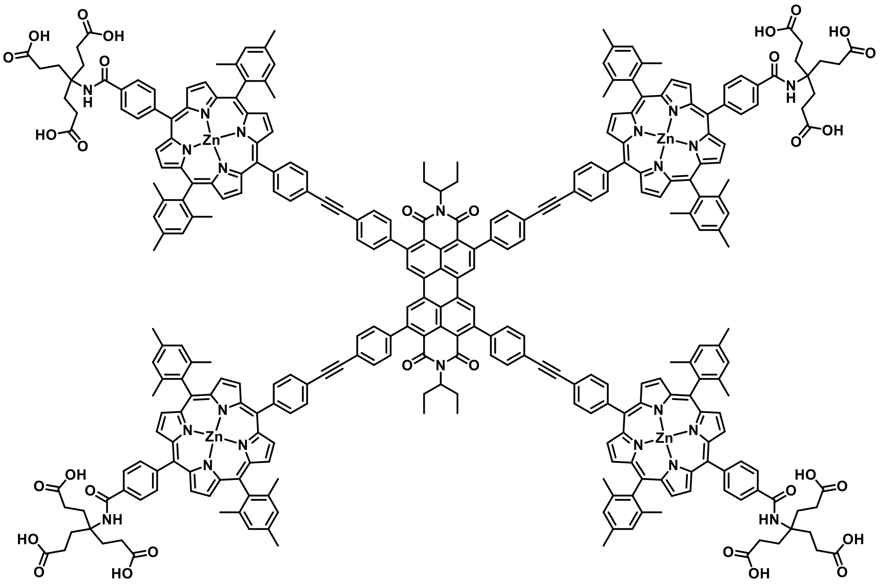

*Acquisition of a well resolved ^{1}H NMR spectrum of **15** proved difficult due to the high molecular weight of the compound, as well as the limited amount of substance we were able to obtain. Furthermore, longer measurement times were not possible as under the conditions required for the NMR (TFA acidified THF solutions) demetalation takes place. Consequently, only very low intensity spectra were obtained, where the baseline also influences the integrals, which is why integration of the signals do not exactly match the theoretical expected ones.*

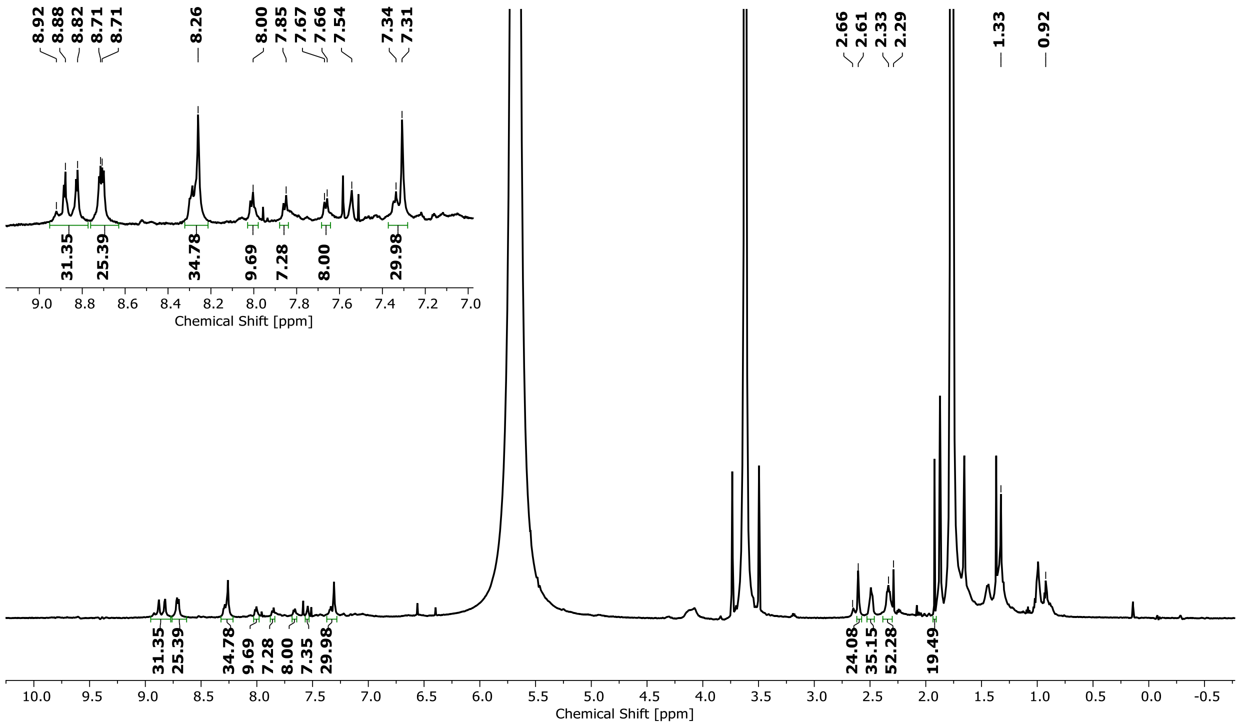

Figure S38 ^{1}H NMR (600 MHz, THF-d_8 + 1 vol% TFA-d, rt) of **15**

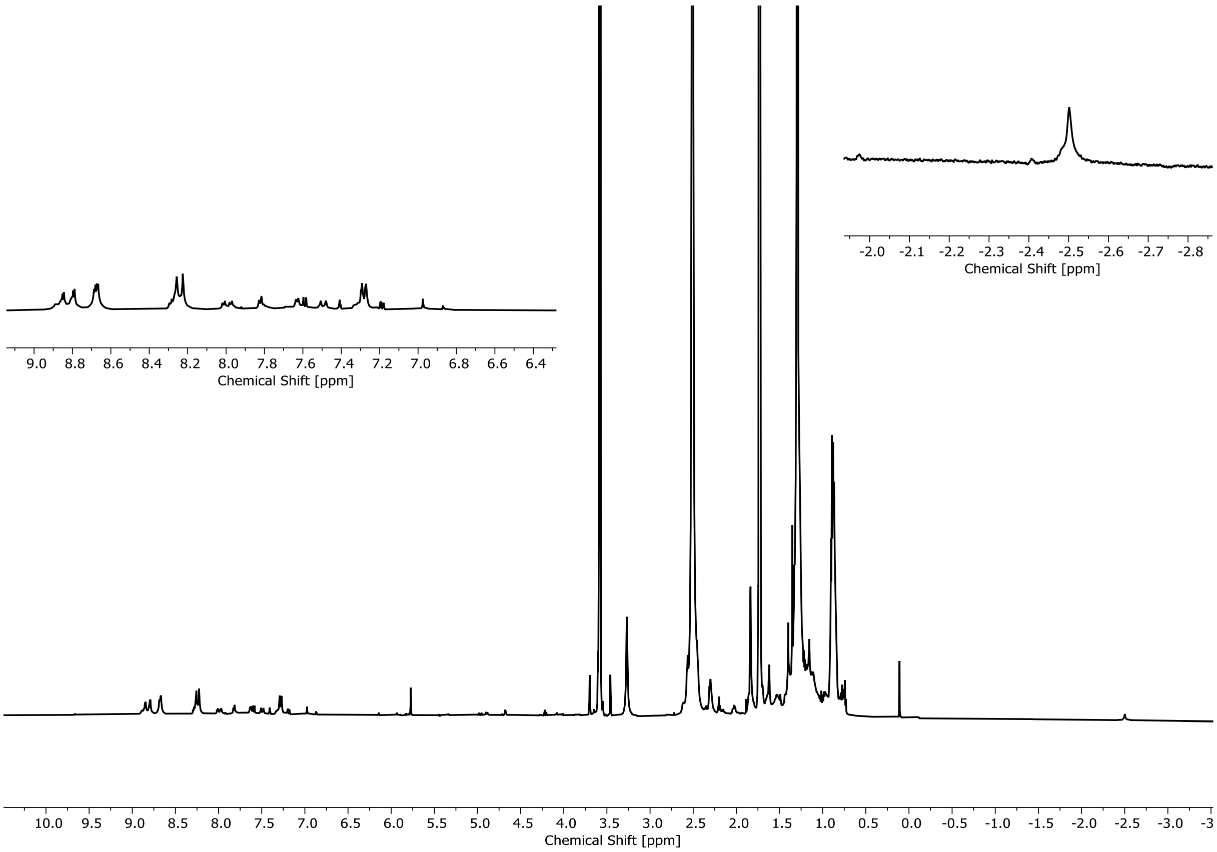

Figure S39 ^{1}H NMR (600 MHz, THF-d_8, rt) of **15** after acidification with TFA-d, showing partial demetallation over the acquisition of the corresponding ^{13}C NMR

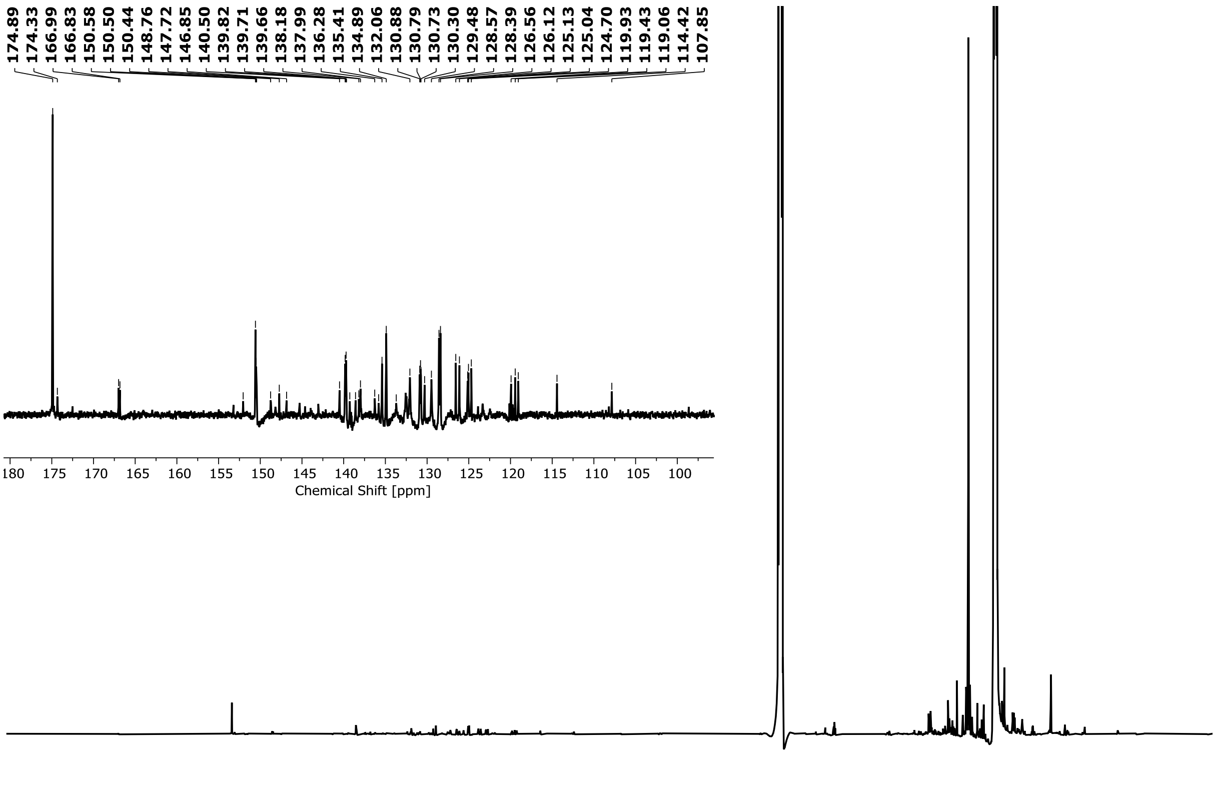

Figure S40 ^{13}C NMR (151 MHz, THF-d_8, rt) of **15** after acidification with TFA-d, showing partial demetallation

3. UV/Vis / Fluorescence Spectra

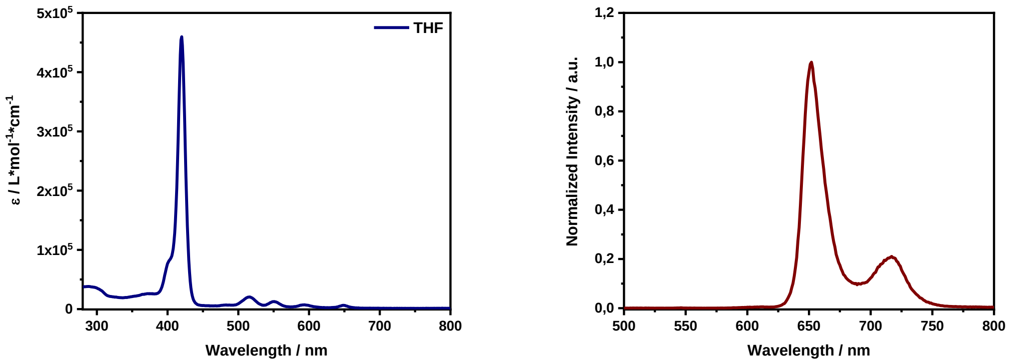

Figure S41 Left: Absorption Spectrum of **1**; Right: Normalized emission spectrum of **1** excited at 420 nm at room temperature

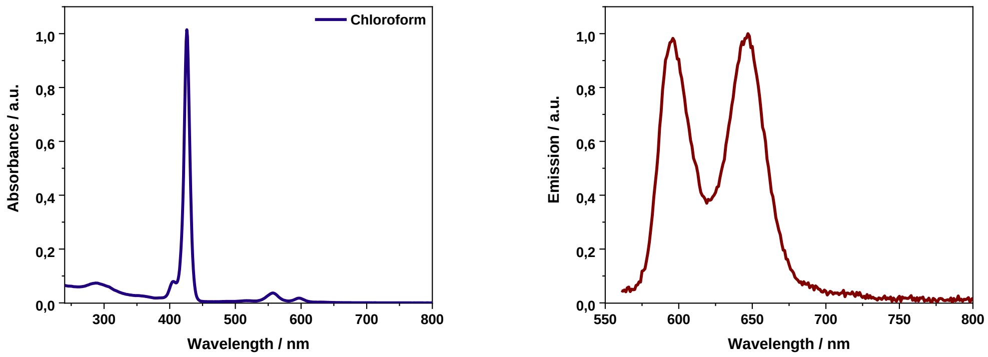

Figure S42 Left: Absorption Spectrum of **2**; Right: Normalized emission spectrum of **2** excited at 420 nm at room temperature

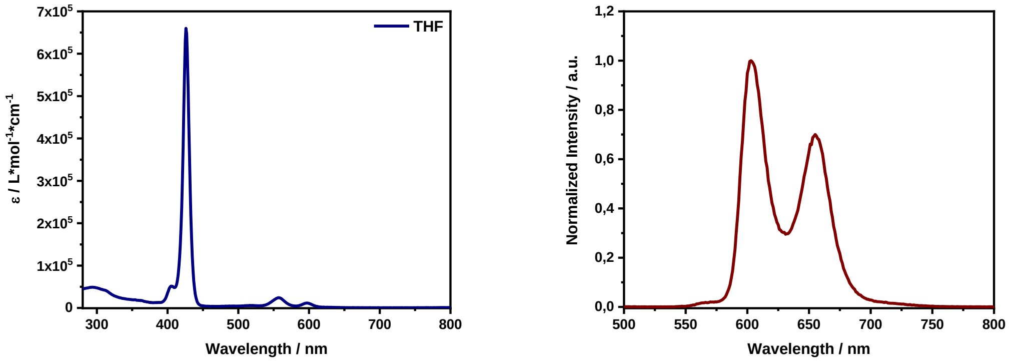

Figure S43 Left: Absorption Spectrum of **3**; Right: Normalized emission spectrum of **13** excited at 420 nm at room temperature

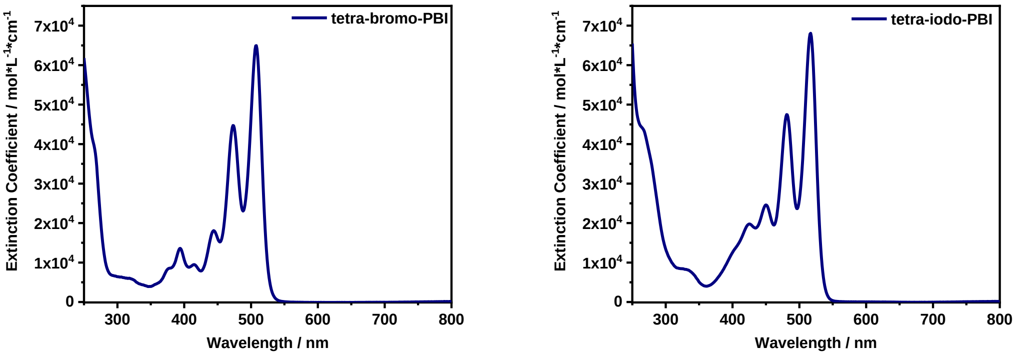

Figure S44 Left: Absorption Spectrum of **6** in THF; Right: Absorption spectrum of **7** in THF

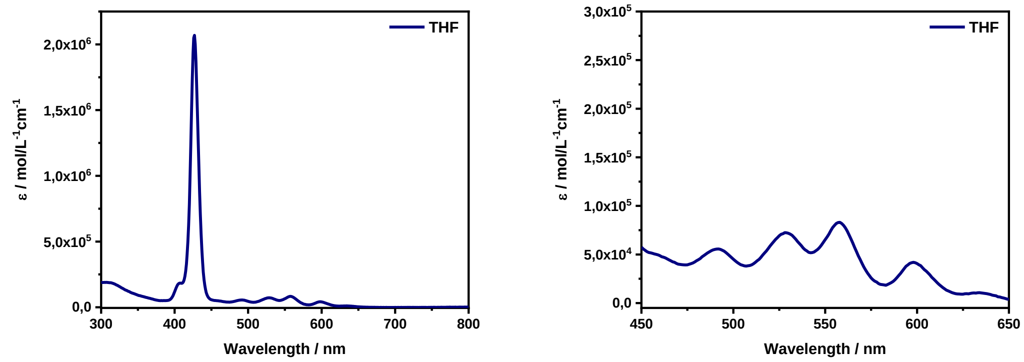

Figure S45 Left: Absorption Spectrum of **8**; Right: partial spectrum of the Q-band and PBI bands

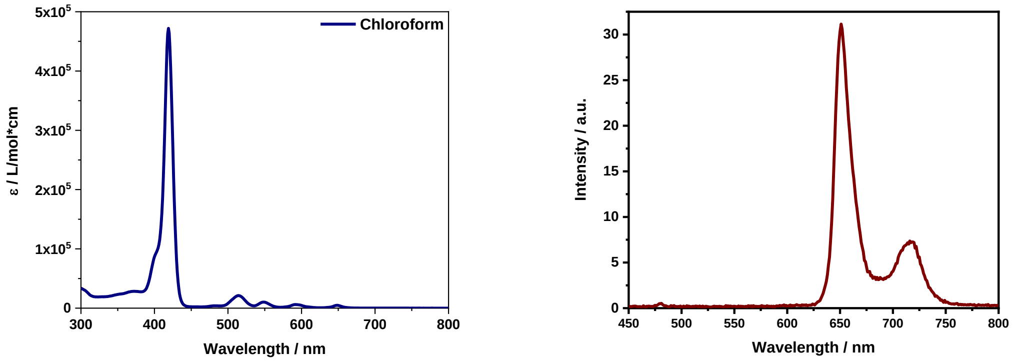

Figure S46 Left: Absorption Spectrum of **9**; Right: Emission spectrum of **9** excited at 420 nm at room temperature

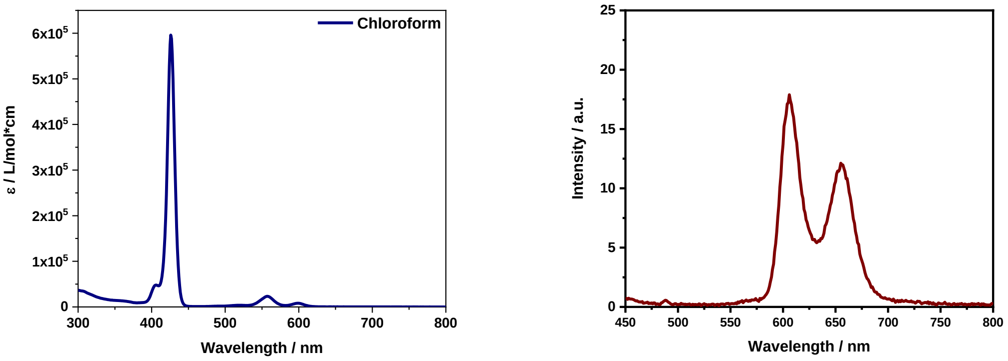

Figure S47 Left: Absorption Spectrum of **10**; Right: Emission spectrum of **10** excited at 425 nm at room temperature

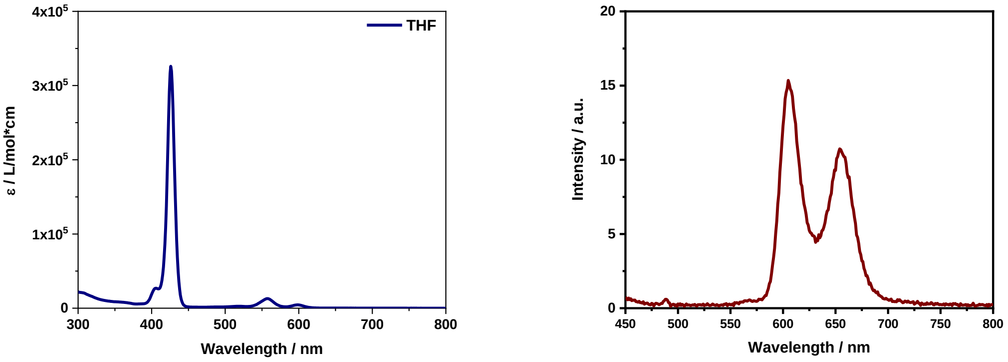

Figure S48 Left: Absorption Spectrum of **11**; Right: Emission spectrum of **11** excited at 425 nm at room temperature

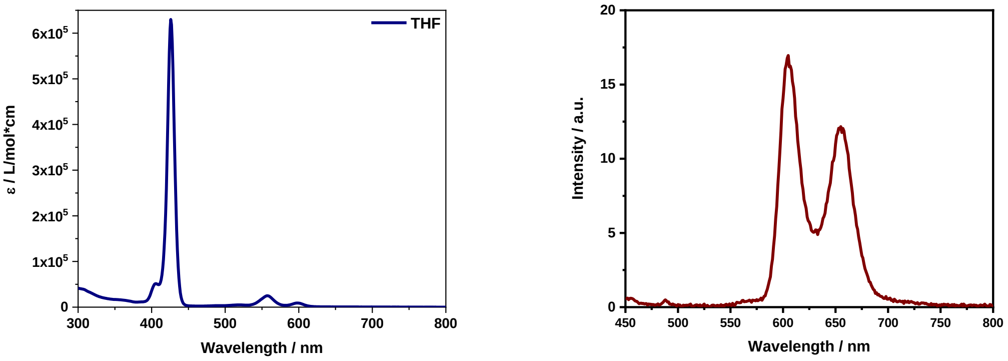

Figure S49 Left: Absorption Spectrum of **12**; Right: Emission spectrum of **12** excited at 425 nm at room temperature

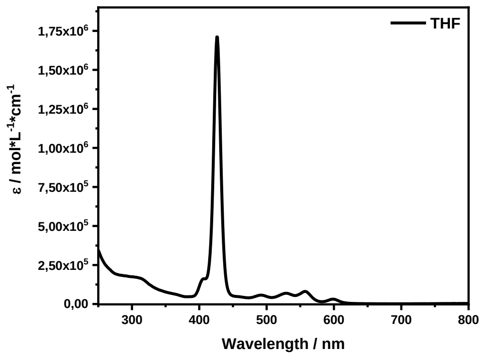

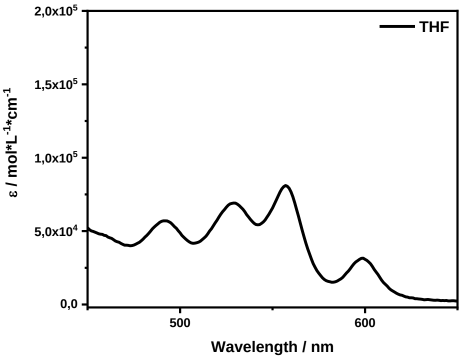

Figure S50 Absorption spectrum of **14** in THF; right: partial spectrum of the Q-band and PBI bands

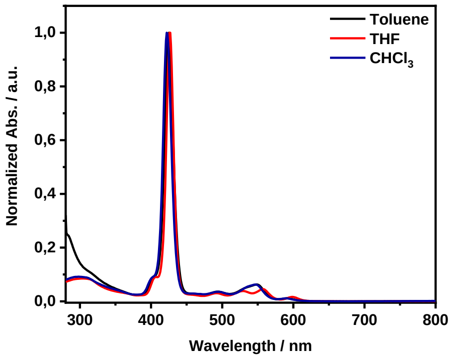

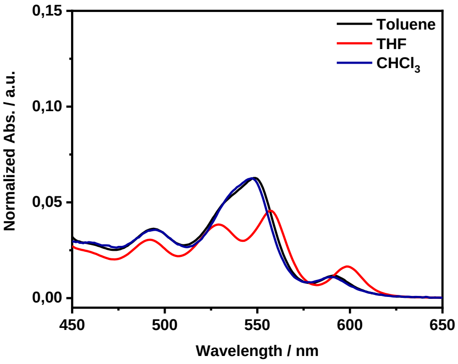

Figure S51 Absorption spectra of **14** in different solvents, as indicated; right: partial spectrum of the Q-band and PBI bands

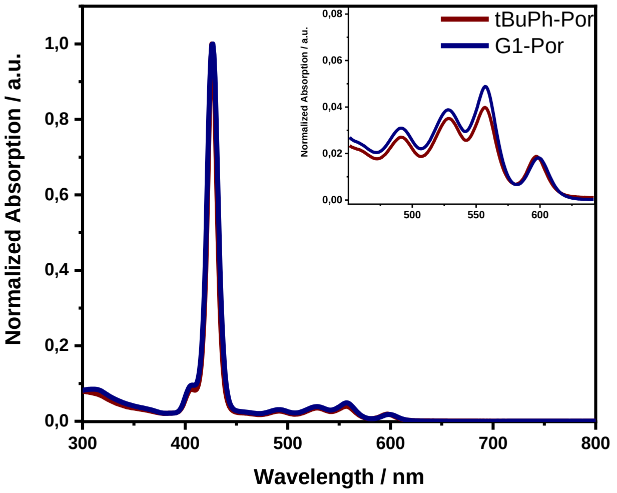

Figure S52 Comparison of the normalized absorption spectra of **8** and **14** in THF

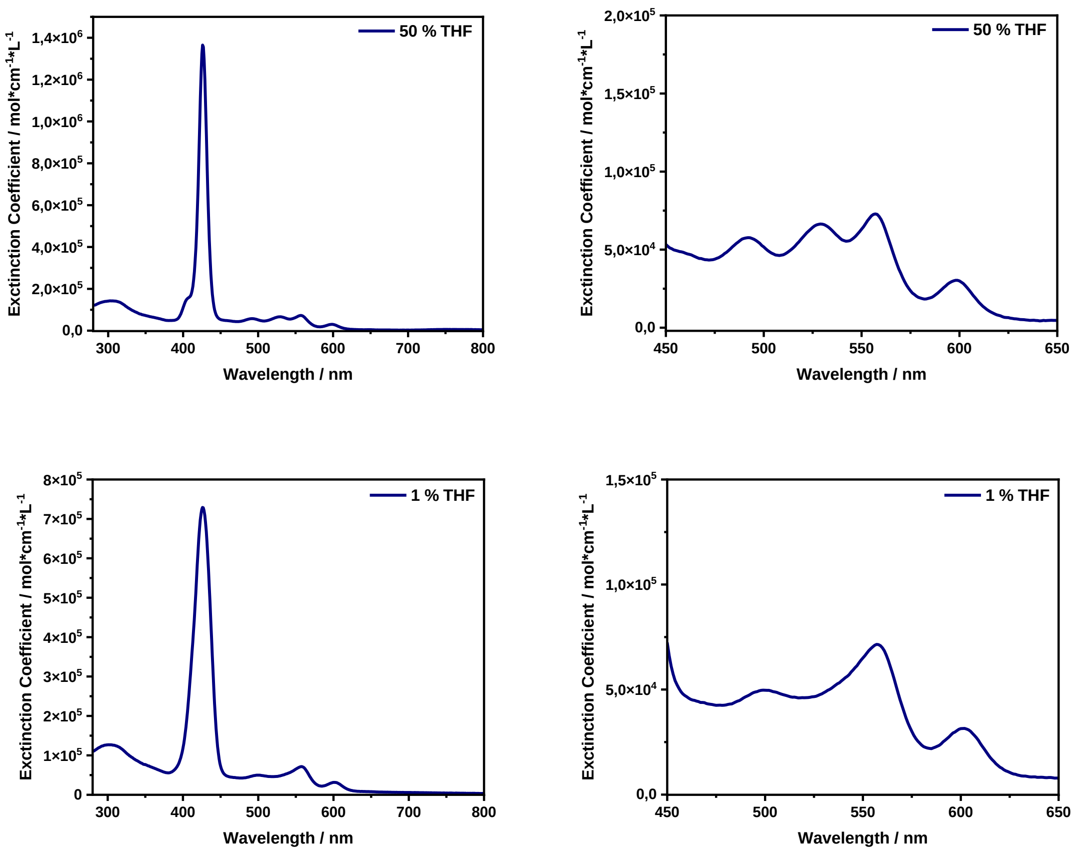

Figure S53 Normalized absorption spectrum of **15** in THF / 10 mM aqueous NaOH solutions (parts as indicated); right: partial spectrum of the Q-band and PBI bands

4. UV/Vis Aggregation Studies

Amphiphile **15** was dissolved in a freshly prepared solution of 10 mM NaOH in water (Milli-Q®) and 10 vol% THF. In separate screw-cap vials, each stock-solution was diluted with the corresponding amounts of 10 mM NaOH(aq) and THF (total volume = 2.5 mL) to give the respective mixtures. Each solution was then measured separately after a curing time of 1 min during which the vials were kept tightly sealed to minimize evaporation of the solvent.

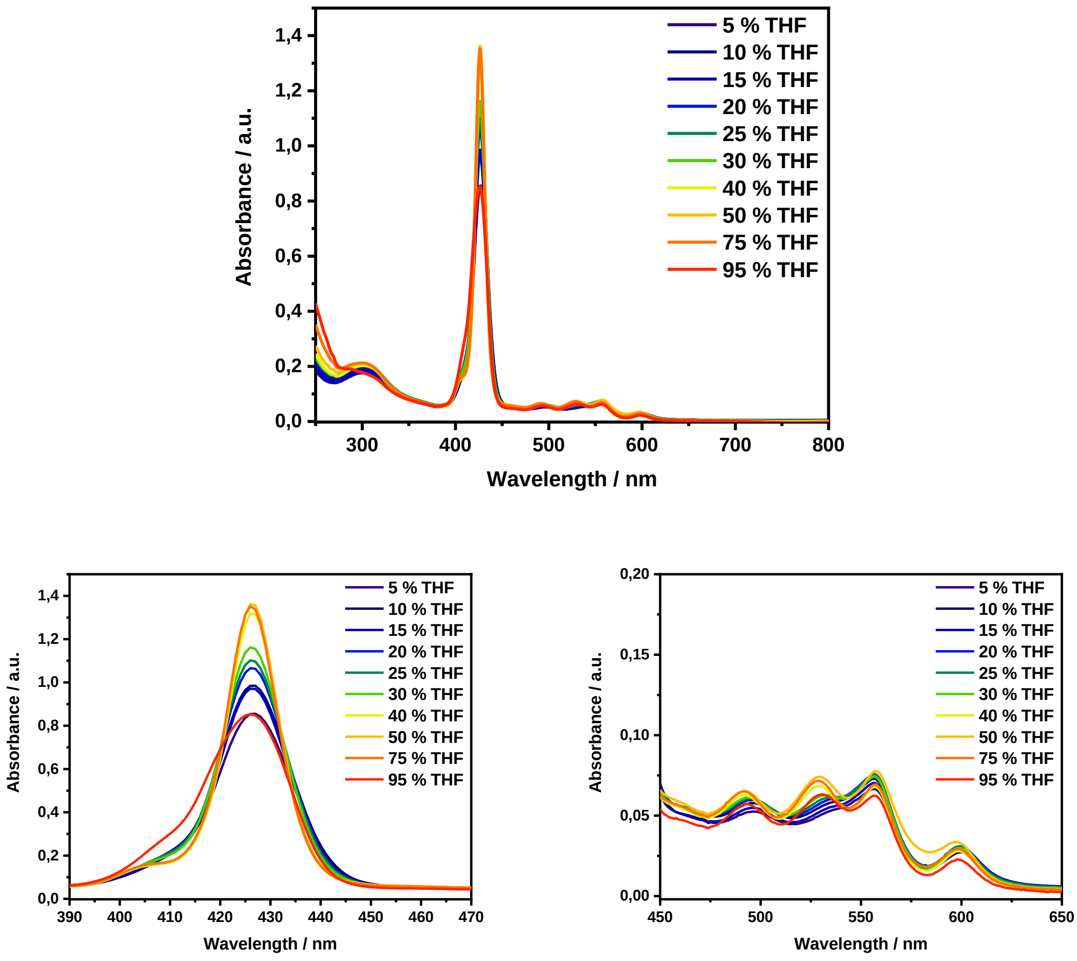

Figure S54 UV/Vis absorption spectra of **15** in 10 mM aqueous NaOH with different parts of THF as indicated. Higher parts of THF as 75 vol% lead to a re-aggregation of the samples, as the deprotonated pentad is not soluble in THF. Higher parts as 95 vol% THF lead to a precipitation of the compound.

Time dependent UV/Vis absorption spectra were recorded to investigate the presence of thermodynamically more stable aggregates which form over time. For this, a 1*10^{-6} M solution of **15** in 10 mM NaOH / 1 vol% THF was monitored over the time of 20 h.

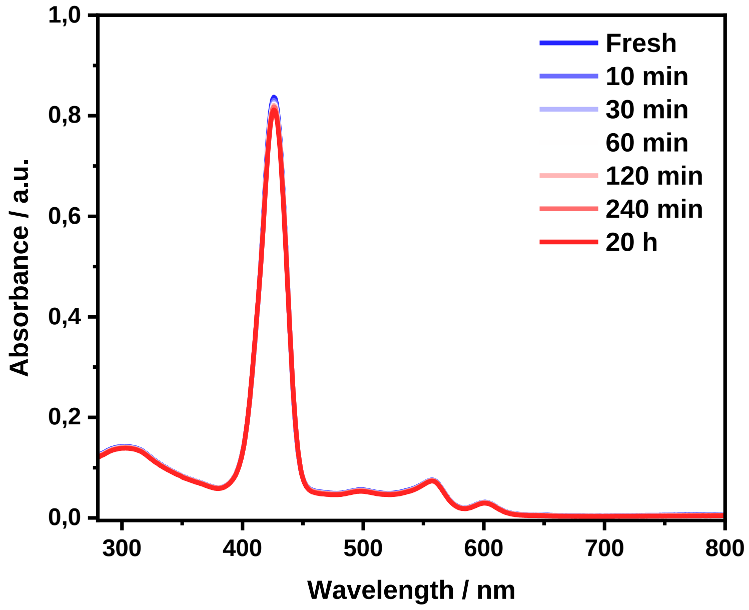

Figure S55 Time dependent UV/vis absorption spectra of **15**

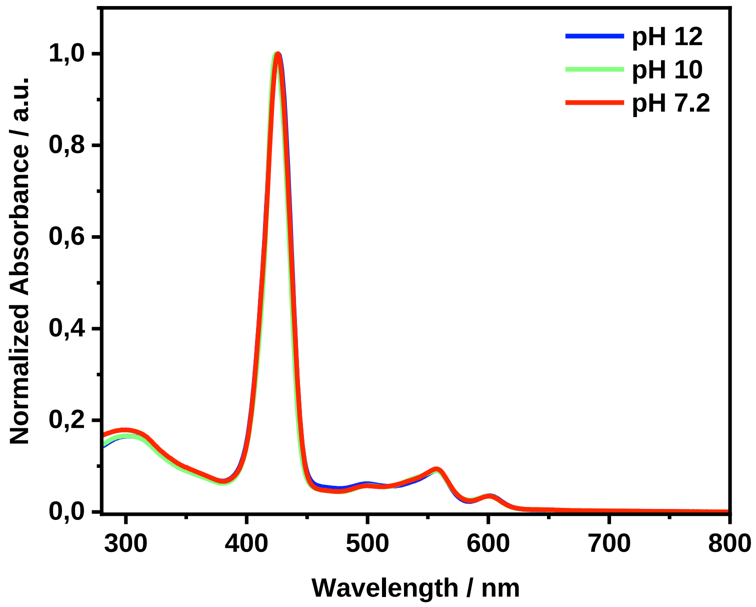

Figure S56 Normalized UV/Vis absorption spectra of **15** at different pH values at c= 1*10^{-6} M (pH 12 = 10 mM NaOH, pH 10 = 0.1 mM NaOH, pH 7.2 Sodium Phosphate Buffer)

Temperature dependent UV/Vis absorption spectra were recorded of a $1*10^{-6}$ M solution of **15** in 10 mM NaOH / 1 vol% THF and in 10 mM NaOH / 5 vol% THF. After reaching the set temperature, the sample was allowed to equilibrate for 5 min while stirring, before measured and the new temperature was set.

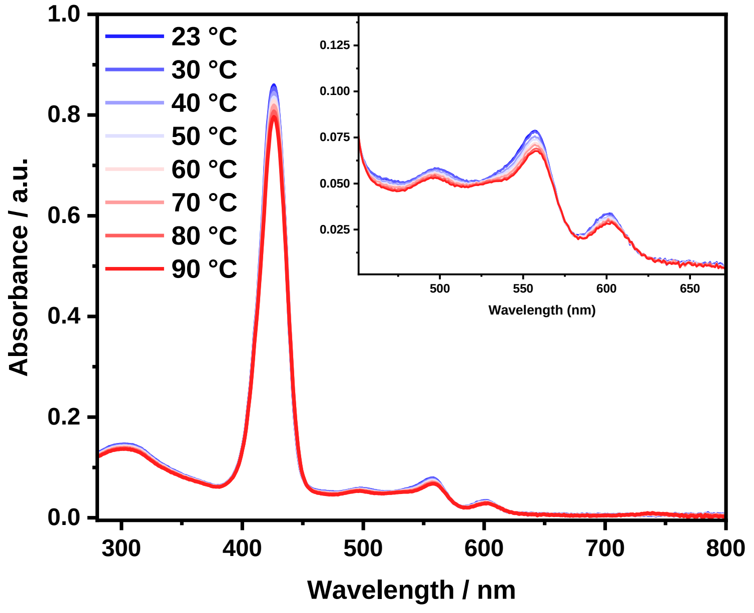

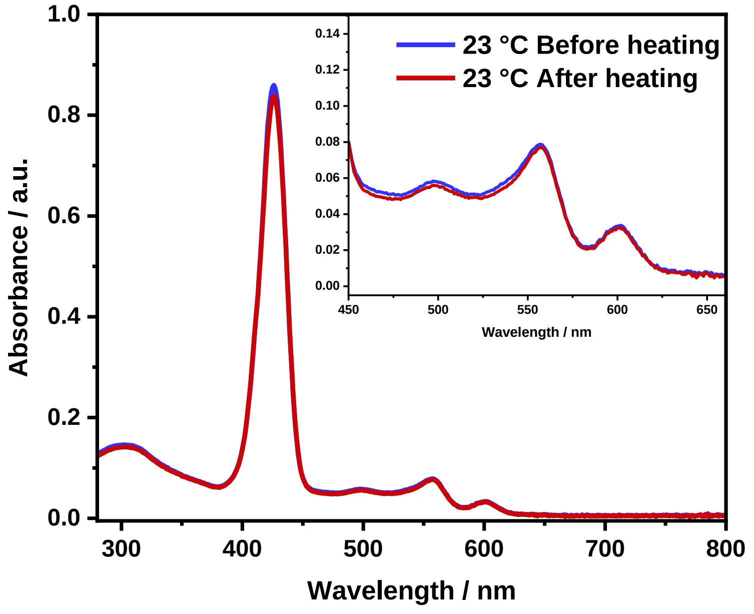

Figure S57 Temperature dependent UV/Vis absorption spectra of 15 in top: 10 mM NaOH / 1 vol% THF and bottom: comparison of the absorption spectrum prior and after the heating at 23 °C

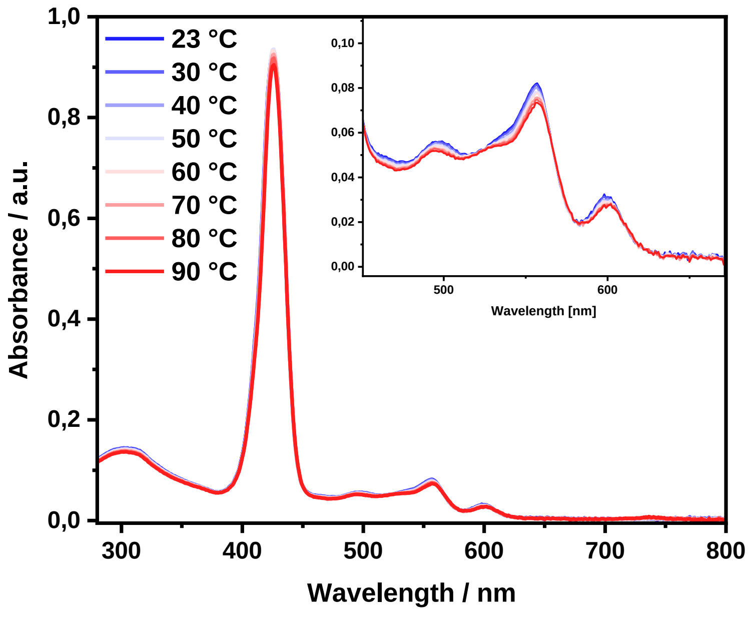

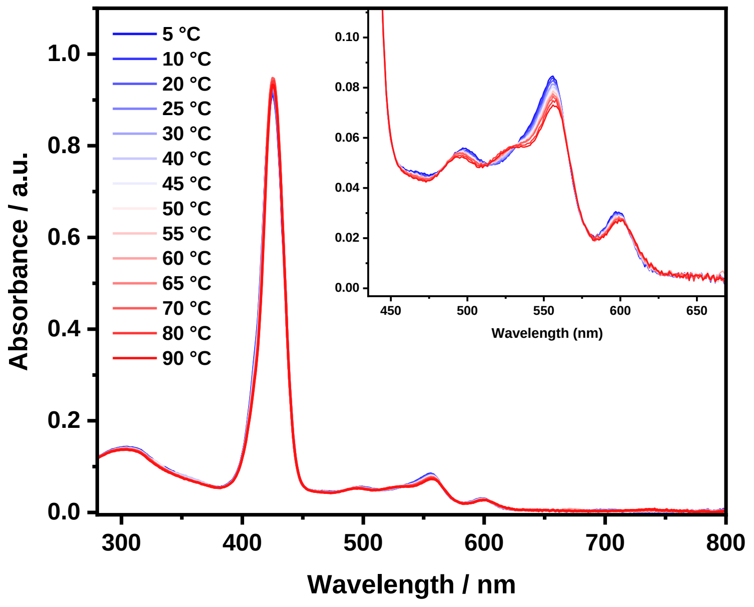

Figure S58 Temperature dependent UV/Vis absorption spectra of 15 in top: 10 mM NaOH / 5 vol% THF and bottom: 10 mM NaOH / 10 vol% THF

5. Dynamic Light Scattering

Dynamic light scattering (DLS) was performed to assess the size of the formed aggregates. Samples were filtered through a micro filter (200 nm) and the average of three measurements is given.

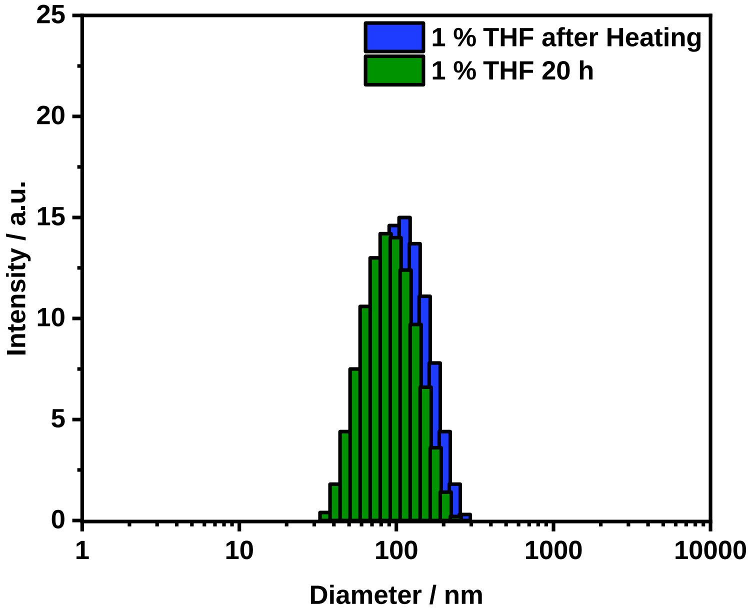

Figure S59 DLS size distribution of **15** recorded in 10 mM NaOH / 1 vol% THF solutions at $1*10^{-6}$ M concentration; blue: sample after the above-mentioned heating cycle, cooled to room temperature; green: sample after 20 h time dependent measurement.

6. Mass Spectra

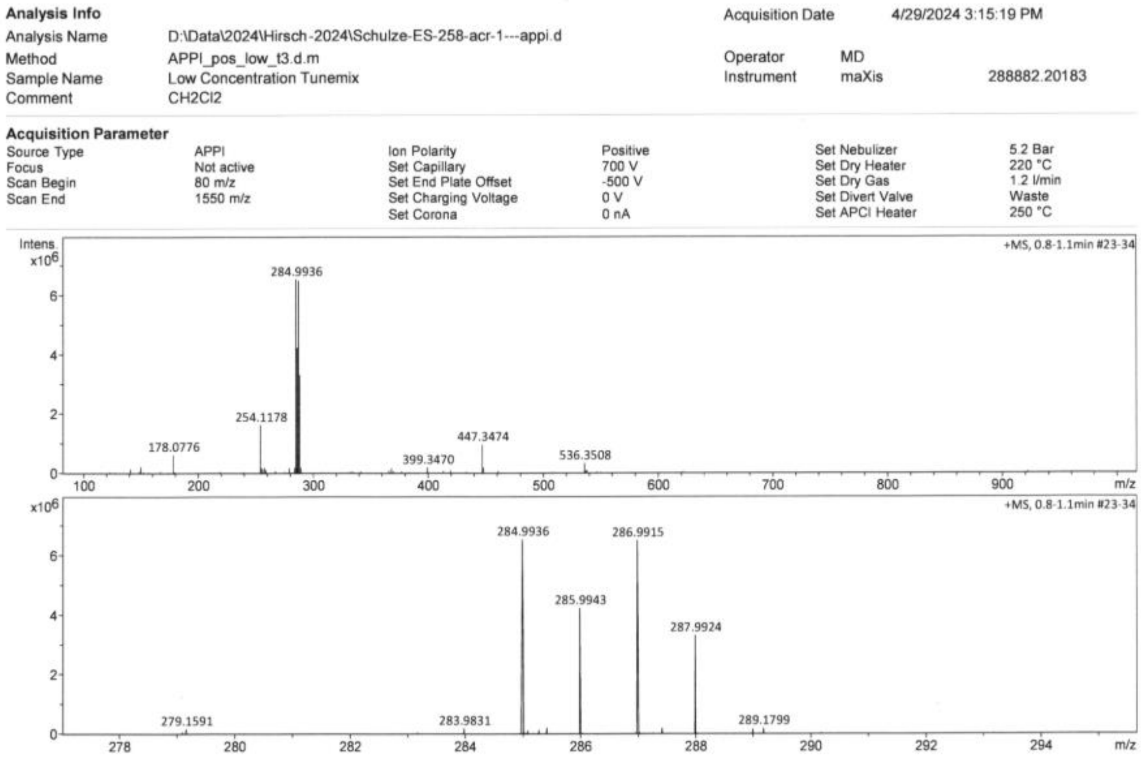

Figure S60 MS Spectrum (APPI) of **Bromo-tolane-aldehyde**

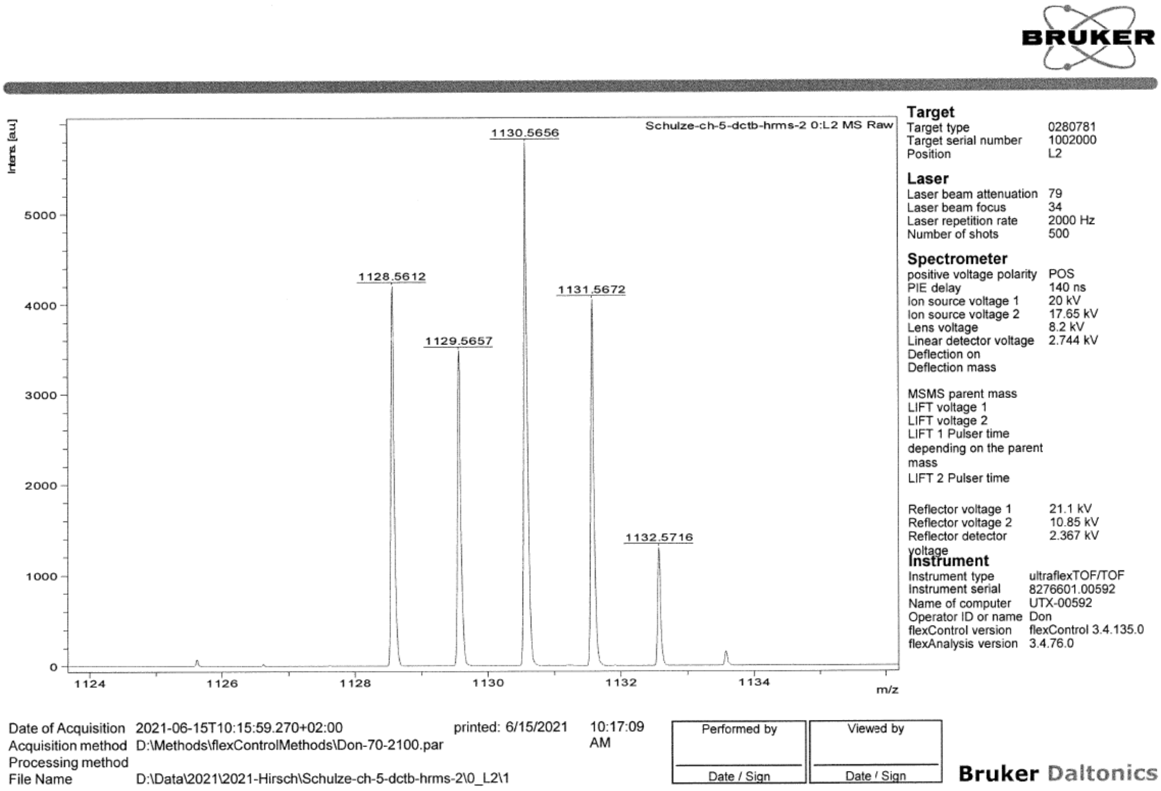

Figure S61 HRMS Spectrum (MALDI-dctb) of **1**

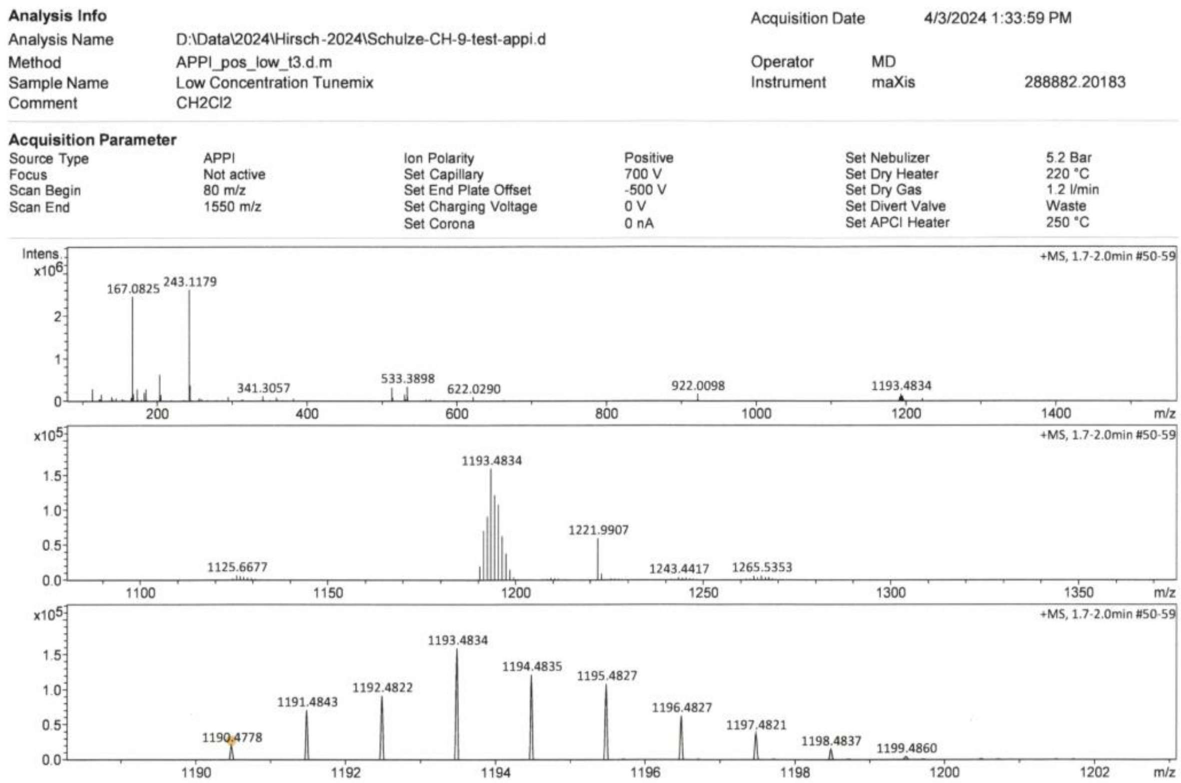

Figure S62 HRMS Spectrum (APPI) of **2**

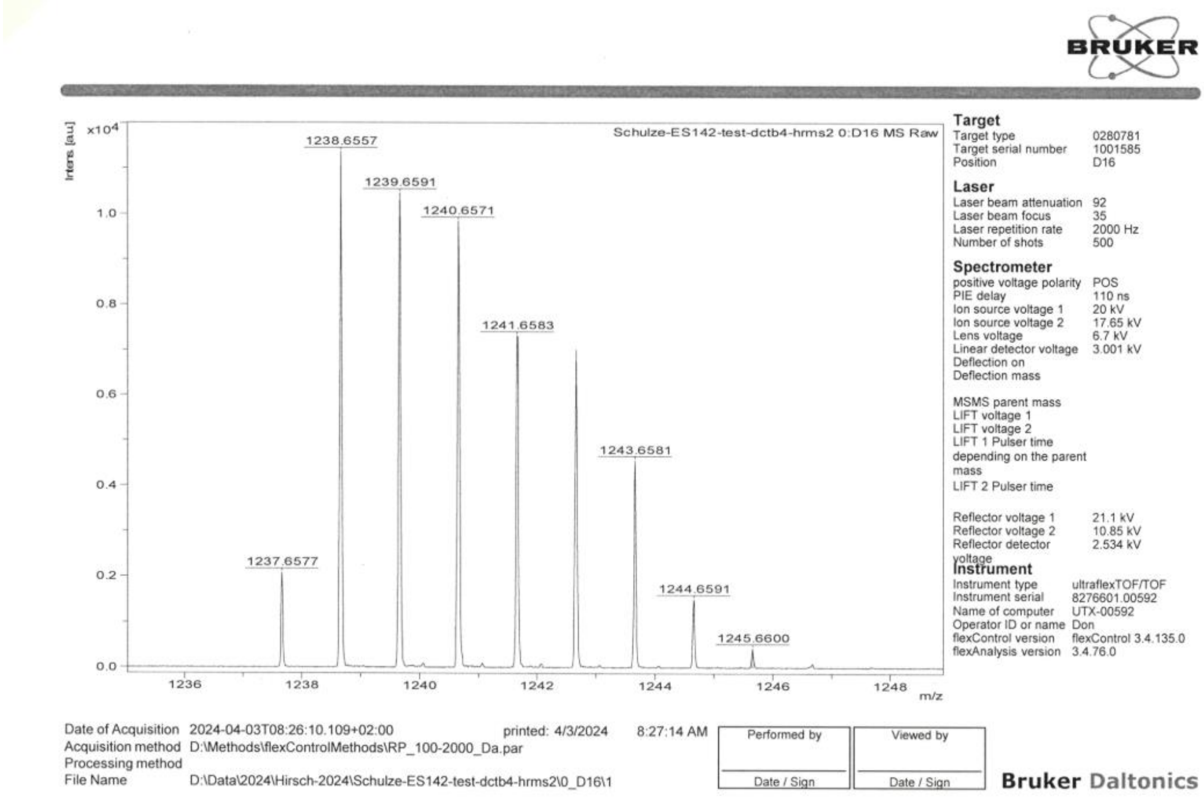

Figure S63 HRMS Spectrum (MALDI-dctb) of **3**

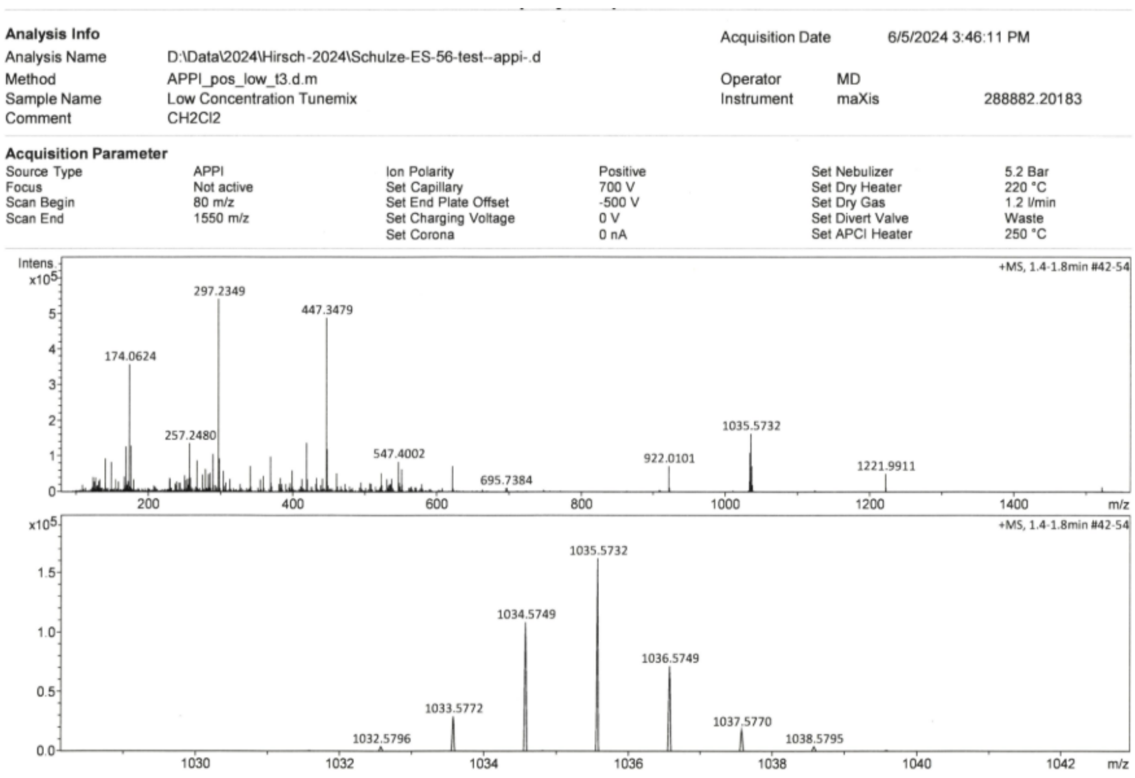

Figure S64 MS Spectrum (APPI) of **5**

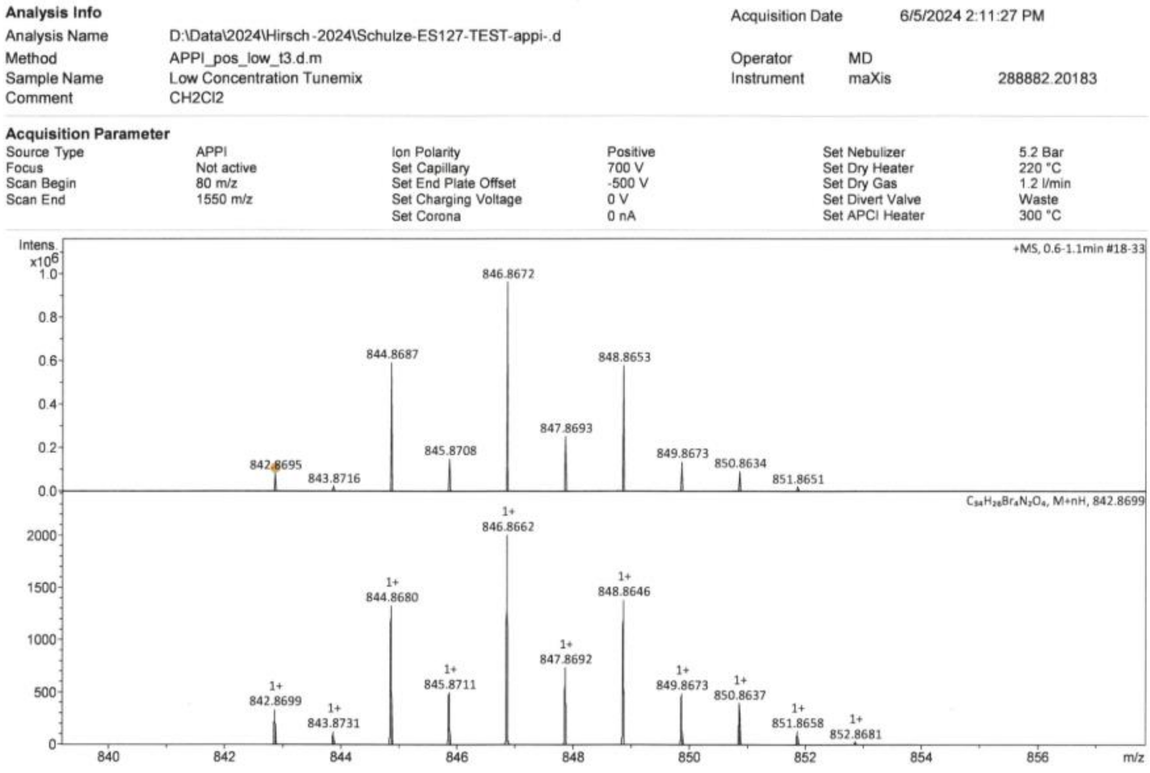

Figure S65 MS Spectrum (APPI) of **6** (Bottom simulated spectrum)

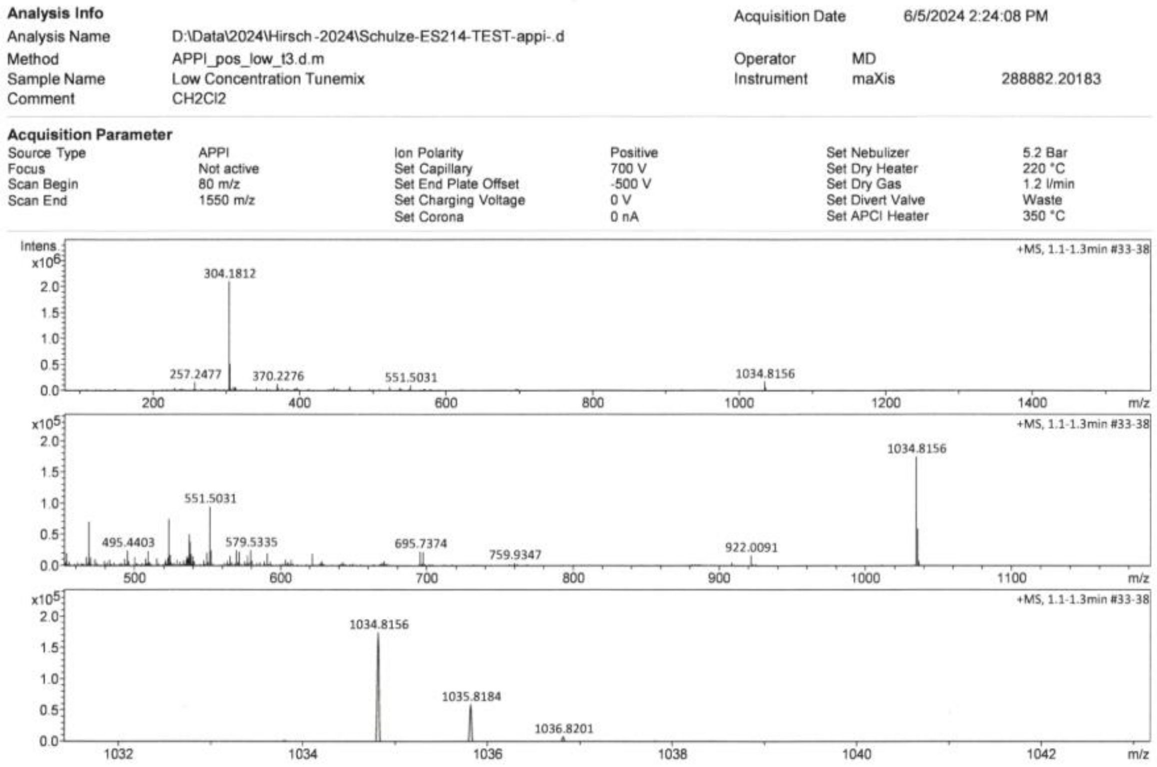

Figure S66 MS Spectrum (APPI) of **7**

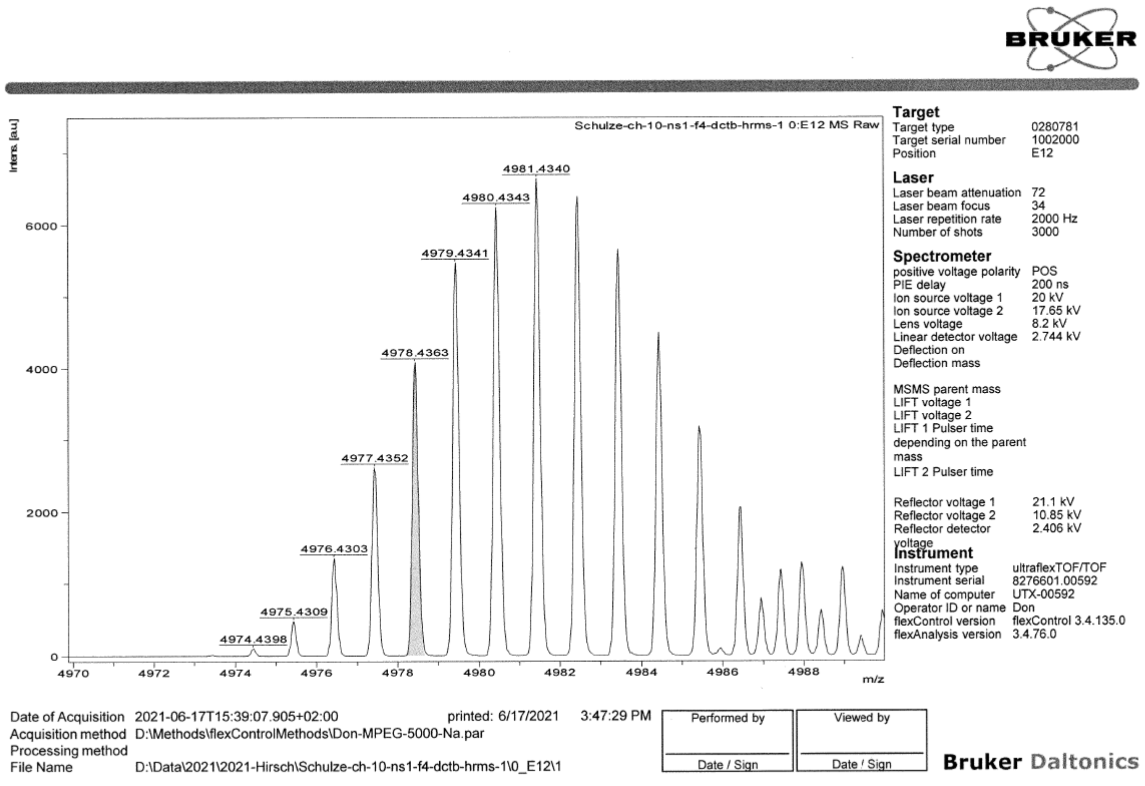

Figure S67 HRMS Spectrum (MALDI-dctb) of **8**

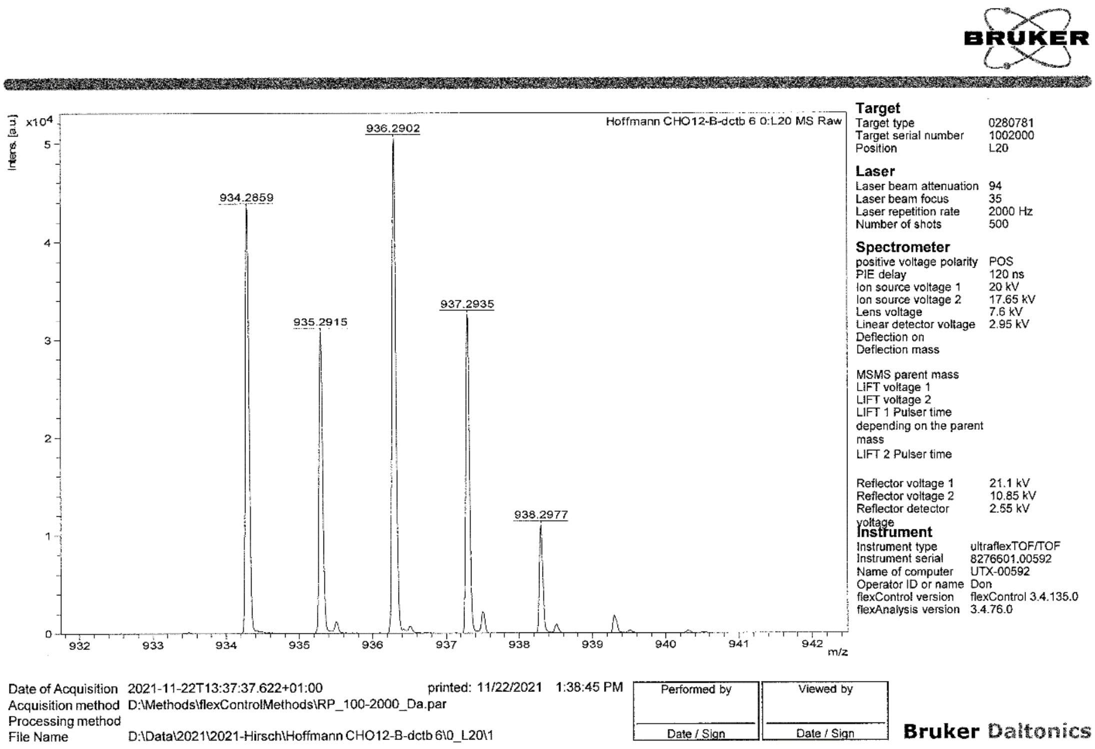

Figure S68 HRMS Spectrum (MALDI-dctb) of **9**

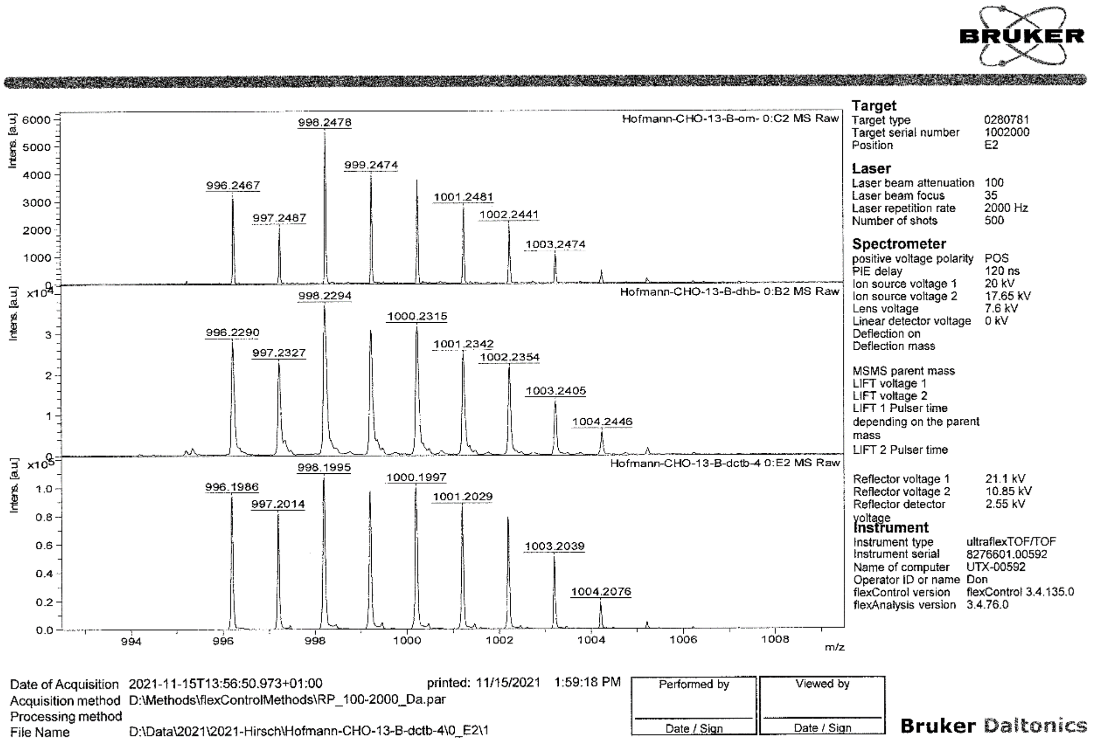

Figure S69 HRMS Spectrum (MALDI-dctb) of **10**

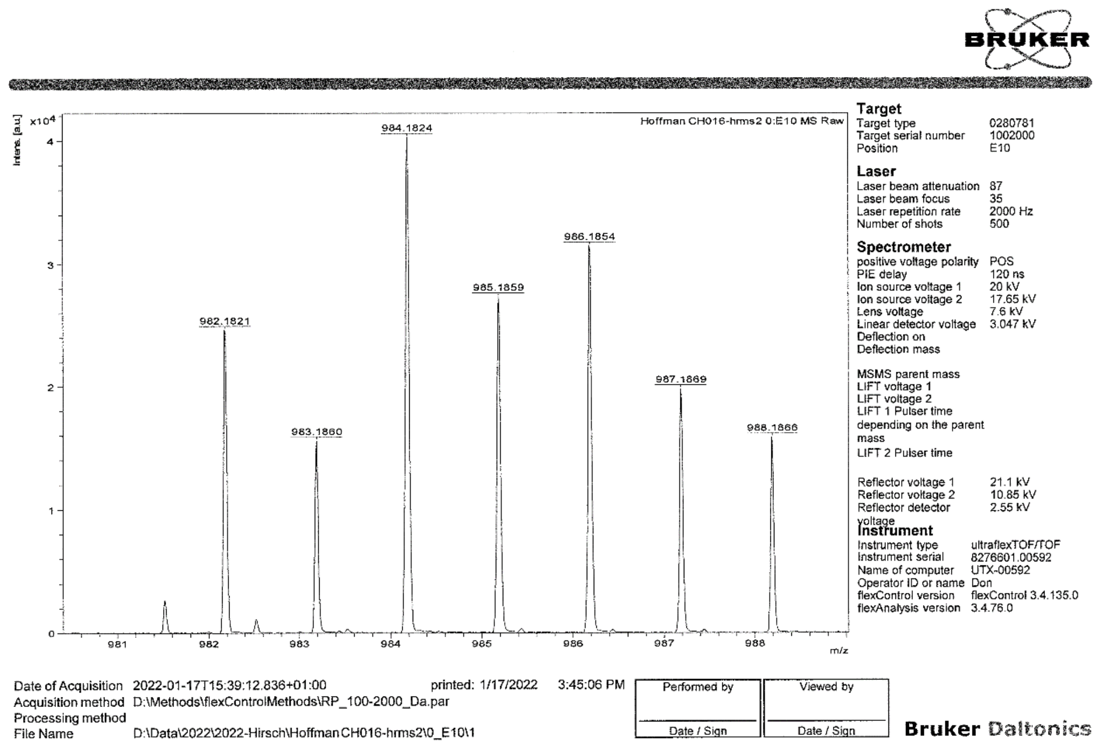

Figure S70 HRMS Spectrum (MALDI-dctb) of **11**

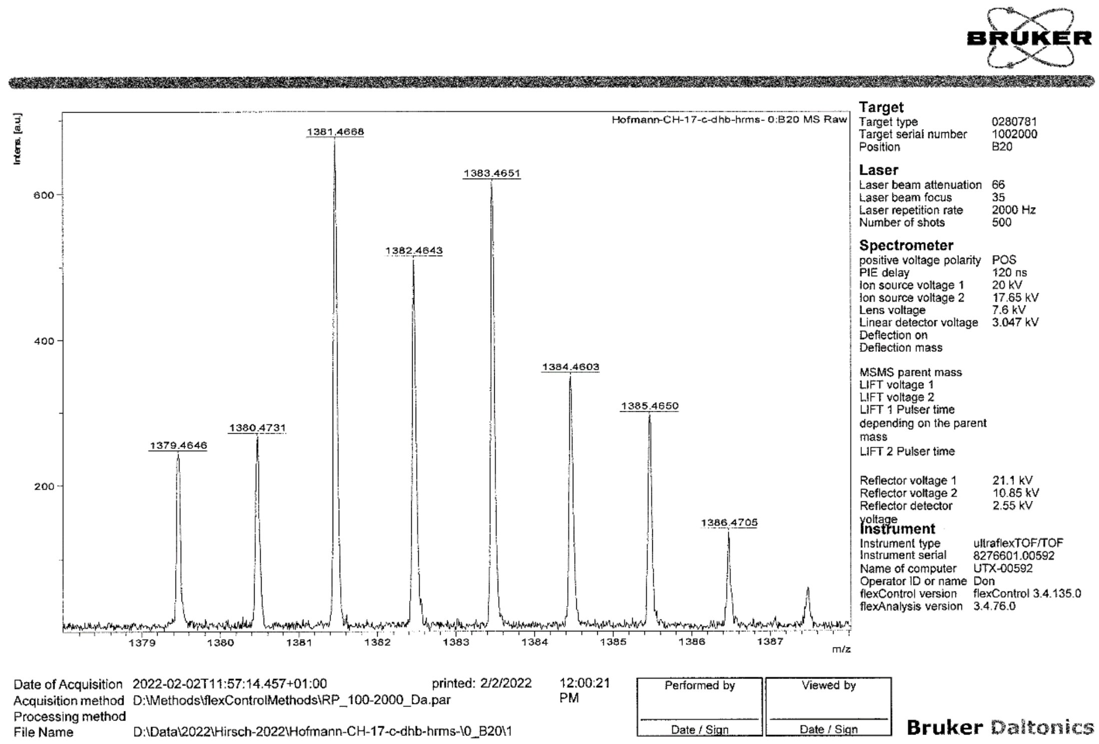

Figure S71 HRMS Spectrum (MALDI-dctb) of **12**

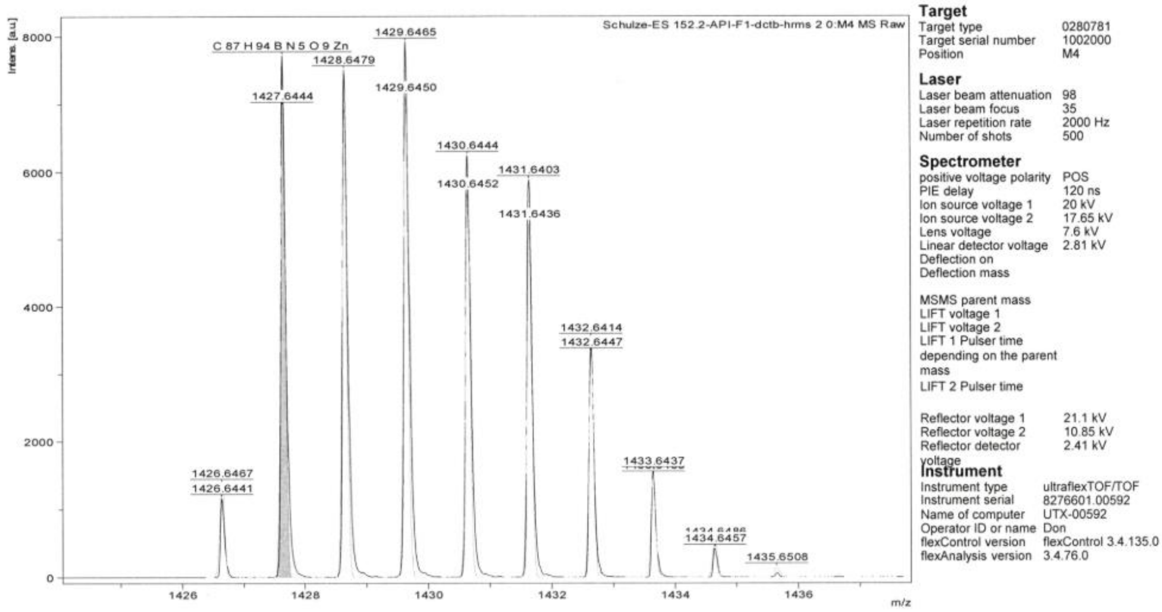

Target
Target type 0280781
Target serial number 1002000
Position M4

Laser
Laser beam attenuation 98
Laser beam focus 35
Laser repetition rate 2000 Hz
Number of shots 500

Spectrometer
positive voltage polarity POS
PIE delay 120 ns
Ion source voltage 1 20 kV
Ion source voltage 2 17.65 kV
Lens voltage 7.6 kV
Linear detector voltage 2.81 kV
Deflection on
Deflection mass

MSMS parent mass
LIFT voltage 1
LIFT voltage 2
LIFT 1 Pulser time depending on the parent mass
LIFT 2 Pulser time

Reflector voltage 1 21.1 kV
Reflector voltage 2 10.85 kV
Reflector detector voltage 2.41 kV

Instrument
Instrument type ultraflexTOF/TOF
Instrument serial 8276601.00592
Name of computer UTX-00592
Operator ID or name Don
flexControl version flexControl 3.4.135.0
flexAnalysis version 3.4.76.0

Date of Acquisition 2022-10-27T11:50:12.843+02:00 printed: 10/27/2022 12:04:34 PM
Acquisition method D:\Methods\flexControlMethods\RP_100-2000_Da.par
Processing method
File Name D:\Data\2022\Hirsch-2022\Schulze-ES 152.2-API-F1-dctb-hrms 2\0_M4\1

Performed by	Viewed by
Date / Sign	Date / Sign

Bruker Daltonics

Figure S72 HRMS Spectrum (MALDI-dctb) of **13** (Overlayed simulated spectrum)

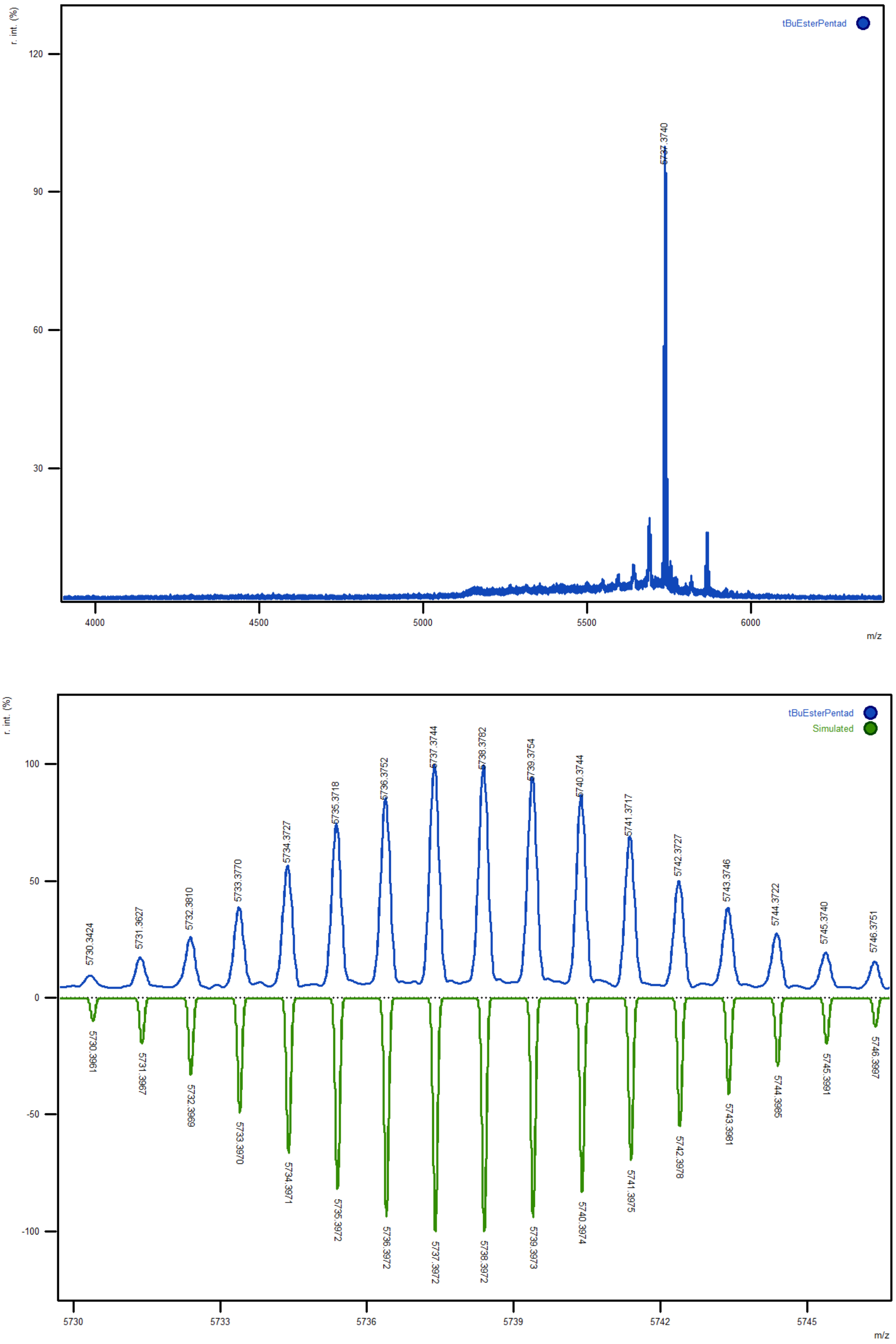

Figure S73 High resolution mass spectrum of **14** (MALDI-dctb). Top: Measured Spectrum; Bottom: Simulated spectrum. Due to the isotopic pattern and the high molecular weight of the compound, the monoisotopic peak cannot be observed well resolved in the measured spectrum, as it displays only theoretical 1.1 % relative intensity.

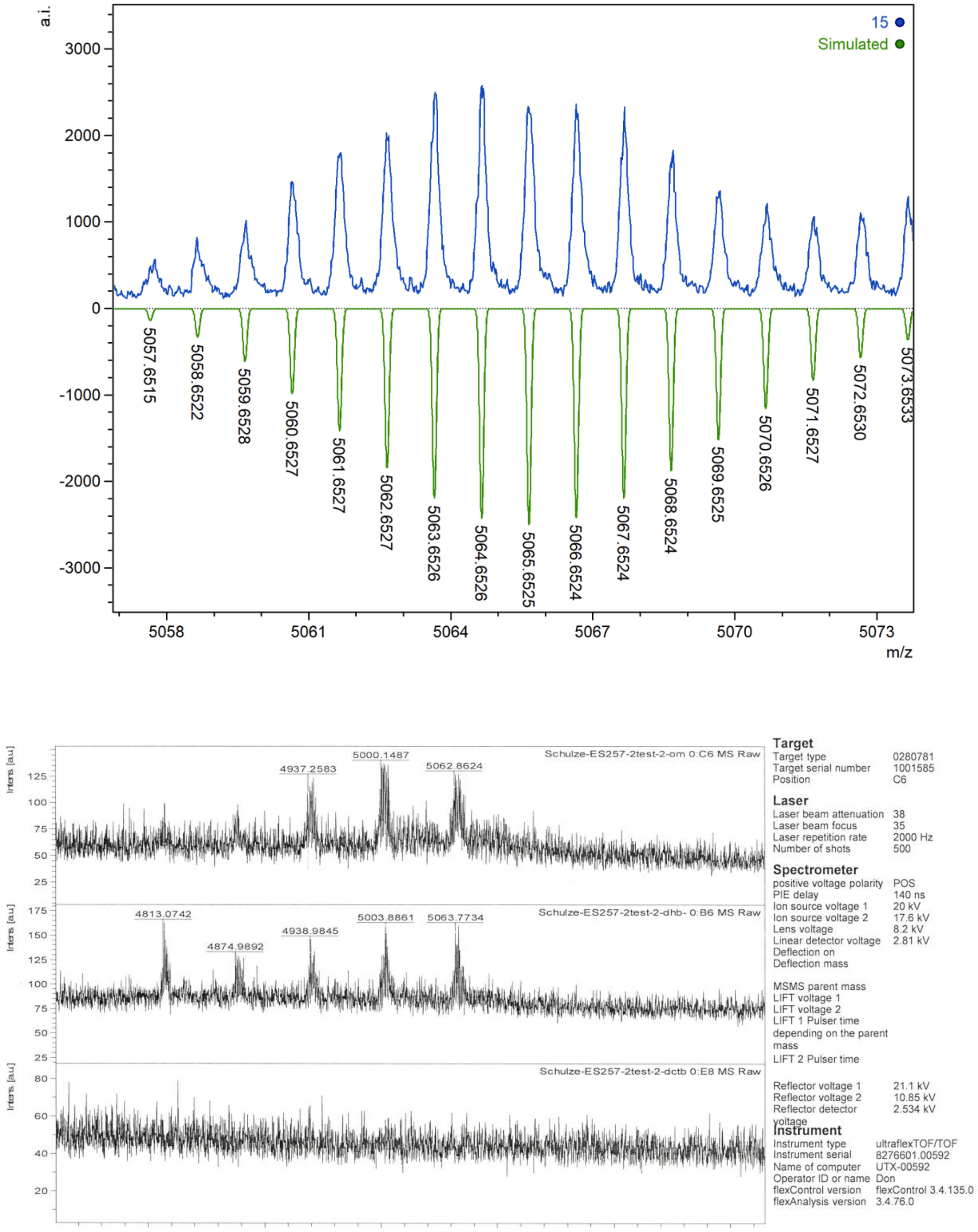

Figure S74 High resolution mass spectrum of **15** (MALDI-dctb). Top: Fully metalated pentad; Due to the isotopic pattern and the high molecular weight of the compound, the monoisotopic peak cannot be observed well resolved in the measured spectrum, as it displays only theoretical 1.9 % relative intensity; Bottom: mass spectrum of partially demetalated pentad.

7. Microscopical Imaging

The samples for STEM studies were prepared via a simple drop-casting method on lacey-ultrathin 400 mesh Cu grid (#01824, Plano). The concentrations of the aqueous solutions were 0.25 mM with 5 vol% THF and 10 vol% THF. The sample was dried on air for 1 hour. STEM experiments were performed on a double Cs-corrected Thermo Fisher Scientific Titan Themis microscope operated at 300 kV. The HAADF images were recorded with an aberration corrected probe with 15.6 mrad and collection angular range of 61–200 mrad, typical to deliver Z-contrast images. HRTEM imaging were performed using parallel illumination with the image corrector tuned to deliver resolution (information limit) better than 1Å.

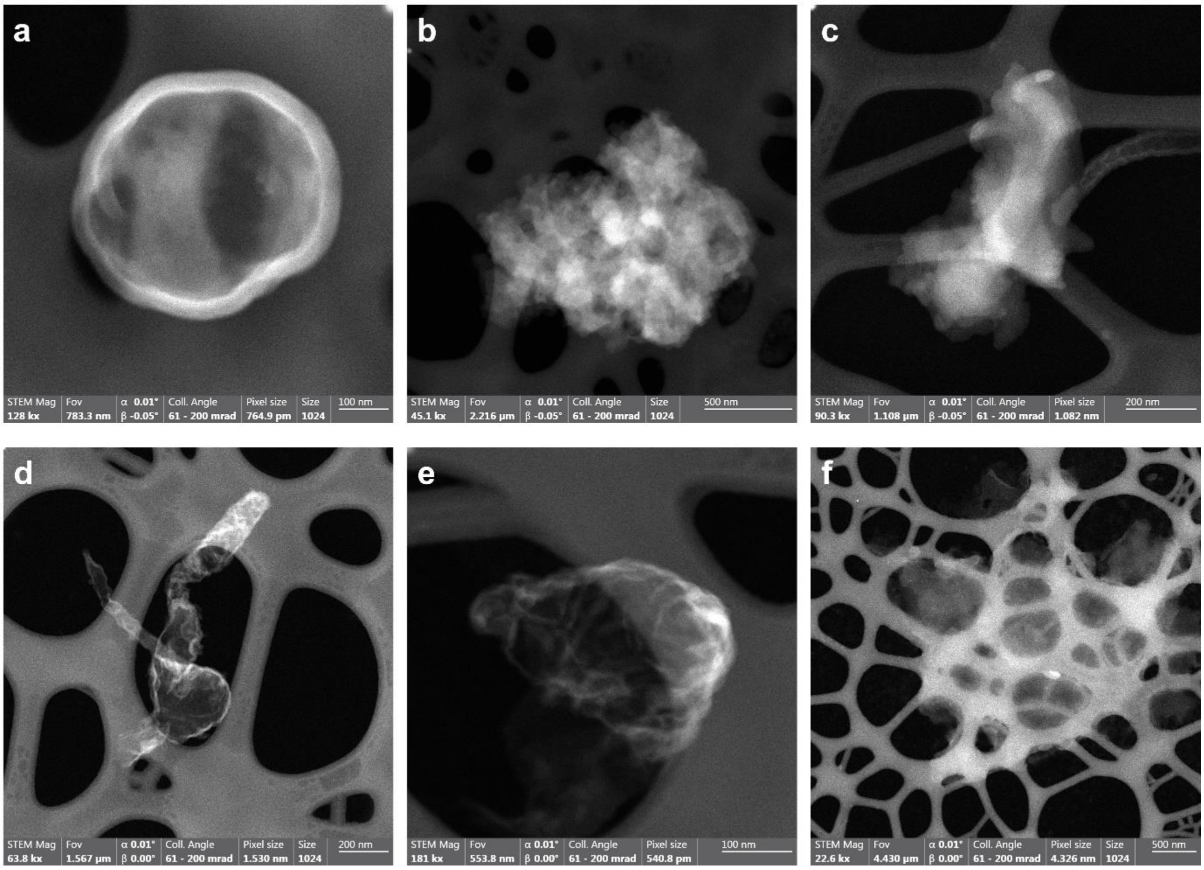

Figure S75. Various morphology of the assembled molecule 15 revealed via STEM-HAADF (Z-contrast) images. (a-c) the sodium salt of 15 (c = 0.25 mM, 5 vol% THF in H_2O, without heating) and (d-f) the sodium salt of 15 (c = 0.25 mM, 10 vol% THF in H_2O, 2 h at 60°)

The underlying lattice spacing matches very well to the expected lattice spaces of (002), (101), (004) of graphite, while the highest recorded lattice spatial frequency lie in between (110) and (112) lattice spacing of graphite. These lattice spacings might originate from the lattice motif of the PBI core, when regularly stacked, being very similar to the honeycomb structure of graphite, whereas the mismatch of the outmost ring between (110) and (112) of graphite originate from the porphyrin, having 5- and 6-membered rings that deviate from the honeycomb structure of graphite. A broad halo ring, which corresponds to ~0.7 – 1.3 nm of real space distance inside the marked (002) ring can be seen in the FFT (red dash circle in the inset of **Figure 4d**), This is could stem from the expected spacings of the PBI core to the porphyrin arms. Furthermore, the fact that multiple lattice spacings could be recorded and identified in the HRTEM image of the sample assembled in high temperature suggest a more stable, and higher degree of ordering in the molecular stacking/packing.

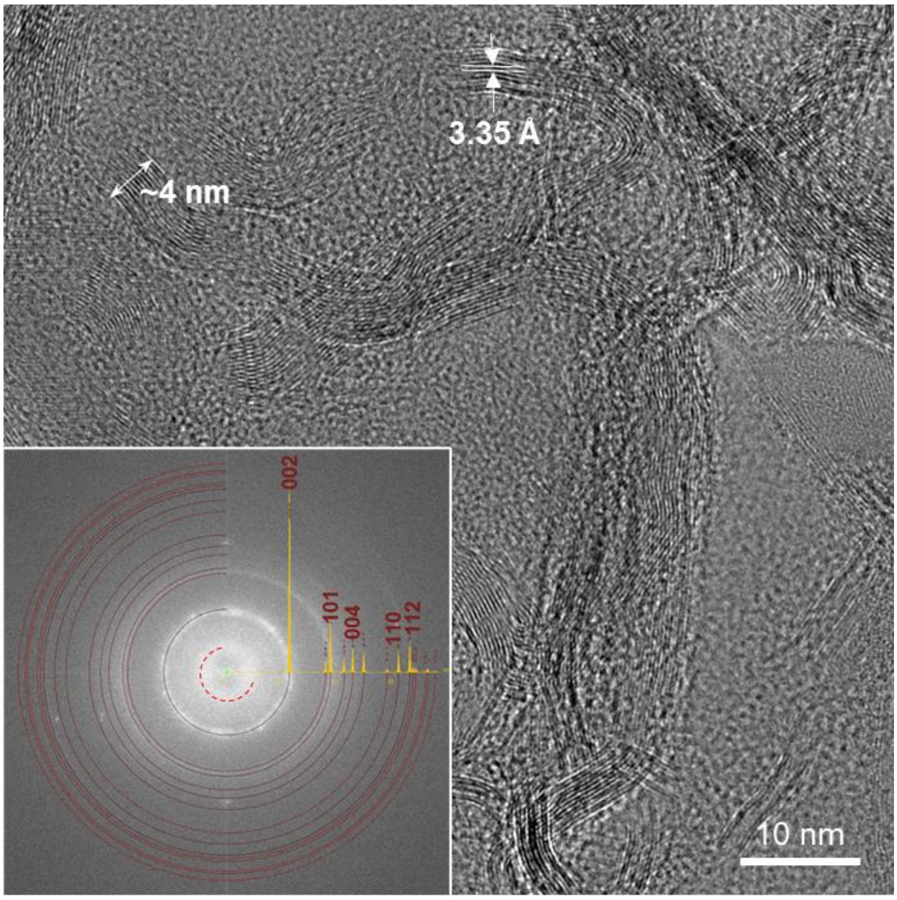

Figure S76 HRTEM image the sodium salt of **15** (c = 0.25 mM, 10 vol% THF in H_2O, 2 h at 60°) The inset is the FFT of the image with the spatial frequencies of graphite superimposed, the inner most red-dash ¾ circle indicate spatial frequency of 1 nm.

8. Theoretical Investigations

To get insights into the geometry of the amphiphile, and confirm a possible flat structure, the pentad was modelled by comparably "cheap" semiempirical methods due to the size of the molecule. For these calculations, the HyperChem^{TM} (Version: 8.0.10 for Windows) software from Hypercube Inc. was used. Structures were drawn in ChemDraw^{TM} and transferred to HyperChem^{TM}. After preliminary optimizations by force field methods, the structure was geometry optimized using semi-empirical methods (Method: SemiEmp; PM3; RMSGrad: 0.01; ConversionLimit: 0.1; IterationLimit: 50; Total Charge 0; SpinMultiplicity: 1; SpinPairing: RHF).

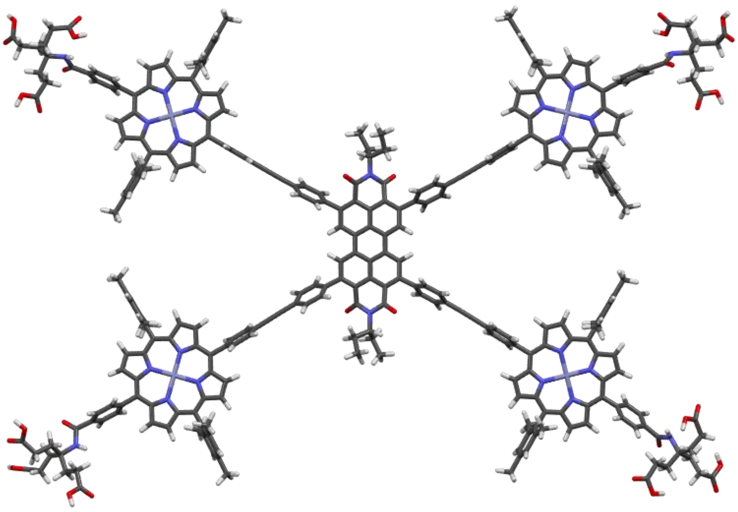

Figure S77 Theoretical model of **15** obtained from SemiEmperical (PM3) geometry optimization

9. References

[1] Christian Methfessel, Synthesis and Characterization of Amphiphilic Porphyrin-Perylene Dyads: Towards Optoelectronic Membranes in Water, Friedrich-Alexander-University Erlangen Nürnberg, **2020**.

[2] T. Luchs, A. Zieleniewska, A. Kunzmann, P. R. Schol, D. M. Guldi, A. Hirsch, *Chem. – A Eur. J.* **2021**, *27*, 5041–5050.

[3] M. M. Martin, M. Dill, J. Langer, N. Jux, *J. Org. Chem.* **2019**, *84*, 1489–1499.

[4] T. Teraoka, S. Hiroto, H. Shinokubo, *Org. Lett.* **2011**, *13*, 2532–2535.

[5] G. Battagliarin, Y. Zhao, C. Li, K. Müllen, *Org. Lett.* **2011**, *13*, 3399–3401.

[6] J. Wu, D. He, L. Zhang, Y. Liu, X. Mo, J. Lin, H. J. Zhang, *Org. Lett.* **2017**, *19*, 5438–5441.
